# Estimating the population-level non-respiratory burden of severe influenza: a systematic review of methods and estimates

**DOI:** 10.1016/j.lanhl.2026.100934

**Published:** 2026-10

**Authors:** Yun Lin, Wen Chen, Sahil Mukherjee, Justin K Cheung, Luis Alberto Garcia Valbuena, Jessica Y Wong, Vanessa Cozza, Stefano Tempia, Benjamin J Cowling

**Affiliations:** a WHO Collaborating Centre for Infectious Disease Epidemiology and Control, School of Public Health, Li Ka Shing Faculty of Medicine, The University of Hong Kong, Hong Kong Special Administrative Region, China; b Faculty of Life Sciences & Medicine, King’s College London, London, UK; c Global Influenza Programme, Epidemic and Pandemic Management, WHO, Geneva, Switzerland

## Abstract

Influenza is widely recognised as a respiratory infection, although the condition can also exacerbate or trigger serious non-respiratory outcomes that are often overlooked in burden assessments. Previous reviews have separately evaluated how methodological choices affect influenza-associated mortality and hospitalisation estimates and have covered non-respiratory outcomes in part, although these studies were restricted to select causes or severe outcomes, specific populations, or particular geographical settings. A more comprehensive synthesis focused specifically on severe non-respiratory influenza burden has been missing. To address this gap, we conducted a systematic review of 238 studies from 52 countries or regions examining both the burden of severe influenza beyond respiratory disease and the estimation methods used. We found that non-respiratory outcomes represent a substantial component of the total influenza burden, with a pronounced age gradient disproportionately affecting older adults (≥65 years). Cardiovascular events were particularly notable, contributing to more than 50 deaths and 60 hospitalisations per 100 000 older adults. Burden estimates also varied by analytical method, cause, and study period, and most evidence was concentrated in high-income settings, leaving important geographical gaps. Our findings highlight that influenza burden assessments and prevention strategies should extend beyond respiratory presentations, particularly for older populations, and emphasise upon the need for standardised methods and broader geographical coverage to generate estimates that are more comparable across studies and settings that better inform public health policy and resource allocation.

## Introduction

Influenza imposes a substantial disease burden, and accurate burden estimates are crucial for informing prevention and control programmes.^1^ Although influenza is primarily classified as a respiratory infection, it can also contribute to or exacerbate severe non-respiratory complications through systemic inflammation, increased risk of coagulation and thrombosis, and heightened metabolic demands.^2^, ^3^, ^4^ These effects can lead to acute and sometimes fatal outcomes, including cardiovascular events, strokes, or acute decompensation of chronic conditions, particularly in older adults (≥65 years) and individuals with pre-existing health conditions.^5^, ^6^

Estimating the population-level impact of influenza requires approaches beyond laboratory-confirmed cases, which capture only a fraction of infections because of underdetection, variable health care-seeking behaviour, and imperfect testing sensitivity.^7^ Statistical and mathematical models—ie, excess burden approaches—address this issue by estimating the population-level impact of influenza while accounting for background trends and other confounders.^8^ These methods have substantially improved estimates of influenza-associated respiratory hospitalisation and mortality burdens.^9^, ^10^, ^11^ However, the impact of influenza beyond respiratory disease remains under-represented because hospital records or death certificates usually capture the immediate clinical event rather than influenza as the underlying trigger^3^, ^12^ and low laboratory confirmation and time delay between infection and secondary outcomes further obscure this link and contribute to underestimation of the full burden of influenza.^2^, ^3^

Although earlier reviews have separately evaluated how methodological choices affect influenza-associated mortality and hospitalisation estimates and have covered non-respiratory outcomes in part,^13^, ^14^ these studies were restricted to select causes or severe outcomes, did not focus specifically on non-respiratory burden, or were restricted to specific geographical settings or demographic subgroups.^15^, ^16^ Therefore, a more comprehensive synthesis specifically focusing on the non-respiratory burden of severe influenza is needed to improve understanding of this evidence base and to inform better-designed, better-reported, and more targeted future studies. Thus, we conducted a systematic review of peer-reviewed studies that used statistical or modelling approaches to estimate population-level influenza-associated severe disease burden beyond respiratory-only outcomes. We aimed to summarise and compare the methodologies used, highlight the estimated non-respiratory burden, and assess the influence of methodological choices on reported estimates.

## Methods

### Search strategy and selection criteria

This systematic review was registered with PROSPERO (CRD420250636210), conducted according to established guidance for systematic reviews of observational epidemiological studies reporting prevalence and incidence data,^17^ and reported following PRISMA guidelines.^18^ We searched MEDLINE (PubMed) and Embase for peer-reviewed articles published from the date of database inception up to Aug 18, 2026, using combinations of search terms to identify studies reporting non-respiratory severe burden estimates associated with influenza virus infection (full search strategy in the appendix pp 1–2). The search terms included both specific causes recognised as influenza-related, such as respiratory and cardiovascular, different cardiovascular diseases (including both broad categories and specific conditions such as myocardial infarction and ischaemic heart diseases), and diabetes,^19^, ^20^, ^21^ and broader terms such as “non-respiratory” and “all-cause”. To capture the broader spectrum of severe burden, we also included terms for outcomes beyond hospitalisation and mortality, such as medical attendance and long-term complications.

No language restriction was applied to the retrieved articles. Additional relevant articles were manually retrieved from the reference lists of papers identified from the two databases. Two authors (YL and WC) independently screened all titles, abstracts, and full texts for eligibility, and any disagreements were resolved by the third author (JKC).

Eligible articles were those that used statistical or modelling approaches to estimate population-level influenza-associated severe disease burden involving non-respiratory outcomes (full eligibility criteria in the appendix pp 2–3). Consistent with our focus on population-level impact rather than individual-level clinical seriousness,^8^ studies reporting only laboratory-confirmed influenza outcomes were excluded. Wherever multiple publications reported the same underlying study, duplicate reports were identified and the most comprehensive report was retained for analysis, according to the predefined criteria described in the appendix (p 3).

### Data analysis

All data were independently extracted by two authors (YL and WC) using a standardised form. The extracted information included study characteristics (first author, location, and time period), the causes and outcomes studied, methodological details (study definitions, data sources, covariates, statistical models, influenza activity proxies or epidemic thresholds, and uncertainty measures), and reported estimates of influenza-associated severe burden. Missing methodological details were supplemented from cited references when necessary.

Estimates were collected for the overall population and stratified by age group, when available. We prespecified our analytical age groups as children (<5 years), adolescents (5–19 years), adults (20–44 and 45–64 years), and older adults (≥65 years), on the basis of age bands most commonly reported in existing influenza burden studies. Reported age bands were assigned to the analytic age group with which they shared the greatest overlap (appendix pp 4–5). We focused on comparing rates instead of absolute numbers to minimise the impact of changing population sizes. When studies provided only count data, we obtained the relevant population size to calculate rates. If annual estimates were reported for multiple years, we calculated the average rate across the study period (appendix pp 9–10).

Risk of bias for all included studies was assessed using the ROBINS-E tool^22^ (details in the appendix pp 3–4).

We first summarised the characteristics of the included studies, such as causes, outcomes, age groups, study regions, study periods, and statistical methods. Specifically, we extracted key methodological features, such as the statistical models used, proxies for influenza activity or epidemic threshold, and other model covariates. Statistical modelling approaches were classified by how they linked outcomes to influenza activity and estimated excess burden as follows: regression models using influenza activity proxies to statistically attribute population-level outcomes to influenza activity; regression models without influenza activity proxies (including Serfling-type models) that estimate influenza-associated burden from excess rates above a modelled baseline; rate-difference methods that directly subtract observed outcome rates during non-epidemic baseline periods from those during influenza epidemic periods; and other methods (detailed classification in the appendix pp 8–9). Methodological features were compared across studies, specifically the use of different influenza activity proxies in regression models incorporating them, and methods for defining baseline or epidemic periods in regression models without proxies and rate-difference approaches.

To contextualise non-respiratory burden relative to respiratory outcomes, we additionally extracted respiratory estimates (primarily pneumonia and influenza and broad respiratory categories) from the included studies that reported both respiratory and non-respiratory outcomes (appendix pp 11–13).

Forest plots were constructed to present age-specific estimates for influenza-associated hospitalisation and mortality by cause, whereas boxplots were used to compare estimates across methodological categories. Statistical heterogeneity of the estimates among studies was assessed using the *I*^2^ statistic.^23^ To evaluate the impact of methodological choices on estimates, we conducted random-effects meta-regression analyses for mortality and hospitalisation estimates, which had the most available data. Of the 1451 available estimates, 512 (35·3%) had a reported SE or CI. For the remainder, SEs were imputed using a linear regression model fitted to estimates with reported uncertainty, with the absolute rate value, age group, and cause category as predictors (appendix pp 10–11).

For each outcome, we fitted a random-effects meta-regression model using restricted maximum likelihood to estimate between-study variance, with study-level covariates including age group, WHO region (selected over World Bank income-level classification based on model fit; appendix p 11), cause of death or hospitalisation (eg, cardiovascular disease or diabetes), seasonal or pandemic period or both, and modelling approach. As individual studies could contribute multiple estimates (eg, across age groups or causes), we conducted a sensitivity analysis using study-level cluster-robust variance estimation to assess the impact of non-independence on our results (appendix pp 10–11). We further extended the models to include pneumonia and influenza and broad respiratory categories as reference groups to contextualise non-respiratory estimates relative to respiratory outcomes (appendix pp 11–13).

All analyses were conducted using R version 4.4.0.

## Results

We identified 238 publications that met the inclusion criteria^7^, ^12^, ^19^, ^20^, ^21^, ^24^, ^25^, ^26^, ^27^, ^28^, ^29^, ^30^, ^31^, ^32^, ^33^, ^34^, ^35^, ^36^, ^37^, ^38^, ^39^, ^40^, ^41^, ^42^, ^43^, ^44^, ^45^, ^46^, ^47^, ^48^, ^49^, ^50^, ^51^, ^52^, ^53^, ^54^, ^55^, ^56^, ^57^, ^58^, ^59^, ^60^, ^61^, ^62^, ^63^, ^64^, ^65^, ^66^, ^67^, ^68^, ^69^, ^70^, ^71^, ^72^, ^73^, ^74^, ^75^, ^76^, ^77^, ^78^, ^79^, ^80^, ^81^, ^82^, ^83^, ^84^, ^85^, ^86^, ^87^, ^88^, ^89^, ^90^, ^91^, ^92^, ^93^, ^94^, ^95^, ^96^, ^97^, ^98^, ^99^, ^100^, ^101^, ^102^, ^103^, ^104^, ^105^, ^106^, ^107^, ^108^, ^109^, ^110^, ^111^, ^112^, ^113^, ^114^, ^115^, ^116^, ^117^, ^118^, ^119^, ^120^, ^121^, ^122^, ^123^, ^124^, ^125^, ^126^, ^127^, ^128^, ^129^, ^130^, ^131^, ^132^, ^133^, ^134^, ^135^, ^136^, ^137^, ^138^, ^139^, ^140^, ^141^, ^142^, ^143^, ^144^, ^145^, ^146^, ^147^, ^148^, ^149^, ^150^, ^151^, ^152^, ^153^, ^154^, ^155^, ^156^, ^157^, ^158^, ^159^, ^160^, ^161^, ^162^, ^163^, ^164^, ^165^, ^166^, ^167^, ^168^, ^169^, ^170^, ^171^, ^172^, ^173^, ^174^, ^175^, ^176^, ^177^, ^178^, ^179^, ^180^, ^181^, ^182^, ^183^, ^184^, ^185^, ^186^, ^187^, ^188^, ^189^, ^190^, ^191^, ^192^, ^193^, ^194^, ^195^, ^196^, ^197^, ^198^, ^199^, ^200^, ^201^, ^202^, ^203^, ^204^, ^205^, ^206^, ^207^, ^208^, ^209^, ^210^, ^211^, ^212^, ^213^, ^214^, ^215^, ^216^, ^217^, ^218^, ^219^, ^220^, ^221^, ^222^, ^223^, ^224^, ^225^, ^226^, ^227^, ^228^, ^229^, ^230^, ^231^, ^232^, ^233^, ^234^, ^235^, ^236^, ^237^, ^238^, ^239^, ^240^, ^241^, ^242^, ^243^, ^244^, ^245^, ^246^, ^247^, ^248^, ^249^, ^250^, ^251^, ^252^, ^253^, ^254^, ^255^, ^256^ (figure 1; appendix pp 14–38). Most studies were published within the past five to 15 years, with a notable increase after the 2009 influenza pandemic (appendix p 39). The included studies reported estimates from 52 countries or regions, covering approximately 60% of the world’s population, with a small number of studies focusing on global or supraregional estimates (appendix pp 14–38). Most studies were conducted in the European region (95 [40%] of 238), the region of the Americas (71 [30%]), and the Western Pacific region (71 [30%]), with the majority based in high-income countries (190 [80%]; figure 2).

**Figure 1:**
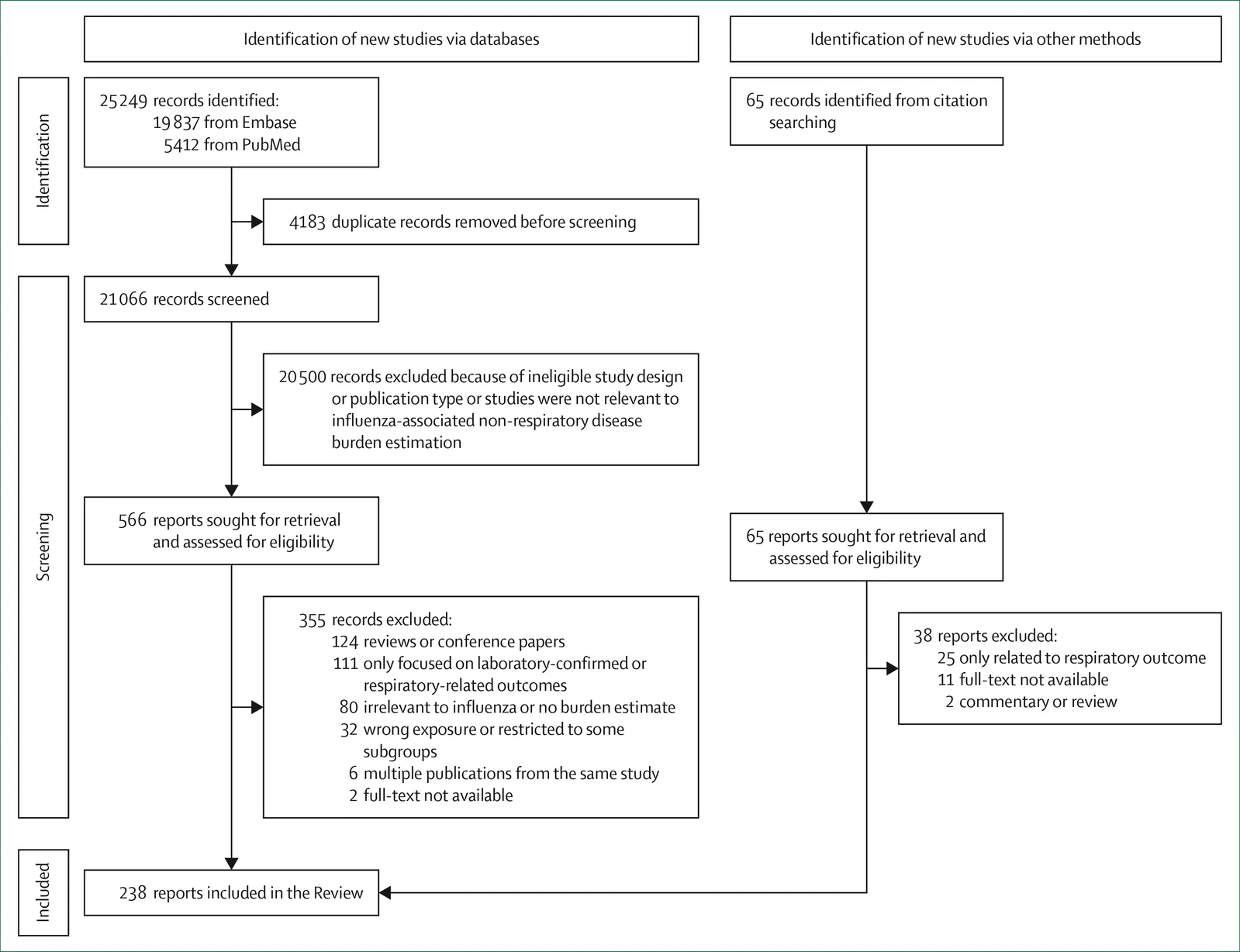
PRISMA flow diagram of study selection process Summary of identification, screening, eligibility assessment, and inclusion of studies in the systematic review.

**Figure 2:**
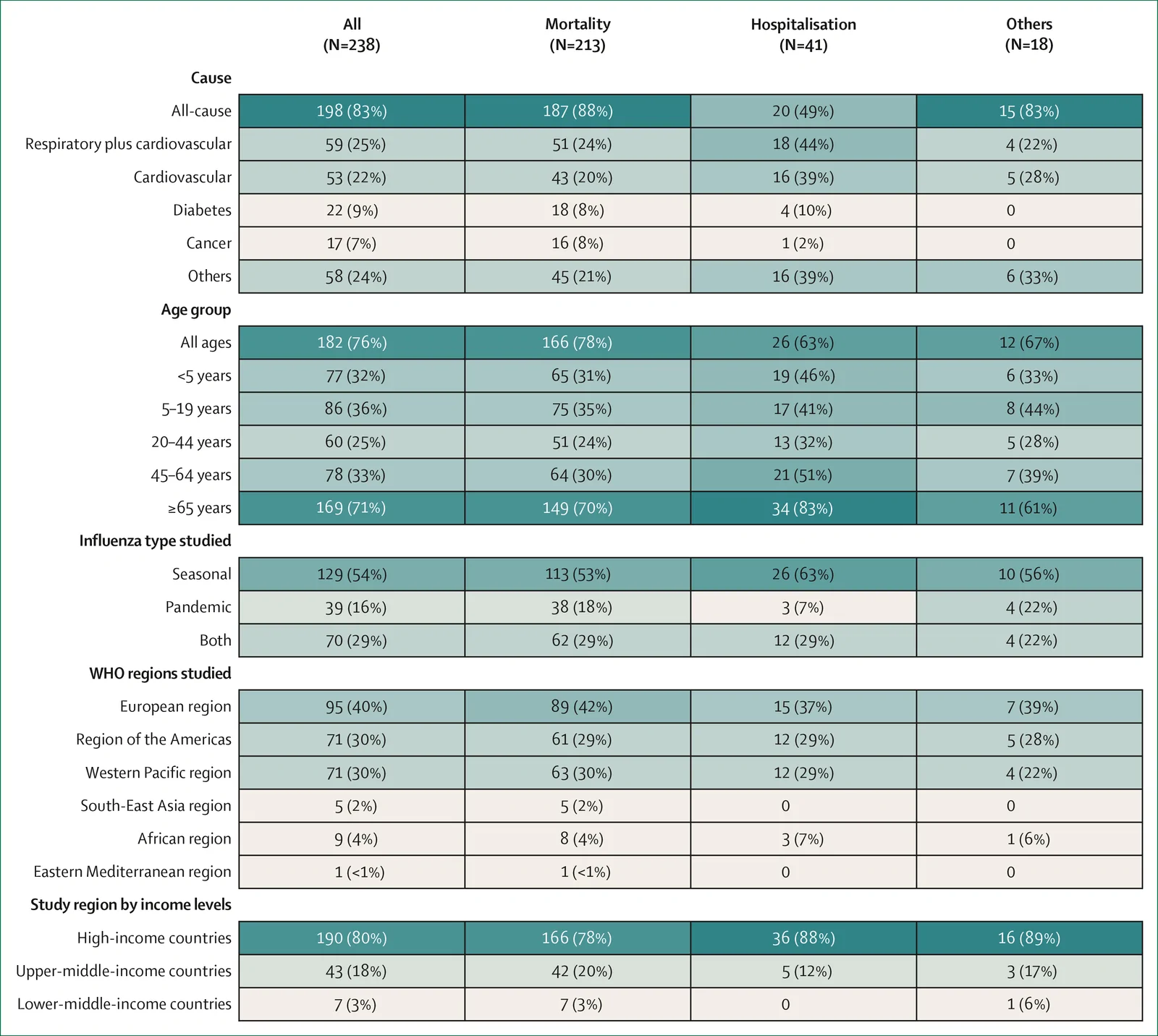
Characteristics of included studies estimating non-respiratory severe influenza disease burden The columns provide data for all studies, and subsets focusing on mortality, hospitalisation, and other outcomes. Other outcomes include intensive care unit admission or hospitalisation with ventilator use, influenza-associated complications, emergency department visits, outpatient or general practitioner consultations, medically attended illness, antibiotic prescriptions, years of life lost, sickness insurance claims, and absenteeism (see the appendix pp 7–8 for a detailed summary). Percentages within each category are shown in parentheses. Cell colour intensity indicates the proportion within each category, with darker shading representing higher proportions. Included studies could report on more than one outcome.

Various age group categorisations were used, and older adults (169 [71%] of 238) were the most frequently studied group (figure 2). Of the included studies, the majority reported mortality (213 [89%] of 238) or hospitalisation (41 [17%]), with all-cause (198 [83%]), respiratory and cardiovascular (59 [25%]), cardiovascular (53 [22%]), diabetes (22 [9%]), and cancer-related (17 [7%]) being the most studied causes. Data sources were largely homogeneous within each outcome category: mortality outcomes nearly universally used death certificate or official death registration data, whereas hospitalisation studies used hospital discharge or administrative databases, with outcomes generally defined using the underlying cause of death or primary discharge diagnosis, usually based on ICD-9 or ICD-10 classifications, or both. A small number of studies (n=18) reported other burden indicators, including severe disease (eg, intensive care unit admission), health-care use (eg, emergency department or outpatient visits), and societal burden (eg, years of life lost or absenteeism), largely focusing on all-cause, respiratory and cardiovascular, and cardiovascular outcomes (figure 2, appendix pp 7–8, 14–38).

Most studies were rated as low overall risk of bias (117 [49%] of 238), whereas 66 (28%) had some concerns, 34 (14%) were rated as high risk, and 21 (9%) were rated as very high risk (appendix p 40). Among studies rated as having some concerns or higher risks, exposure measurement was the most frequent source of concern, followed by missing data and confounding (appendix p 4).

Different modelling techniques were used to estimate influenza-associated severe burden (appendix pp 14–38). Most studies applied a single main statistical approach, whereas 17 reported estimates from more than one method. Overall, 117 studies used regression models incorporating an influenza activity proxy, 73 used regression models without a proxy (eg, Serfling-type models), and 38 used a rate-difference approach. Within each method category, most studies focused on mortality, with fewer addressing hospitalisations or other outcomes, particularly among studies using regression models without proxy (table). In addition to these three main approaches, 28 studies used other methods such as the moving average approach, relative mortality distribution models, multiplier approaches, and so forth (appendix pp 9, 14–38).

**Table:** Summary of methodological details of three main statistical approaches (regression model with Influenza proxy, regression model without Influenza proxy, and rate-difference approach) used to estimate non-respiratory influenza-associated severe disease burden

	Regression model with proxy	Regression model without proxy	Rate-difference approach
Number of studies	117	73	38
Outcomes∗			
Mortality	100 (85%)	72 (99%)	32 (84%)
Hospitalisation	27 (23%)	1 (1%)	9 (24%)
Others†	7 (6%)	3 (4%)	5 (13%)
Cause∗			
All cause	89 (76%)	66 (90%)	35 (92%)
Respiratory plus cardiovascular	43 (37%)	9 (12%)	7 (18%)
Cardiovascular diseases‡	47 (40%)	11 (15%)	9 (24%)
Diabetes	10 (8%)	8 (11%)	1 (3%)
Cancer	7 (6%)	5 (7%)	1 (3%)
Others	25 (21%)	4 (5%)	4 (10%)
Influenza period			
Seasonal	69 (59%)	30 (41%)	22 (58%)
Pandemic	2 (2%)	23 (31%)	12 (32%)
Both	46 (39%)	20 (27%)	4 (10%)
Study region (by WHO region)∗			
EUR	36 (31%)	37 (51%)	21 (55%)
AMR	25 (21%)	32 (44%)	13 (34%)
WPR	51 (44%)	7 (10%)	9 (24%)
SEAR	3 (3%)	1 (1%)	1 (3%)
AFR	6 (5%)	2 (3%)	2 (5%)
EMR	0	1 (1%)	0
Study region (by World Bank income level)∗§			
High-income	90 (77%)	56 (77%)	34 (89%)
Upper-middle-income	25 (21%)	13 (18%)	3 (8%)
Lower-middle-income	3 (3%)	1 (1%)	3 (8%)
Consideration of time¶			
Linear or polynomial terms and sinusoidal function	58 (50%)	48 (66%)	··
Linear or polynomial terms	21 (18%)	3 (4%)	··
Spline function	22 (19%)	4 (5%)	··
Statistical model¶			
Linear	23 (20%)	49 (67%)	··
Poisson	57 (49%)	17 (23%)	··
Negative binomial	26 (22%)	2 (3%)	··
Influenza activity proxy			
Proportion positive	44 (38%)	··	··
Influenza-like illness × proportion positive	24 (20%)	··	··
Laboratory-confirmed case count or incidence	17 (14%)	··	··
General practitioner or outpatient influenza-like illness count or rate	20 (17%)	··	··
Influenza hospitalisation or mortality	7 (6%)	··	··
Others	5 (4%)	··	··
Epidemic threshold¶			
Influenza burden exceeding a specified threshold	··	48 (66%)	7 (18%)
Influenza case count exceeding a specified threshold	··	4 (5%)	15 (39%)
Other model considerations			
Activity of respiratory syncytial virus	39 (33%)	··	··
Meteorological factors	55 (47%)	··	··
Lag between influenza activity and outcome	62 (53%)	··	··
Uncertainty estimation¶			
Bootstrapping	39 (33%)	5 (7%)	1 (3%)
Using standard error||	22 (19%)	11 (15%)	5 (13%)
Others∗∗	36 (31%)	31 (42%)	14 (37%)

AFR=African region. AMR=Region of the Americas. EMR=Eastern Mediterranean region. EUR=European region. SEAR=South-East Asia region. WPR=Western Pacific region.

∗ A single study might have reported multiple outcomes, causes, or locations, so category subtotals might exceed the total number of studies.

† Other burden indicators beyond hospitalisation and mortality, including intensive care unit admission or hospitalisation with mechanical ventilator use, influenza-associated complications, emergency department visits, outpatient or general practitioner consultations, medically attended illness, antibiotic prescriptions, years of life lost, sickness insurance claims, and absenteeism (see appendix pp 7–8 for a detailed summary).

‡ Includes broad categories of cardiovascular diseases and specific conditions such as heart disease, cerebrovascular disease, myocardial infarction, and ischemic heart disease.

§ Some studies reported multicountry or regional estimates that a single World Bank income-level classification might not be available.

¶ Some studies might have used methods not listed in the table, so subtotals in each category might be less than the total number of studies.

|| Refers to uncertainty estimated based on standard errors of regression model coefficients (assuming a normal approximation).

∗∗ Refers to other uncertainty estimation methods (eg, Bayesian approaches or other methods not specified).

Among studies using regression models with an influenza activity proxy, majority (58 [50%] of 117), controlled for temporal trends and seasonality by including linear or polynomial terms, or both, and sinusoidal functions of time. The most frequently used regression models were Poisson (57 [49%] of 117) and negative binomial (26 [22%] of 117). The most common influenza activity proxy was the proportion of samples testing positive for influenza (44 [38%] of 117). Many studies adjusted for the activity of respiratory syncytial virus (39 [33%] of 117) or meteorological factors such as temperature and humidity (55 [47%] of 117). In about half of these studies (62 [53%] of 117), different time lags were assumed between influenza activity and disease outcomes (table). Baseline burden was usually calculated by setting influenza activity to zero or removing the viral term from the model, with most studies then estimating excess burden either by subtracting this modelled baseline from the predicted burden at observed influenza levels or from the observed burden itself. A smaller group of studies calculated the burden directly as the product of the influenza activity indicator and corresponding regression coefficient instead.

For the 73 studies using regression models without an influenza activity proxy, 48 (66%) used Serfling-type approaches that controlled for temporal trends and seasonality using linear or polynomial terms, or both, and sinusoidal functions and linear regression was the most common model (49 [67%]). Most of these studies defined the influenza period as the time when the influenza burden exceeded a specified epidemic threshold, allowing them to distinguish between epidemic and non-epidemic periods. Excess burden was then estimated by subtracting baseline burden from the observed burden during epidemic periods. In comparison, the 38 studies that used a rate-difference approach typically calculated excess burden by comparing burdens during epidemic periods with those during non-epidemic periods (table).

In general, regression models without an influenza activity proxy and the rate-difference approach were more commonly used to estimate all-cause outcomes or to generate estimates for pandemic periods (table). Studies across all three approaches were predominantly from high-income settings and concentrated in the European region and Americas, with the Western Pacific region also contributing a substantial share, particularly for studies using regression models with a proxy (table). A growing number of studies, mainly from high-income and upper-middle-income countries or regions, have used regression models that incorporate influenza activity (appendix p 41), and these models increasingly include type-specific influenza proxies (27% pre-2009 *vs* 58% post-2009; p=0·010), illustrating a tendency towards more detailed approaches to estimating non-respiratory influenza burden.

On using the reported burden from the included studies, influenza-associated hospitalisation and mortality estimates were the highest among older adults (appendix p 42). For instance, estimated cardiovascular deaths per population of 100 000 increased markedly with age, with a median of 0·28 (IQR 0·22 to 0·82) in children, 0·10 (0·04 to 0·19) in adolescents, 1·21 (0·66 to 1·45) in adults aged 20 to 44 years, and reaching 57·2 (36·0 to 99·1) in older adults (appendix p 42). A similar age pattern was seen for other non-respiratory causes and for all-cause and combined respiratory and cardiovascular estimates. Within the paediatric population, comparisons of narrower age bands showed that those younger than 1 year generally had a higher burden (appendix p 43); however, data for these narrower age groups were sparse and restricted to fewer causes, so the broad children group was retained for the main analysis (appendix pp 4–5).

When comparing causes, in addition to the high overall burdens from respiratory and cardiovascular and all-cause categories due to their wider coverage of outcomes, cardiovascular-related hospitalisation and mortality rates were generally higher than those for other non-respiratory conditions. Among older adults, the median influenza-associated mortality rates per population of 100 000 were 112·0 (IQR 71·9 to 167·0) for all-cause, 78·7 (59·8 to 110·0) for respiratory and cardiovascular, and 57·2 (36·0 to 99·1) for cardiovascular diseases, as compared with 5·4 (3·6 to 7·7) for cancer and 4·6 (3·3 to 16·5) for diabetes. For comparison, respiratory influenza mortality in this age group was 35·7 (18·0 to 58·2) per population of 100 000 for broad respiratory outcomes and 14·6 (7·8 to 27·1) for pneumonia and influenza (appendix pp 11–13, 42). Within-study ratios of non-respiratory to respiratory estimates exceeded 1 for all-cause and respiratory and cardiovascular across most age groups, and for cardiovascular mortality, particularly among older adults, showing considerable burden beyond what was captured by respiratory coding alone (appendix pp 11–13, 44).

Summaries of hospitalisation and mortality estimates for the most frequently studied causes (ie, all-cause, respiratory and cardiovascular, and cardiovascular) among children and older adults are presented in the appendix (pp 45–48).

Substantial heterogeneity was observed in reported influenza-associated hospitalisation and mortality burden estimates, with *I*^2^ values of 100% for mortality estimates for individuals of all ages, children, and older adults and more than 99% for available hospitalisation estimates for these age groups. Given this extreme heterogeneity, pooled estimates were not calculated. Rate-difference approaches tended to yield higher estimates for all-cause and respiratory and cardiovascular mortality, whereas regression models with an influenza activity proxy usually provided more conservative estimates (appendix p 49). For example, among older adults, the median all-cause mortality rate was 190 (IQR 146–519) per population of 100 000 using the rate-difference approach, as compared with 106 (60·2–146) per population of 100 000 for regression models with an influenza activity proxy and 112 (85·2–191) per population of 100 000 for the Serfling-type approach (appendix p 49).

Random-effects meta-regression investigating drivers of variation suggested that Serfling-type approaches yielded significantly higher mortality estimates (adjusted difference 27·3, 95% CI 1·8 to 52·8; p=0·036), while rate-difference approaches also tended to give higher estimates, although this finding was not significant (p=0·148) after adjustment for influenza period and other study characteristics (figure 3). In a sensitivity model without adjustment for influenza period, mortality estimates derived from rate-difference (44·3, 95% CI 16·0 to 72·6; p=0·002) and Serfling-type (38·9, 14·6 to 63·2; p=0·002) approaches were significantly higher than those from regression models using an influenza proxy, indicating that both the choice of estimation method and key study characteristics, specifically the study period in this case, contribute to variations in reported estimates. Regardless of the method used, estimates of mortality and hospitalisation burden were substantially higher among older adults, with higher mortality rates (80·4 per population of 100 000; 95% CI 57·8 to 103·0) and hospitalisation rates (60·9 per population of 100 000; 37·7 to 84·1) in this age group (figure 3). Mortality estimates appeared lower in the Americas, whereas hospitalisation estimates seemed to be higher in the Western Pacific region and African region, than in the other regions (figure 3).

**Figure 3:**
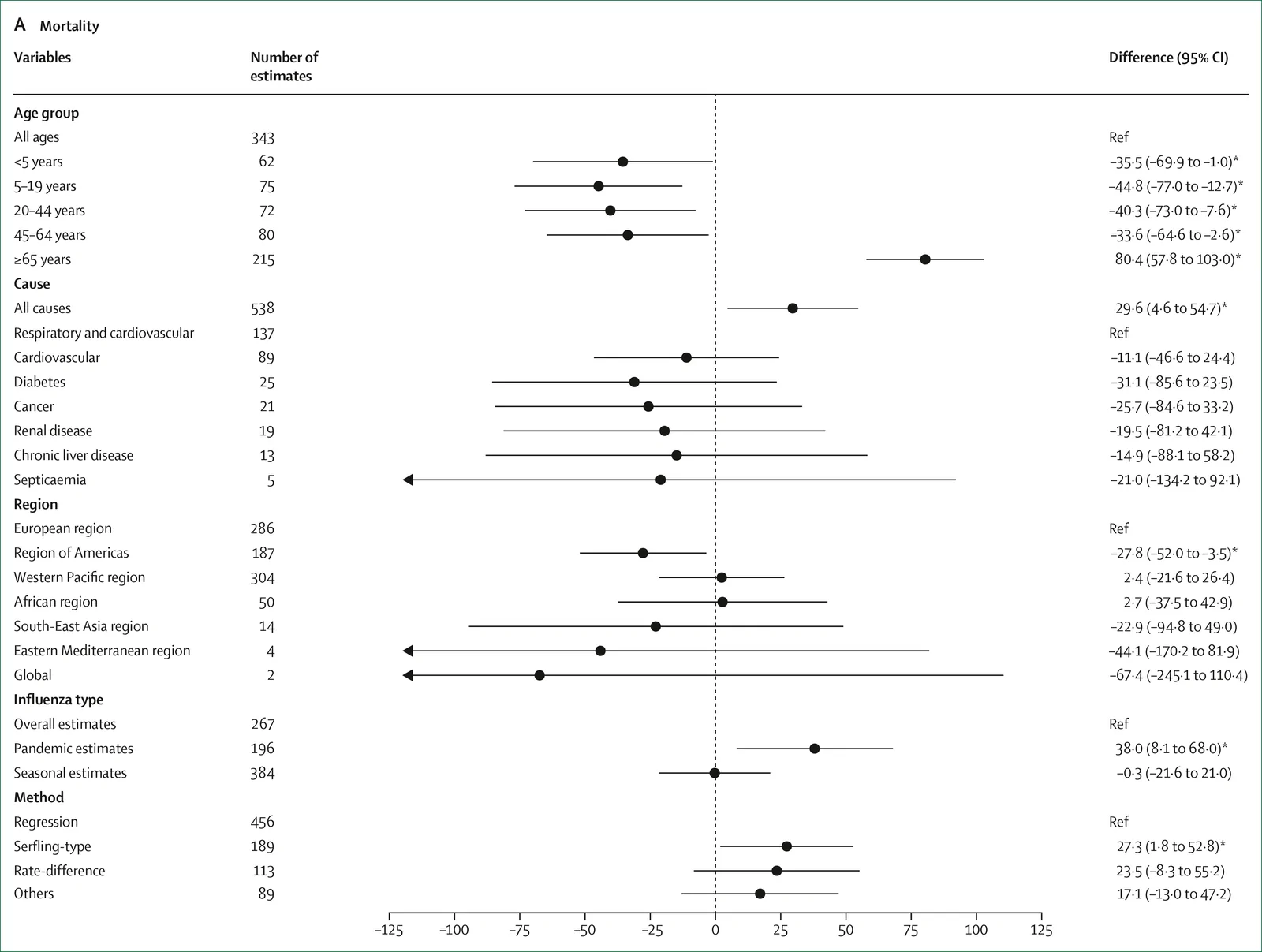

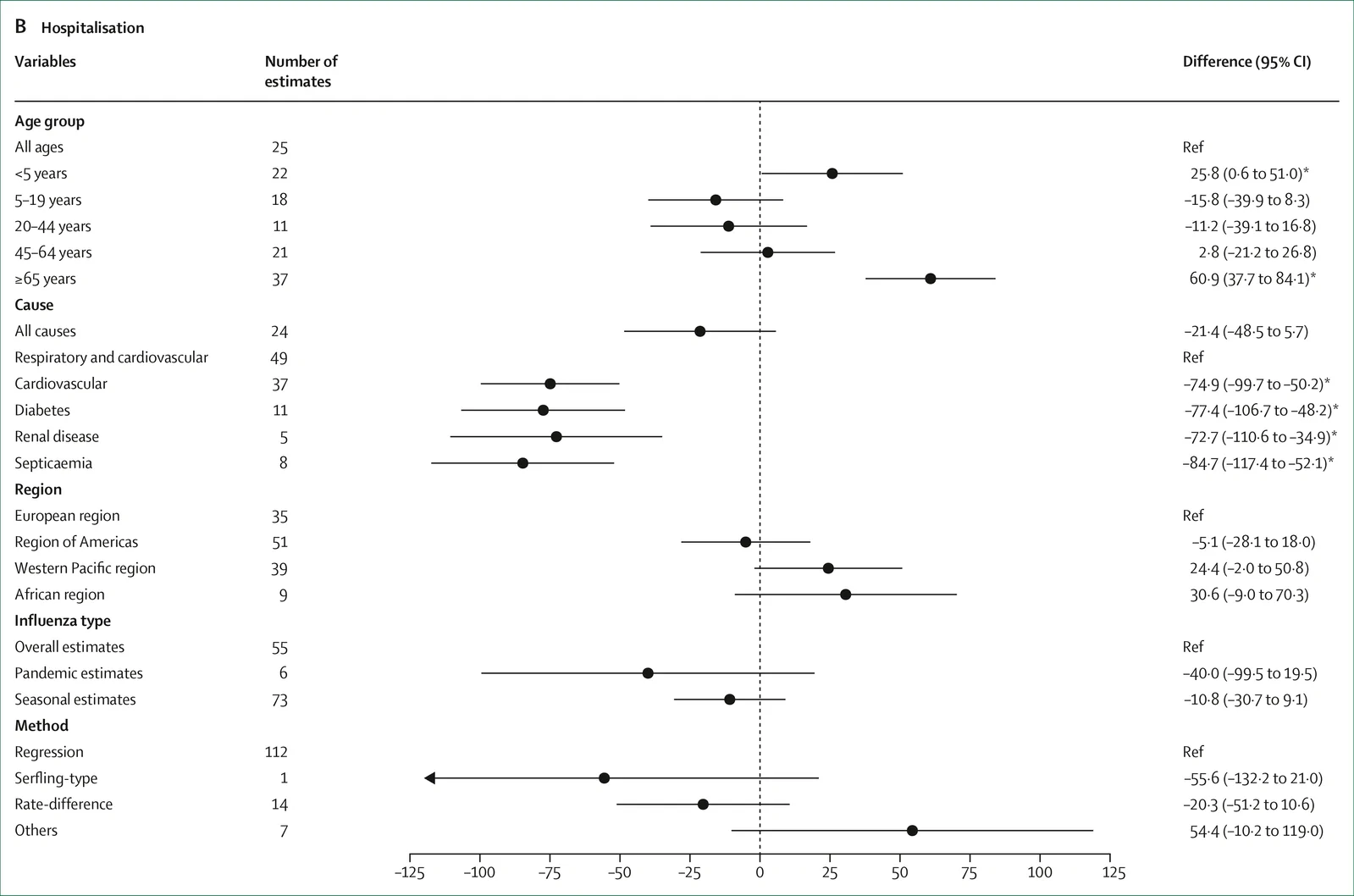
Meta-regression results Estimated differences in (A) influenza-associated mortality and (B) hospitalisation by variables, including age group, cause, region, influenza period reported for each estimate (overall *vs* seasonal *vs* pandemic), and key methodological factors. Points and horizontal lines indicate differences with 95% CIs; values lower than –120 are indicated by arrows at the ends of the lines. Numeric values are shown on the right, with ∗ denoting significant differences (p<0·05).

Sensitivity analysis accounting for multiple estimates within studies yielded broadly consistent results (appendix pp 11, 50). When extending the meta-regression to include respiratory categories as the reference, non-respiratory causes, particularly all-cause, respiratory and cardiovascular, and cardiovascular mortality in older adults, consistently exceeded pneumonia and influenza estimates, reinforcing both the substantial burden beyond respiratory manifestations and consistency of method-related variations across age groups (appendix pp 11–13, 51).

## Discussion

We reviewed 238 published studies on non-respiratory influenza-associated severe disease burden and found substantial variation in burden estimates driven by methodological choices, study period, underlying causes, and age groups, reflecting both real epidemiological variation and heterogeneity in study design and statistical modelling. Our findings highlight that outcomes beyond respiratory disease, particularly in older adults, account for a substantial proportion of the total influenza burden. By systematically synthesising data across a broad range of non-respiratory outcomes, our study provides a more comprehensive picture of the health impact of influenza virus infections beyond respiratory outcomes while highlighting the need for standardised approaches, broader geographical representation, and expanded outcome coverage in future burden assessments.

The substantial non-respiratory influenza burden, particularly among older adults, highlights the need to broaden prevention and control efforts beyond respiratory outcomes.^257^ Vaccination strategies and resource allocation should reflect the full spectrum of influenza burden beyond respiratory aspects,^5^, ^6^ particularly for individuals with cardiovascular disease and other chronic conditions, who could be at elevated risk. Clinicians should also remain alert, especially during influenza seasons, to possible influenza-related cardiovascular, metabolic, and other non-respiratory conditions. Enhanced surveillance systems are also needed to better capture influenza’s full clinical impact and support targeted prevention efforts. These systems could include broadening hospital-based and mortality surveillance for non-respiratory presentations, expanding rapid laboratory testing to strengthen infection identification, strengthening virological surveillance to better define influenza periods, and linking surveillance with administrative health records over longer windows to capture post-acute complications.

Over the past two decades, notable advancements have occurred in burden-estimation methods, reflecting growing recognition of factors influencing baseline and excess burden estimates.^19^, ^258^ However, substantial heterogeneity remains. Rate-difference and Serfling-type approaches generally reported higher excess burden than regression models with influenza activity proxies, possibly because they less effectively distinguish influenza-specific effects from other seasonal drivers or co-circulating pathogens^13^, ^220^ and because study periods might align differently with each method’s assumptions. Regional variation, including lower mortality estimates in the Americas and higher hospitalisation burden in the Western Pacific region, is broadly consistent with literature^10^, ^40^ and could reflect differences in population age structure, comorbidity profiles, health care-seeking behaviour, coding practices, and surveillance intensity. Taken together, these findings highlight the need for greater methodological rigour, transparency, and reporting consistency in future burden-estimation studies, particularly for non-respiratory severe influenza outcomes.

Several important gaps persist. Most studies were from high-income, temperate settings, with little data from the African and Eastern Mediterranean regions, and low-income settings where surveillance capacity, health-care access, and epidemiological patterns could differ substantially,^257^, ^259^, ^260^ thereby restricting the generalisability of these findings to these under-represented regions. Methodological choices can also be influenced by data availability. Regression models using influenza activity proxies require robust viral surveillance data, whereas rate-difference approaches could be more feasible wherever surveillance is low but influenza seasonality is well defined.^120^, ^220^ Although our meta-regression adjusted for both region and estimation method, residual confounding is possible. Despite our broad search strategy, only one study in this Review estimated the population-level burden of longer-term complications or sequelae, highlighting an important gap that future research should address to improve our understanding of the full impact of influenza. Additionally, ecological modelling approaches estimate influenza-associated burden at the population level rather than tracking individual outcomes following infection, thereby restricting causal inference and validation of the full non-respiratory burden.^261^ Addressing these gaps will require investment in surveillance infrastructure and capacity building, particularly in under-represented and low-resource settings, which would also help to disentangle the methodological and epidemiological drivers of heterogeneity in burden estimates.

Although we covered a broad range of non-respiratory outcomes and systematically evaluated methodological factors across a diverse set of studies in this Review, some limitations need to be acknowledged. Our search was restricted to PubMed and Embase, so studies indexed only in regional databases might have been missed. Potential publication bias, and some heterogeneity in outcome definitions, including variations in ICD codes, can be expected. Age groups were inconsistently reported, requiring harmonisation into broad categories. More granular stratification, particularly within the groups of children and older adults, would be informative but was restricted by the heterogeneity of the reported age bands. Standard errors were imputed for many estimates, introducing further uncertainty. Because the meta-regression modelled estimates from studies using different outcome definitions and analytical approaches, residual heterogeneity possibly remains despite adjustment for key covariates. Therefore, future research should prioritise the adoption of standardised definitions and analytical approaches, improved surveillance, and data validation, especially in under-represented regions, and integrate non-respiratory outcomes into routine influenza surveillance. Long-term follow-up studies of laboratory-confirmed influenza cases are also needed to better validate and quantify the full impact of the disease.

To summarise, in our systematic review, we show that influenza imposes a substantial non-respiratory burden, with estimates varying by cause, age group, study period, and methodological approach. This finding emphasises upon the need for standardised approaches in future research. The finding also highlights the importance of recognising and addressing the full spectrum of influenza-associated morbidity and mortality in public health policy and resource allocation and support the need for comprehensive prevention and surveillance strategies that extend beyond respiratory outcomes.

## Declaration of interests

BJC consults for AstraZeneca, Fosun Pharma, GlaxoSmithKline, Moderna, Pfizer, Roche, Haleon, Novavax, Seqirus, and Sanofi Pasteur. All other authors declare no competing interests.
