# Supplementary material for: Estimating the population-level non-respiratory burden of severe influenza: a systematic review of methods and estimates
Source: Lancet Healthy Longev. 2026 Oct;7(10):None. doi: 10.1016/j.lanhl.2026.100934 (PMC13760031; doi:10.1016/j.lanhl.2026.100934)
Supplement: Supplementary file 1 [file mmc1.pdf]

THE LANCET
Healthy Longevity

Supplementary appendix

This appendix formed part of the original submission and has been peer reviewed. We post it as supplied by the authors.

Supplement to: Lin Y, Chen W, Mukherjee S, et al. Estimating the population-level non-respiratory burden of severe influenza: a systematic review of methods and estimates. *Lancet Healthy Longev* 2026. https://doi.org/10.1016/j.lanhl.2026.100934

Supplementary Materials for *Estimating the Population-Level Non-Respiratory Burden of Severe Influenza: A Systematic Review of Methods and Estimates*

Table of Contents

Supplementary Texts* ... *1

Search strategy and study eligibility ... 1

Risk of bias assessment using ROBINS-E ... 3

Definitions ... 4

Summary of studies estimating burden beyond hospitalization and mortality ... 7

Classification of main estimation approaches ... 8

Data synthesis and derivation of overall rate estimates ... 9

Meta-regression model ... 10

Comparisons with the respiratory burden ... 11

Supplementary Table* ... *14

Supplementary Figures* ... *39

Supplementary References* ... *52

Supplementary Texts

Search strategy and study eligibility

Search strategy

We searched for published journal articles using the following combination of search terms (1 and 2 and 3 and 4) in "all fields" in each of the two databases (i.e., MEDLINE (PubMed) and Embase):

1. "mortality" OR "mortalities" OR "death" OR "fatality" OR "burden" OR "hospitalization" OR "hospital" OR "inpatient" OR "in-patient" OR "admitted" OR "admission" OR (("attendance" OR "visit") AND ("medical" OR "emergency" OR "outpatient")) OR "symptomatic" OR "symptom" OR "post-acute" OR "long-term" OR "sequelae" OR "complication"

2. "influenza" OR "flu"

3. "excess" OR "attributable" OR "influenza-associated" OR "influenza-related" OR "influenza-attributed" OR "influenza-attributable" OR "flu-associated" OR "flu-related" OR (("associated with" OR "attributed to" OR "related to" OR "attributable to") AND ("influenza" OR "flu"))

4. ("cardiovascular" OR "cardiac" OR "heart" OR "myocardial" OR "coronary" OR "stroke" OR "ischaemic" OR "ischemic" OR "ischaemia" OR "ischemia" OR "atherosclerosis" OR "atherogenesis" OR "circulatory" OR "cerebrovascular")
OR ("cancer" OR "tumor" OR "neoplasm" OR "neoplasia" OR "malignancy" OR "malignancies")
OR "diabetes"
OR ((("renal" OR "kidney") AND "disease") OR "nephritides" OR "nephrosis" OR "nephrotic")
OR (("liver" OR "hepatic") AND ("disease" OR "dysfunction" OR "cirrhosis" OR "fibrosis"))

OR (((“degenerative” OR “neurologic” OR “nervous system” OR “neurodegenerative”) AND (“disease” OR “disorder” OR “condition”)) OR “Alzheimer” OR “dementia”)
OR (“sepsis” OR “septic” OR “septicaemia” OR “bacteraemia” OR “endotoxemia”)
OR (“cause-specific” OR “nonrespiratory” OR “non-respiratory”)
OR (“all-cause” OR “all cause” OR “all-causes” OR “all causes”)

The above search terms are designed to capture outcomes related to non-respiratory diagnoses including cardiovascular diseases, malignant neoplasms, diabetes, renal diseases, chronic liver diseases, degenerative central nervous system disorders such as Alzheimer disease, and sepsis-related syndromes. These conditions are considered as they represent leading causes of death, discharge diagnoses, or critical illnesses in individuals infected with influenza other than respiratory causes[1-3]. We also include broader search terms like “non-respiratory” or “cause-specific” to capture other possible conditions. In addition, we also included terms related to broader severe outcomes beyond hospitalization and mortality, such as medical attendance (including visits to emergency department and/or outpatients) and post-acute or long-term complications, trying to capture a more complete spectrum of influenza-related disease burden in non-respiratory aspects.

Study eligibility and deduplication

Studies were eligible if they used statistical or mathematical modelling approaches to estimate population-level influenza-associated disease burden for non-respiratory clinical outcomes at the country or regional level. We excluded studies limited to specific subpopulations (e.g., pregnant women), or were reviews, conference abstracts, commentaries, or letters. Because our focus was on population-level burden (i.e., impact) rather than the clinical severity of individual infections (i.e., seriousness of disease) as defined in the WHO pandemic influenza severity assessment (PISA) framework[4], studies reporting only laboratory-confirmed influenza outcomes were excluded, as laboratory confirmation capture only a small proportion of true infections and may underestimate total burden[5], particularly for non-respiratory outcomes where influenza testing is rarely performed.

Publications were considered duplicate reports when they shared the same data source, study population, analytical method, and a substantially overlapping study period. Publications that differed in data source, method, or reported entirely non-overlapping outcomes or causes were treated as distinct studies. When duplicate reports were identified, we retained the most comprehensive publication, defined as the one with longer study period, broader cause and/or outcome coverage, or more detailed age stratification.

Risk of bias assessment using ROBINS-E

Risk of bias was assessed using ROBINS-E, which evaluates seven domains of bias in observational epidemiological studies through structured signalling questions[6]. The tool covers potential bias from confounding, exposure measurement, participant selection, post-exposure interventions, missing data, outcome measurement, and reporting. Each domain is informed by specific signalling questions with standard response options (“Yes”, “Probably yes”, “Probably no”, “No”, or “No information”), which are then translated into a domain-level judgement. For bias due to confounding, we treated age and temporal trend as the minimum critical confounders that should be controlled for (e.g., through age stratification or adjustment, and appropriate modelling of secular and seasonal patterns). Studies that did not account for both age and temporal trends were generally judged at higher risk of confounding bias. For bias in exposure measurement, we rated studies according to whether influenza exposure was measured using well-defined, valid, and reliable methods, such as laboratory-confirmed influenza (PCR or viral culture) or surveillance indicators aligned with WHO influenza surveillance standards. Less specific or poorly described exposure measures were judged to be at higher risk of bias.

After all seven domains were assessed, an overall risk-of-bias judgement was derived following ROBINS-E guidance. Studies were classified as low, some concerns, high, or very high risk of bias, depending on the most severe domain-level judgement and the accumulation of concerns across domains (e.g., one or more serious or critical domains leading to high or very high overall risk).

Figure S2 presents the distribution of risk scores across domains and overall. Most domain-level ratings were low risk of bias, with the notable exception of exposure measurement (domain 2), which showed the highest proportion of non-low ratings, followed by missing data (domain 5) and confounding (domain 1). Overall, 117 studies (49.2%) were rated as low risk, 66 (27.7%) as some concerns, 34 (14.3%) as high risk, and 21 (8.8%) as very high risk. Among the 121 non-low risk studies, exposure measurement was the most frequent source of concern across all categories (some concerns: 74.2%; high risk: 64.7%; very high risk: 100%), followed by missing data (25.8%, 41.2%, 28.6%) and post-exposure interventions (12.1%-20.6%). Studies rated as high or very high risk were additionally distinguished by concerns related to confounding (32.4% and 19.0%, respectively), suggesting that inadequate confounder adjustment was a key differentiator between studies with some concerns and those at higher risk of bias.

Definitions

Matching reported age bands to analytic age groups

We harmonised age categories reported in the included studies to five pre-specified analytic age groups: <5 years, 5-19 years, 20-44 years, 45-64 years, and ≥65 years. Reported age bands were mapped to the analytic age group with which they shared the greatest overlap. Bands falling wholly within a single group were directly assigned; those extending beyond the target range were assigned provided the predominant portion of their span fell within it (e.g., 0-14 years was assigned to the 5-19 group, as 10 of 15 years fell within that range). Bands spanning more than three analytic groups were excluded from age-specific analyses. Among assigned bands, only those covering >50% of the target range were included in meta-analyses, as narrower bands may capture epidemiologically distinct subpopulations.

The reported age bands (in years unless otherwise specified) mapped to each analytic group are listed below, ordered by frequency; those included in meta-analysis were shown in bold. The number of studies with estimates assigned to each group and the subset included in meta-analysis are shown in parentheses (total/meta-analysis):

- <5 years (77 total, 50 in meta-analysis): **0–4**, <1, **1–4**, **<5**, 0–1, 0–0.5, **0–5**, 0–6 months, **0–60 months**, 0.5–1, 0.5–2, **0.5–4**, **1–3**, **1–5**, 12–23 months, **2–4**, 24–35 months, 3–5, 36–60 months, 6–11 months, <2, infants.
- 5–19 years (86 total, 49 in meta-analysis): **5–14**, **5–19**, 5–9, **5–24**, 10–14, **<15**, **1–14**, **<18**, **0–14, 0–19,** 15–19, **0–17, 10–19, 2–17, 5–17, 5–18, <19, 5–15, 5–20, 6–17, <14, <20** and **≤17**.
- 20–44 years (60 total, 43 in meta-analysis): **25–44, 15–44, 18–49**, **20–39, 15–39, 20–44, 20–49**, 40–44, **15–49, 19–49**, 20–24, 25–29, **25–49**, 30–34, 35–39, 15–29, **15–34, 18–34**, 20–29, 20–30, **20–34**, 25–34, **25-39, 25–54**, 30–39, **30–44, 30–50**, 35–44, and 35–49.
- 45–64 years (78 total, 57 in meta-analysis): **45–64, 50–64, 40–64**, 60–64, 50–54, 55–59, 45–49, **50–69**, 55–64, **35–64, 40-59, 40–60**, 45–54, **45–75**, and 50–59.
- ≥65 years (169 total, 132 in meta-analysis): **≥65**, 65–74, **≥75**, >85, **>60**, 65–69, 70–74, 80+, 75–79, 60–69, 75–84, 80–84, 70–79, 65–84, **70+**, 85–89, 65–79, 80–89, 90+, 90–94, 60-74, 60–79, 65–75.

Other reported age bands (e.g., <65, 15–64, 0–64, 18–64, 50+, 18+, 5+) were not assigned to any analytic age group because they substantially spanned two or more groups without predominantly falling within any single one (e.g., 18-64 spanning three groups), or their boundaries split approximately equally between two adjacent groups (e.g., 40-49, split evenly between the 20-44 and 45-64 groups).

Among the 77 studies including <5 years group, 53 reported the broad <5 age band, while only 26 and 19 reported narrower bands for <1 and 1-4 years, respectively. Comparisons of these narrower bands showed notably higher burden in <1 year compared to the 1-4 and broad <5 group, e.g., for all-cause mortality (Fig S5). However, beyond all-cause, R+C and cardiovascular outcomes, data were available only for the broad <5 group, suggesting the limited data for finer pediatric age stratification. We therefore retained the broad <5 years group as the primary pediatric category in the main analysis to maximize statistical power.

Classification of study countries by WHO region and World Bank income level

We classified each study country according to the WHO regional groupings and World Bank income levels, using the country (or subregion) reported in the original article. These classifications were used to summarise the distribution of studies by region and economic context and to explore possible regional and income-related differences in influenza-associated severe disease burden.

By WHO region[7], countries represented in the included studies were:

- African Region (AFR): South Africa (8 studies), Kenya.
- Region of the Americas (AMR): United States (54), Canada (9), Mexico (7), Argentina (5), Brazil (3), Chile (3), Uruguay (2), Colombia, Paraguay, Peru, Venezuela.
- Eastern Mediterranean Region (EMR): Iran.
- European Region (EUR): United Kingdom (25), Spain (14), Italy (12), France (11), Denmark (9), Germany (9), Netherlands (9), Portugal (8), Switzerland (8), Norway (7), Sweden (6), Europe (unspecified; 5), Finland (4), Belgium (3), Czech Republic (3), Israel (3), Austria (2), Poland (2), Bulgaria, Greece, Hungary, Ireland, Malta, Romania, Slovenia.
- South-East Asia Region (SEAR): India (3), Thailand (2), Sri Lanka.
- Western Pacific Region (WPR): Hong Kong (21), mainland China (19), Japan (15), Australia (12), Singapore (7), New Zealand (4), South Korea (4), Philippines (3), Taiwan (2).

Numbers in parentheses indicate the number of included studies that reported estimates for that country; where no number is given, only one included study reported estimates for that country.

By World Bank income level[8], study regions were categorised as follows:

- High-income: United States, United Kingdom, Hong Kong, Japan, Spain, Italy, Australia, France, Canada, Denmark, Germany, Netherlands, Portugal, Switzerland, Norway, Singapore, Sweden, Finland, New Zealand, South Korea, Belgium, Chile,

Czech Republic, Israel, Austria, Poland, Taiwan, Uruguay, Bulgaria, Greece, Hungary, Ireland, Malta, Romania, Slovenia.

- Upper-middle-income: mainland China, South Africa, Mexico, Argentina, Brazil, Thailand, Colombia, Iran, Paraguay, Peru.
- Lower-middle-income: India, Philippines, Kenya, Sri Lanka.

Summary of studies estimating burden beyond hospitalization and mortality

A total of 18 studies assessed outcomes beyond hospitalization and mortality, most of which focused on all-cause, R+C, and cardiovascular outcomes. These outcomes fell broadly into three categories. The first related to severe disease indicators, including ICU admission, hospitalization with mechanical ventilator use (N=3), out-of-hospital cardiac arrest (N=1) and influenza-associated complications (N=1). The second encompassed healthcare utilization, including emergency department visits (N=3), outpatient visits or GP consultations (N=3), medically attended illness (N=2), and antibiotic prescriptions (N=1). The third captured societal or population-level burden, including years of life lost (N=3), sickness insurance or benefit claims (N=2), and absenteeism (N=1). Note that some studies reported multiple outcome types, so counts sum to more than 18.

Data sources and settings for these outcomes varied by category. Studies assessing severe disease indicators primarily used hospital-based or population-based active surveillance systems or hospital discharge databases, drawing on data from tertiary care or referral hospital networks. Studies of healthcare utilization relied on emergency department administrative databases, sentinel general practice surveillance networks, or health insurance claims databases, capturing care delivered in outpatient, primary care, or emergency department settings. Studies estimating years of life lost used vital registration data at the population level, while those reporting sickness benefit claims used national social insurance records.

Among these studies, all-cause outcomes spanned the full range of severity categories, while R+C and cardiovascular outcomes were primarily captured through outpatient,

emergency department, or medically-attended illness. Non-respiratory, non-circulatory outcomes were also reported for medically-attended or severe illness (Table S1).

Classification of main estimation approaches

Influenza-associated burden is typically estimated either by statistically linking population-level health outcomes to influenza activity proxies, or by quantifying excess rates above an expected baseline during periods of influenza circulation, with the choice of approach often depending on data availability and surveillance infrastructure. To facilitate summary and comparison, we classified the main statistical approaches used to derive influenza-associated mortality and hospitalization rates into three major categories[9], based on how each study linked observed outcomes to influenza activity and/or defined excess burden.

The first category refers to regression models with an influenza activity proxy, which included regression models that explicitly incorporated an influenza activity indicator (e.g. laboratory-confirmed positivity, virologic surveillance measures, or influenza-like illness rates) as a covariate. These models typically related weekly or monthly mortality or hospitalization counts to influenza activity while adjusting for secular trends, seasonality, and other covariates (such as weather or co-circulating pathogens), and then quantified influenza-attributable rates from the fitted influenza terms.

The second category is regression models without an influenza activity proxy, which did not use an explicit influenza activity measure. Instead, influenza-associated burden was estimated from excess rates above a modelled baseline, often defined using seasonal patterns and long-term trends (e.g. Serfling-type). Excess rates during influenza seasons or epidemic periods were interpreted as the influenza-associated component.

Lastly, rate-difference methods estimated influenza-associated burden by directly subtracting observed outcome rates (e.g., all-cause or cause-specific mortality or hospitalization rates per 100,000 population) during reference (non-epidemic) periods from those during predefined epidemic (or influenza season) periods, without fitting a

regression model. The excess rate was calculated as the arithmetic difference between the observed rate during high-activity periods and the rate during baseline periods.

In addition, 28 papers used other main methods not captured by these three categories. These included autoregressive integrated moving average (ARIMA) models, relative mortality distribution models, multiplier models, survival regression, and machine-learning approaches (e.g. XGBoost). A small number of studies used extrapolation approaches (e.g. generalized additive models or linear regression combined with extrapolation) to extend estimates beyond the directly observed populations.

Data synthesis and derivation of overall rate estimates

For studies that did not report an overall rate estimate for the study period (e.g., only sub-annual, type-specific, or count data), we derived study-level summary rates as follows.

For multi-year studies, we first obtained annual rates and then calculated a single study-level estimate by averaging these annual values over the full study period. When estimates were reported only for sub-annual periods (e.g., by month, season, or epidemic wave), we summed the period-specific values within each calendar year to derive an annual rate, and then averaged these annual rates across years. For studies reporting only type-specific excess estimates within a year (e.g. A(H1N1), A(H3N2), B), we summed the type-specific values to obtain an all-influenza annual estimate before averaging across years.

Because seasonal and pandemic periods may differ in underlying influenza activity and burden[10], we also standardised how these were handled when deriving overall estimates for comparison. If a study reported only one overall estimate (without separating seasonal and pandemic periods), influenza type categories followed the definitions in the original paper. For studies that reported both seasonal and pandemic estimates over overlapping calendar years, we summed all type-specific estimates within each year to derive an all-influenza annual value, and then calculated a single summary estimate for the entire

period. When seasonal and pandemic estimates were reported for non-overlapping periods, we retained them as separate records and classified them as seasonal-only or pandemic-only estimates, rather than combining them into a mixed overall estimate. Hence, in the meta-regression models this influenza type variable reflects only the predominant influenza types represented in each included estimate. We focused on overall estimates to maximise comparability across heterogeneous studies and therefore did not additionally extract or compare seasonal and pandemic estimates when an overall estimate was available.

For studies reporting only age-band-specific rates within older adults (e.g. 65-74, 75-79, ≥80 years), and no overall ≥65-year rate, we derived an approximate ≥65-year estimate by taking the simple arithmetic mean of the reported age-band rates. This assumes each age band contributes similarly to the ≥65 population, so these derived estimates should be interpreted with caution and used only for approximate comparison. For studies that reported only the number of burden estimate, we calculated rates using population denominators obtained from external sources (e.g., national or subnational census data, or population statistics from local government or statistical bureaus).

Meta-regression model

We used random-effects meta-regression to identify factors associated with variation in reported influenza-associated mortality and hospitalization rates. Separate models were fitted for mortality and hospitalization, with between-study heterogeneity (τ^2) estimated using restricted maximum likelihood. Covariates included age group, study region, cause of death or hospitalisation, influenza type (seasonal, pandemic, or overall), and modelling approach, as described in the main **Methods**.

As only around 35.3% of available estimates were reported with a standard error (SE), we imputed SEs using a regression model[9] for estimates without an SE:

$$\log(SE_i) = |MR_i| + age_i + cause_i$$

where SE_i is the SE of the ith estimate, $|MR_i|$ is the absolute value of the influenza-associated mortality or hospitalization rate, age_i is the age group, and $cause_i$ the cause category for that estimate. Separate imputation models were fitted for mortality and hospitalization outcomes, with R^2 of 0.65 and 0.51 respectively, indicating goodness of fit.

For geographic classification, we compared models using WHO region versus World Bank income level, restricting to estimates with information available for both classifications. For mortality, the Akaike Information Criterion (AIC) was 10,704 for the model with WHO regions and 10,738 for the model with income levels. For hospitalization, the corresponding AIC values were 1,382 and 1,401. As models using WHO regions consistently had lower AIC values, we used WHO region as the geographic classification in the final meta-regression models.

As individual studies could contribute multiple estimates (e.g., for different age groups, causes, or time periods), we conducted a sensitivity analysis applying cluster-robust variance estimation (sandwich estimator) at the study level to evaluate the stability of our meta-regression results. Results from the cluster-robust approach were broadly consistent with those from the primary random-effects meta-regression models for both mortality and hospitalization outcomes (Fig S12), supporting the robustness of the primary findings.

Comparisons with the respiratory burden

To improve interpretation of the non-respiratory burden, we also extracted respiratory burden estimates from studies that reported these outcomes alongside non-respiratory estimates. Overall, 144/238 (60.5%) of included studies reported at least one respiratory outcome, including broad respiratory categories, pneumonia and influenza (P&I), chronic obstructive pulmonary disease (COPD), pneumonia, tuberculosis, bronchitis and related conditions, with most providing P&I and/or broad respiratory estimates. This included 29/41 (70.7%) hospitalization studies and 125/213 (58.7%) mortality studies.

Because P&I and broad respiratory were the most frequently reported categories, we focused on respiratory hospitalization or mortality rate estimates for these two groups when available. A total of 110 studies contributed such estimates, including 99 with mortality outcomes and 18 with hospitalization outcomes. Data extraction and synthesis followed the same procedures as for the non-respiratory burden estimates.

In addition to comparing absolute respiratory and non-respiratory rates (Fig S4), we calculated within-study ratios of non-respiratory to respiratory burden for each available stratum (e.g., same age group, outcome type, and cause category). For each study and stratum, the non-respiratory rate was divided by the corresponding respiratory rate from the same study. This within-study ratio reduces the influence of between-study differences in design, data sources, and background incidence, providing a clearer measure of how large the non-respiratory burden is relative to respiratory outcomes. For cause-specific categories such as cardiovascular disease, the ratio directly quantifies the relative magnitude of non-respiratory burden compared to respiratory burden, while for broader categories such as all-cause and R+C that encompass respiratory causes, ratios exceeding one indicate the extent to which influenza-associated burden extends beyond what is captured by respiratory coding alone, reflecting additional contributions from cardiovascular and other non-respiratory pathways (Fig S6).

To further assess these relationships while adjusting for other study characteristics, we extended the main meta-regression model to include P&I and respiratory-only estimates as explicit reference categories. Among adults aged ⩾65 years, this analysis showed the expected pattern, as coefficients for cardiovascular, R+C and all-cause outcomes were clearly higher than for P&I and respiratory-only estimates, confirming that non-respiratory, particularly cardiovascular, outcomes contribute substantially to the overall influenza-associated burden in older adults (Fig S13). In younger age groups, however, coefficients for R+C and all-cause outcomes were similar to, or slightly lower than, those for P&I and respiratory-only outcomes, likely suggesting that influenza in these ages being dominated by respiratory manifestations, with relatively few cardiovascular complications

and greater background noise in broad all-cause measures[11,12]. Despite these age-specific differences in cause patterns, the relative differences between estimation methods observed in the main analyses were broadly similar across age groups, indicating that method-related variation in burden estimates is not confined to specific age stratum.

Supplementary Table

Table S1. Basic information on included studies (N=238).

Study id	Country/ region	Study period	Whether the estimate was for seasonal or pandemic periods, or both	Statistical or modelling technique	Influenza activity proxy	Cause of outcome	Whether the study reported rate or number of burden estimate	Reported age bands (unit in years unless otherwise specified)
Alling, 1981[13]	US	1968-1976	Seasonal	Regression model with flu proxy (Linear)	Number of acute respiratory deaths	Mortality: cardiovascular, all-cause	number	65+, all ages
Andreasen, 2011[14]	Copenhagen, Denmark, Norway, Sweden, England, Finland, France, Italy, Netherlands, Spain, Sweden, US	1915-1923	1918 pandemic	Rate-difference; Regression model without flu proxy (Linear)	N	Mortality: all-cause	rate	<1, 1-14, 15-44, 45+, all ages
Ang, 2017[15]	Singapore	2010-2014	Seasonal	Regression model with flu proxy (Negative binomial)	Proportion of specimens testing positive for influenza	Hospitalization: cardiovascular, ischemic heart disease, congestive heart failure	both rate and number	<50, 50-64, 65-79, 80+, all ages
Ansart, 2009[16]	Finland, England and Wales, Scotland, Denmark, Norway, France, Switzerland, Germany, Sweden, Netherlands, Spain, Portugal, Bulgaria, Italy	1906-1922	1918 pandemic	Regression model without flu proxy (Linear)	N	Mortality: all-cause	both rate and number	all ages
Aungkulanon, 2015[17]	Thailand	2006-2011	Seasonal and 2009 pandemic	Regression model with flu proxy (Negative binomial)	Proportion of specimens testing positive for influenza	Mortality: cardiovascular, all-cause	both rate and number	<65, 65+, all ages
Azziz-Baumgartner, 2013[18]	Argentina	2002-2009	Seasonal and 2009 pandemic	Regression model without flu proxy (Linear)	N	Mortality: R+C; Hospitalization: R+C	both rate and number	all ages

Bertolani, 2018[19]	Italy	2008-2015	Seasonal and 2009 pandemic	Regression model with flu proxy (Negative binomial)	Incidence of influenza syndrome	Hospitalization: R+C	number	all ages
Blackburn, 2018[20]	England	2004-2015	Seasonal and 2009 pandemic	Regression model with flu proxy (Poisson)	Counts of lab-confirmed influenza cases	Hospitalization: myocardial infarction, stroke	number	45-64, 65-74, 75+
Bonmarin, 2015[21]	France	2000-2009	Seasonal	Rate-difference	N	Mortality: all-cause	number	65+
Brinkhof, 2006[22]	Switzerland	1969-1999	Seasonal	Regression model with flu proxy (Poisson)	Influenza or P&I mortality rates	Mortality: all-cause	both rate and number	60-69, 70-79, 80+, 60+
CaldasAfonso, 2024[23]	Portugal	2008-2018	Seasonal	Regression model with flu proxy (Poisson)	Weekly ILI incidence rate	Mortality: R+C, all-cause; Hospitalization: R&C, all-cause	both rate and number	<5
Carrat, 1995[24]	France	1980-1990	Seasonal	ARIMA	Influenza or P&I mortality rates	Mortality: R+C, diabetes, renal diseases, lung cancer, breast cancer, cirrhosis, and other unknown or unspecified causes	rate	75+
Chan, 2010[25]	Taiwan	1999-2007	Seasonal	Regression model with flu proxy (Negative binomial)	Influenza virus isolation rate	Mortality: R+C, all-cause	rate	65+
Chandra, 2021[26]	US (Michigan)	1912-1920	1918 pandemic	ARIMA	N	Mortality: all-cause	proportion	<1, 1-5, 5-64, 65+
Charu, 2011[10]	Mexico	2000-2010	Seasonal and 2009 pandemic	Regression model without flu proxy (Linear)	N	Mortality: R+C, all-cause	both rate and number	<5, 5-19, 20-59, 60+, all ages
Charu, 2013[27]	US	2003-2010	Seasonal and 2009 pandemic	Regression model with flu proxy (Poisson)	Proportion of specimens testing positive for influenza	Mortality: R+C	both rate and number	<5, 5–24, 25–44, 45–64, 65+, all ages
Chaves, 2023[28]	Global	2010-2019	Seasonal	GAM for individual countries and meta-regression for global and regional estimates	Proportion of specimens testing positive for influenza	Mortality: IHD	both rate and number	50+, 50-64, 65-74, 75+
Cheng, 2015[29]	Argentina, Chile, Mexico, southern Brazil,	2002-2008	Seasonal	Regression model without flu proxy	N	Mortality: cardiovascular, R+C	both rate and number	0-64, 65–74, 75+, all ages

	Paraguay, US, Uruguay			(Linear) + extrapolation				
Cheng, 2020[30]	Philippines	2006-2015	Seasonal and 2009 pandemic	Regression model with flu proxy (Negative binomial)	Proportion of specimens testing positive for influenza	Mortality: all-cause	both rate and number	0-4, 5-9, 10-19, 20-59, 60+, all ages
Choi, 1982[31]	US	1967-1978	Seasonal	ARIMA; Regression model without flu proxy (Linear); Regression model with flu proxy (Linear)	N; N; RGCP reported influenza rates	Mortality: all-cause	rate	all ages
Chow, 2006[32]	Singapore	1996-2003	Seasonal	Regression model with flu proxy (Negative binomial)	Proportion of specimens testing positive for influenza	Mortality: R+C, all-cause	both rate and number	20-64, 65+, all ages
Chowell, 2011[33]	Peru	1918-1920	1918 pandemic	Regression model without flu proxy (Linear)	N	Mortality: all-cause	both rate and number	all ages
Chowell, 2014[34]	Spain	1915-1919	1918 pandemic	Regression model without flu proxy (Linear)	N	Mortality: all-cause	both rate and number	all ages
Cilek, 2018[35]	Madrid (Spain)	1918-1920	1918 pandemic	Regression model without flu proxy (Linear)	N	Mortality: all-cause	rate	<5, 5-14, 15-24, 25-49, 50-69, 70+
Clay, 2019[36]	US	1915-1925	1918 pandemic	Regression model without flu proxy (REM)	N	Mortality: all-cause	both rate and number	all ages
Cobos, 2016[37]	US (Maricopa County, Arizona)	1954-1961	1957 pandemic	Regression model without flu proxy (Linear)	N	Mortality: all-cause	rate	<5, 5-14, 15-24, 25-44, 45-64, 65+, all ages
Cohen, 2010[38]	South Africa; US	1998-2005	Seasonal	Regression model without flu proxy (Linear)	N	Mortality: cardiovascular, cancer, diabetes, all-cause, IHD, cerebrovascular disease	both rate and number	65-69, 70-74, 75-79, 80+, 65+
Cohen, 2012[39]	South Africa, US	1987-2005	Seasonal	Regression model without flu proxy (Spline)	N	Mortality: all-cause	rate	25-54, 65+
Cohen, 2018[40]	South Africa	2009-2013	Seasonal and 2009 pandemic	Regression model with flu proxy (GAM)	Incidence of confirmed infection	Mortality: cardiovascular, all-cause	both rate and number	<1, 1-4, 5-19, 20–44, 45–64, 65–74, 75+, all ages, <5, 5+
Collins, 1953[41]	US	1918-1951	Seasonal and 1918 pandemic	Rate-difference	N	Mortality: all-cause, other causes (beyond P&I)	rate	all ages

Comas-Garcia, 2011[42]	Mexico (San Luis Potosı´)	2003-2009	Seasonal and 2009 pandemic	Rate-difference	N	Mortality: cardiovascular, R+C, all-cause	both rate and number	0-4, 5-19, 20-59, 60+, all ages
Cooper, 2015[43]	Thailand	2005-2009	Seasonal	Regression model with flu proxy (GAM)	Product of the proportion of lab tests positive for influenza and the number of ILI patients	Mortality: cardiovascular, cancer, diabetes, renal diseases, chronic liver diseases, septicaemia, all-cause	rate	<18, 18-59, 60+, all ages
Cox, 2010[44]	Belgium	2006-2007	Seasonal	Regression model without flu proxy (Poisson)	N	Mortality: all-cause	number	85+, all ages
Czaja, 2020[45]	US (Colorado)	2007-2016	Seasonal and 2009 pandemic	Regression model with flu proxy (Negative binomial)	Proportion of specimens testing positive for influenza	Mortality: R+C; Hospitalization: R+C	both rate and number	0-4, 5-49, 50-64, 65+, all ages
Dahal, 2018[46]	US (Arizona)	1918-1921	1918 pandemic	Regression model without flu proxy (Linear)	N	Mortality: all-cause	rate	<5, 5-14, 15-24, 25-44, 45-64, 65+
Dawood, 2012[47]	Global	2009-2010	2009 pandemic	Multiplier	N	Mortality: cardiovascular, R+C; Years of life lost	both rate and number	18-64, 65+, all ages
Deng, 2025[48]	Mainland China	2012-2021	Seasonal	Regression model with flu proxy (GAM)	Proportion of specimens testing positive for influenza	Mortality: all-cause	rate	all ages
Dushoff, 2006[49]	US	1979-2001	Seasonal	Regression model with flu proxy (Linear)	Proportion of specimens testing positive for influenza	Mortality: R+C, all-cause	number	all ages
Egger, 1989[50]	Switzerland	1969-1985	Seasonal	Regression model without flu proxy, ARIMA	N	Mortality: all-cause	both rate and number	1-59, 60-69, 70-79, 80-89
Eigkhoff, 1961[51]	US	1957-1960	Seasonal	Regression model without flu proxy (Linear)	N	Mortality: all-cause, cardiovascular-renal, all other causes	number	<1, 1-14, 15-24, 25-44, 45-64, 65+, all ages
Emukule, 2017[52]	Western Kenya	2007-2013	Seasonal	Regression model with flu proxy (Negative binomial); rate-difference	Proportion of specimens testing positive for influenza; N	Mortality: all-cause	both rate and number	<5, 5-49, 50+, all ages
Fattore, 2024[53]	Italy	2008-2019	Seasonal	Regression model with flu proxy	ILI outpatient consultation rates	Hospitalization: cardiovascular, R+C, all-cause	both rate and number	0-4, 5-14, 15-64, 65+, all ages

				(Negative binomial)				
Faust, 2020[54]	US (New York City)	1914-1918	1918 pandemic	Rate-difference	N	Mortality: all-cause	rate	all ages
Feng, 2012[55]	Mainland China (Dalian, Qingdao, Zhaoyuan, Shanghai, Wuhan, Yichang, Ningbo, Guangzhou)	2003-2008	Seasonal	Regression model with flu proxy (Negative binomial)	Proportion of specimens testing positive for influenza	Mortality: R+C, all-cause, IHD	both rate and number	0-64, 65+, all ages
Fireman, 2009[56]	US (California)	1996-2005	Seasonal	Regression model without flu proxy (Poisson)	N	Mortality: all-cause	number	65+
Fleming, 1996[57]	England and Wales	1986-1995	Seasonal	Rate-difference	N	Mortality: all-cause; GP consultation: myocardial infarction, cerebrovascular disease	both rate and number	all ages
Fleming, 2000[58]	England and Wales	1988-1997	Seasonal	Rate-difference	N	Mortality: all-cause Hospitalization: cardiovascular	number	65-74, 75+, all ages
Fleming, 2005[59]	England	1989-2000	Seasonal	Rate-difference	N	Mortality: all-cause	both rate and number	<1, 1-4, 5-9, 10-14, 15+, all ages
Fleming, 2005[60]	England	1994-2000	Seasonal	Rate-difference	N	Mortality: R+C, cardiovascular, IHD; Hospitalization: R+C, cardiovascular; GP practice episode: R+C, cardiovascular	both rate and number	46-64, 65-74, 75+, 45+
Foppa, 2008[61]	US	1995-2005	Seasonal	Regression model with flu proxy (Poisson)	Influenza or P&I mortality rates	Mortality: all-cause	number	<18, 18–49, 50–64, 65+, all ages
Foppa, 2015[62]	US	2005-2014	Seasonal and 2009 pandemic	Regression model with flu proxy (Negative binomial)	Proportion of physician visits for ILI multiplied by the proportion of specimens testing positive for influenza	Mortality: R+C	number	0.5-4, 5-19, 20-64, 65+, all ages

Freitas, 2013[63]	Southern Brazil	1980-2008	Seasonal	Regression model without flu proxy (Linear)	N	Mortality: R+C	both rate and number	60+, all ages
Freitas, 2018[64]	Southern Brazil	2002-2015	Seasonal and 2009 pandemic	Regression model without flu proxy (Linear)	N	Mortality: all-cause	both rate and number	24-59, 60-69, 70-79, 80+
Froes, 2022[65]	Portugal	2008-2018	Seasonal	Regression model with flu proxy (Poisson)	ILI outpatient consultation rates	Mortality: R+C, all-cause; Hospitalization: R+C, all-cause	both rate and number	0-4, 5-18, 19-49, 50-64, 65-74, 75+, 65+, all ages
Gavrilova, 2020[66]	Italy, US	1917-1921	1918 pandemic	Rate-difference	N	Mortality: all-cause	rate	all ages
Glatman-Freedman, 2017[67]	Israel	2005-2013	Seasonal	Rate-difference	N	Hospitalization: cardiovascular, R+C, all-cause	rate	<1, 1-4, 5-14, 15-24, 25-34, 35-44, 45-54, 55-64, 65-74, 75-84, 85+, all ages
Goldstein, 2012[1]	US	1997-2007	Seasonal	Regression model with flu proxy (Linear)	Proportion of physician visits for ILI multiplied by the proportion of specimens testing positive for influenza	Mortality: cardiovascular, cancer, diabetes, renal diseases, septicaemia, all-cause, heart disease, myocardial infarction, IHD, cerebrovascular disease, Alzheimer's, unintentional injuries	rate	all ages
Goldstein, 2015[68]	US (New York)	2003-2011	Seasonal and 2009 pandemic	Regression model with flu proxy (Linear)	Percentage of ILI ED visits multiplied by the percent of positive respiratory specimens	Hospitalization: cardiovascular, diabetes, renal diseases, degenerative diseases of the nervous system, septicaemia	rate	<1, 1-4, 5-17, 18-49, 50-64, 65-74, 75+
Goldstein, 2023[69]	France	2014-2023	Both	Regression model with flu proxy (GLM)	Proportion of physician visits for ILI multiplied by the proportion of specimens testing positive for influenza	Mortality: all-cause	both rate and number	all ages
Gómez-Gómez, 2021[70]	Mexico	1998-2015	Both	Regression model without flu proxy (Linear)	N	Mortality: diabetes	both rate and number	20-59, 60+, all ages

Gran, 2010[71]	Norway	1975-2004	Seasonal	Regression model with flu proxy (Poisson)	Number of reported ILI cases	Mortality: all-cause	both rate and number	0-4, 5-14, 15-64, 65+, all ages
Gran, 2013[72]	Norway	1998-2011	Seasonal and 2009 pandemic	Regression model with flu proxy (Poisson); Regression model without flu proxy (Poisson)	Reported cases of ILI; N	Mortality: all-cause	both rate and number	0-4, 5-14, 15-64, 65+, all ages
Green, 2013[73]	England and Wales	2006-2012	Seasonal and 2009 pandemic	Regression model without flu proxy (Poisson for <15 and 15-44, negative binomial for 45-64 and 65+)	Proportion of physician visits for ILI multiplied by the proportion of specimens testing positive for influenza	Mortality: R+C, all-cause	both rate and number	<15, 15-44, 45-64, 65+, all ages
Green, 2015[74]	England	2012-2013	Seasonal	Regression model without flu proxy (Poisson)	Proportion of specimens testing positive for influenza	Mortality: all-cause	both rate and number	all ages
Hai, 2025[75]	Hong Kong	2014-2020	Seasonal	Regression model with flu proxy (Negative binomial)	Proportion of ILI consultations	Out-of-hospital cardiac arrest: cardiovascular, non-cardiovascular and non-respiratory, all cause	rate	<65,65-84, 85+
Hanzlíková, 2026[76]	Czech Republic	1982-2020	Both	Regression model with flu proxy (GAM)	ARI morbidity collected by GP	Mortality: all-cause	number	all ages
Hao, 2020[77]	Mainland China (Hefei)	2012-2017	Seasonal	Regression model with flu proxy (Poisson)	Proportion of physician visits for ILI multiplied by the proportion of specimens testing positive for influenza	Mortality: all-cause; Year of life lost	rate	60-64, 65-69, 70-74, 75-79, 80-84, 85-89, 60+
Hardelid, 2011[78]	England and Wales	1999-2010	2009 pandemic	Regression model without flu proxy (Poisson)	N	Mortality: all-cause	number	<1, 1–4, 5–14, 15–24, 25–44, 45–64, 65+, 65–74, 75–84, 85+
Hardelid, 2013[79]	England and Wales	1999-2010	Seasonal and 2009 pandemic	Regression model with flu proxy (GLM)	Positive flu count	Mortality: all-cause	number	<15, 15-44, 45-75, 75+, all ages
Heudorf, 2026[80]	Germany	2020-2023	Seasonal	Rate-difference	N	Mortality: all-cause	number	0-29, 30-59, 60-69, 70-79, 80+, all ages

Hong, 2019[81]	South Korea	2009-2016	Seasonal and 2009 pandemic	Regression model with flu proxy (Negative binomial)	Proportion of specimens testing positive for influenza	Mortality: R+C, all-cause	both rate and number	<65, 65+, all ages
Honkanen, 2006[82]	Northern Finland	1992-1994	Seasonal	Rate-difference	N	Hospitalization: cardiovascular	rate	65+
Housworth, 1974[83]	US	1957-1966	Seasonal and 1957 pandemic	Regression model without flu proxy (Linear)	N	Mortality: cancer, diabetes, all-cause, chronic rheumatic heart disease, arteriosclerotic heart disease, heart/circulatory/ nervous, all other	both rate and number	infants, all ages
Hrehorovich, 1972[84]	US (Pennsylvania)	1968-1969	Seasonal	Regression model without flu proxy (Linear)	N	Mortality: diabetes, all-cause, heart disease, cerebrovascular disease	both rate and number	<1, 1-4, 5-9, 10-19, 20-39, 40-59, 60-74, 75+, all ages
Huy, 2012[85]	Germany	2001-2006	Seasonal	Rate-difference	N	Mortality: cardiovascular, all-cause, heart disease, myocardial infarction, IHD, cerebrovascular disease, heart failure	both rate and number	all ages
Imaz, 2006[86]	Argentina	1992-2002	Seasonal	ARIMA	N	Mortality: all-cause	both rate and number	65+
Jang, 2023[87]	South Korea	2013-2017	Seasonal	Others: Difference in mortality between individuals with/without influenza	N	Mortality: cardiovascular, cancer, renal diseases, all-cause, myocardial infarction; hypertension, ischemic stroke, hemorrhagic stroke, neurologic	rate	<19, 20-39, 40-64, 65+
Jansen, 2007[88]	Netherlands	1997-2003	Seasonal	Rate-difference	N	Mortality: all-cause	both rate and number	0-1, 2-17, 18-49, 50-64, 65+
Jin, 2020[89]	Mainland China (Shanghai)	2010-2015	Seasonal	Regression model with flu proxy (Poisson)	Proportion of physician visits for ILI multiplied by the proportion of specimens testing	Mortality: R+C, all-cause, IHD	rate	<65, 65+, all ages

					positive for influenza			
Jin, 2022[90]	Mainland China (Shanghai)	2010-2019	Seasonal	Regression model with flu proxy (Poisson)	Proportion of physician visits for ILI multiplied by the proportion of specimens testing positive for influenza	Mortality: cardiovascular, IHD, stroke	rate	60-64, 65-69, 70-74, 75-79, 80-84, 85+
Karstaedt, 2009[91]	South Africa (Soweto)	1997-1999	Seasonal	Rate-difference	N	Mortality: all-cause; Hospitalization: all-cause	both rate and number	<65, 65+, all ages
Kessaram, 2015[92]	New Zealand	1990-2008	Seasonal	Regression model with flu proxy (Poisson)	Weekly influenza A and B counts	Mortality: R+C, all-cause	both rate and number	<65, 65+, all ages
Khajehkazemi, 2023[93]	Iran (Kerman)	2006-2012	Seasonal	Regression model without flu proxy (Linear)	N	Mortality: cardiovascular, all-cause	both rate and number	<65, 65-74, 75+, all ages
Khieu, 2015[94]	New Zealand	1994-2008	Seasonal	Regression model with flu proxy (Negative binomial)	Weekly counts of influenza isolates	Hospitalization: cardiovascular, all-cause, all medical illness	both rate and number	<1, 1-4, 5-19, 20-34, 35-49, 50-64, 65-79, 80+, all ages
King, 2014[95]	US (Maryland)	2001-2013	Seasonal and 2009 pandemic	Regression model without flu proxy (Poisson)	N	Hospitalization associated with mechanical ventilator use: all-cause	number	<2, 2-17, 18-49, 50-64, 65+, all ages
Kovács, 2020[96]	Hungary	2009-2017	Seasonal	Regression model with flu proxy (Poisson)	Percentage of ILI consultations in general practices	Mortality: all-cause	both rate and number	0-4, 5-14, 15-64, 65+, all ages
Pedersen, 2024[97]	Denmark	1815-1915	1889 pandemic	Rate-difference	N	Mortality: all-cause	both rate and number	<1, 1-14, 15-39, 40-60, 60+, all ages
Kuo, 2011[98]	Austria	2001-2009	Seasonal	Regression model without flu proxy (Linear)	N	Mortality: all-cause	number	all ages
Kwok, 2017[99]	Hong Kong	1998-2011	Seasonal and 2009 pandemic	Regression model with flu proxy (Linear)	proportion of physician visits for ILI multiplied by the proportion of specimens testing positive for influenza	Mortality: all-cause	rate	all ages
Kwong, 2008[100]	Canada	1997-2004	Seasonal	Regression model with flu proxy (Poisson)	Proportion of specimens testing positive for influenza	Mortality: All-cause	rate	<50, 50–64, 65–74, 75–84, 85+

Kyeyagalire, 2014[101]	South Africa	2007-2012	Seasonal and 2009 pandemic	Regression model with flu proxy (Poisson)	Proportion of specimen testing positive for influenza	Hospitalization: cardiovascular	both rate and number	<1, 1-4, 5-19, 20-44, 45-64, 65-74, 75+, all ages
Kyncl, 2005[102]	Czech Republic	1982-2000	Seasonal	Survival regression model	N	Mortality: cardiovascular, all-cause	both rate and number	all ages
Kyncl, 2025[103]	Czech Republic	1998-2020	Both	Regression model without flu proxy (GAM)	N	Mortality: all-cause	number	40-44, 45-49, 50-54, 55-59, 60-64, 65-69, 70-74, 75-79, 80-84, 85-89, 90-94, 40-94
López-Cuadrado, 2012[104]	Spain	1999-2005	Seasonal	Regression model with flu proxy (Poisson); Regression model without flu proxy (Linear)	Proportion of specimens testing positive for influenza; N	Mortality: All-cause	both rate and number	45-64, 65+, 45+
Langmuir, 1964[105]	US	1934-1963	Seasonal and 1957 pandemic	Regression model without flu proxy (Linear)	N	Mortality: all-cause	both rate and number	all ages
Ledberg, 2021[106]	Sweden	1860-2020	Both	Regression model without flu proxy (Poisson)	N	Mortality: All-cause	both rate and number	all ages
León-Gómez, 2015[107]	Spain	2006-2012	Seasonal and 2009 pandemic	Regression model with flu proxy (Poisson)	Weekly incidence of influenza	Mortality: All-cause	both rate and number	65+, all ages
Lee, 2007[108]	Singapore	1917-1970	1918 and 1957 pandemic	Regression model without flu proxy (Linear); Rate-difference	N	Mortality: all-cause	both rate and number	all ages
Lee, 2009[109]	Singapore	1950-2000	Both	Rate-difference	N	Mortality: all-cause	both rate and number	all ages
Lemaitre, 2012[110]	France	1997-2010	Seasonal and 2009 pandemic	Regression model with flu proxy (Poisson); Regression model without flu proxy (Linear)	Weekly ILI incidence; N	Mortality: R+C, all-cause; Years of life lost	both rate and number	0-4, 5-24, 25-44, 45-64, 65+, all ages
Lemaitre, 2022[111]	France	2010-2015 (mortality); 2010-2018 (hospitalization)	Seasonal	Regression model with flu proxy (Poisson)	ILI outpatient consultation rates	Mortality: cardiovascular, all-cause Hospitalization: cardiovascular, all-cause	both rate and number	0-4, 5-19, 20-49, 50-64, 65+
Lenglet, 2007[112]	Spain	2000-2004	Seasonal	Rate-difference	N	Hospitalization: all-cause, heart failure	both rate and number	<5, 65+, all ages

Levi, 2022[113]	Italy	2009-2020	Seasonal and 2009 pandemic	Rate-difference	N	Mortality: All-cause	both rate and number	65+, all ages
Li, 2006[114]	Hong Kong	1999-2000	Seasonal	Regression model with flu proxy (Linear)	Proportion of specimens testing positive for influenza	Mortality: R+C; Hospitalization: R+C	number	all ages
Li, 2021[115]	Mainland China (Shanghai)	2010-2017	Both	Regression model with flu proxy (Negative binomial)	Proportion of specimens testing positive for influenza	Mortality: cardiovascular, R+C; Hospitalization: cardiovascular, R+C	rate	0-5, 6-17, 18-64, 65+, <65, all ages
Li, 2023[116]	Mainland China (Guangzhou)	2015-2018	Seasonal	Regression model with flu proxy (Poisson)	Proportion of physician visits for ILI multiplied by the proportion of specimens testing positive for influenza	Mortality: cardiovascular, R+C, all-cause	both rate and number	<60, 60-79, 80+, all ages
Linhart, 2011[117]	Israel	1999-2006	Seasonal	Rate-difference	N	Mortality: R+C, all-cause	both rate and number	0-49, 50-64, 65+
Liu, 2017[118]	Mainland China (Hefei)	2010-2015	Seasonal	Regression model with flu proxy (Poisson)	Proportion of physician visits for ILI multiplied by the proportion of specimens testing positive for influenza	Mortality: cardiovascular, diabetes, all-cause, IHD, cerebrovascular disease; stroke	rate	all ages
Liu, 2020[119]	Mainland China (Beijing)	2011-2018	Both	Regression model with flu proxy (Poisson)	Proportion of specimens testing positive for influenza	Mortality: cardiovascular, IHD, stroke	both rate and number	65+
Liu, 2024[120]	Hong Kong	2014-2021	Seasonal	Machine learning model with flu proxy (XGBoost)	Proportion of specimens testing positive for influenza	Mortality: R+C, all-cause	both rate and number	0-19, 20-39, 40-64, 65-84, 85+, all ages
Lui, 1987[121]	US	1972-1985	Seasonal	Regression model without flu proxy (Linear)	N	Mortality: all-cause	number	65+, all ages
Mamelund, 2000[122]	Norway	1918-1978	Seasonal and 1918, 1957, 1968 pandemic	Rate-difference	N	Mortality: all-cause	both rate and number	all ages
Markush, 1963[123]	US (Florida)	1957-1960	Seasonal	Rate-difference	N	Mortality: all-cause	both rate and number	all ages

Matias, 2014[124]	US	1997-2009	Seasonal	Regression model with flu proxy (Linear)	Proportion of specimens testing positive for influenza	Mortality: R+C	both rate and number	0-4, 5-17, 18-49, 50-64, 65-74, 75+, all ages
Mazick, 2010[125]	Belgium, Denmark, Greece, Germany (Hesse), Malta, Netherlands, Sweden, Switzerland	2009	2009 pandemic	Regression model without flu proxy (Poisson)	N	Mortality: all-cause	both rate and number	0-4, 5-14, 15-64, 65+
McBean, 1993[126]	US	1989-1991	Seasonal	Rate-difference	N	Hospitalization: cancer, diabetes, all-cause, IHD, congestive heart failure, heart rhythm disorders, occlusive stroke	both rate and number	65+
Miller, 1971[127]	Britain	1966-1970	1968 pandemic	Rate-difference	N	Mortality: all cause; Sickness insurance benefit claims: all-cause	number	all ages
Moa, 2022[128]	Australia	2001-2017	Seasonal	Regression model with flu proxy (GAM)	Counts of lab-confirmed influenza cases	Mortality: cardiovascular, myocardial infarction; Hospitalization: cardiovascular, myocardial infarction	both rate and number	50-64, 65+
Mølbak, 2011[129]	Denmark	2005-2009	Seasonal and 2009 pandemic	Regression model with flu proxy (Poisson)	Proportion of ILI consultations	Mortality: all-cause	number	<1, 1-4, 5-14, 15-64, 65+, all ages
Molbak, 2015[130]	Europe	2014-2015	Seasonal	Regression model without flu proxy (Poisson)	N	Mortality: all-cause	proportion	65+
Muñoz, 2011[131]	Spain (Catalonia)	1994-2007	Seasonal	Regression model with flu proxy (Negative binomial)	Counts of lab-confirmed influenza cases	Mortality: all-cause	number	all ages
Murray, 2006[132]	Argentina, Australia, Austria, Belgium, Canada, Chile, Denmark, England, Finland, France, Germany, India,	1918-1920	1918 pandemic	Rate-difference	N	Mortality: all-cause	both rate and number	0-4, 5-9, 10-14, 15-19, 20-24, 25-29, 30-34, 35-39, 40-44, 45-49, 50-54, 55-59, 60-64, 65-69, 70-74, all ages

	Italy, Japan, Netherlands, New Zealand, Norway, Philippines, Portugal, Spain, Sri Lanka, Sweden, Switzerland, Taiwan, Uruguay, US, Venezuela							
Muscatello, 2010[133]	Australia (New South Wales)	2003-2009	2009 pandemic	Regression model without flu proxy (Linear)	N	Mortality: all-cause	both rate and number	0-19, 20-49, 50-64, 65-79, 80+, all ages
Muscatello, 2014[134]	Australia	2003-2009	Seasonal and 2009 pandemic	Regression model with flu proxy (GAM); Regression model with flu proxy (Linear)	Counts of lab-confirmed influenza cases	Mortality: R+C, all-cause	both rate and number	<65, 65+, all ages
Muscatello, 2017[135]	Australia (New South Wales)	2010-2014	Seasonal	Regression model with flu proxy (GAM)	Counts of lab-confirmed influenza cases	ED visit: cardiovascular, all-cause, infection	both rate and number	0-4, 5-14, 15-49, 50-64, 65-84, 85+, all ages
Muscatello, 2021[136]	Australia	2010-2019	Both	Regression model with flu proxy (Poisson)	Weekly counts of each of positive influenza results	Mortality: all-cause	rate	<65, 65-74, 75+, all ages
Narayan, 2020[137]	India	2010-2013	Both	Regression model with flu proxy (Negative binomial)	Proportion of specimens testing positive for influenza	Mortality: cardiovascular, R+C	both rate and number	<5, 5-64, 65+, all ages
Neuzil, 2000[138]	US (Tennessee)	1973-1993	Seasonal	Rate-difference	N	Hospitalization: R+C; Outpatient visit: all-cause; Courses of antibiotics: all-cause	rate	0-0.5, 0.5-1, 1-3, 3-5, 5-15, <15
Newall, 2008[139]	Australia	1997-2004 (mortality); 1998-2005 (hospitalization)	Seasonal	Regression model with flu proxy (GLM)	Monthly laboratory specimen counts	Mortality: cardiovascular, all-cause; Hospitalization: cardiovascular	rate	50-64, 65+
Newall, 2010[140]	Australia	1997-2004	Seasonal	Regression model with flu proxy (Poisson); Regression model without flu proxy (Linear)	Monthly laboratory specimen counts; N	Mortality: cardiovascular, all-cause	both rate and number	50-64, 65-74, 75-84, 85+

Nicholson, 1996[141]	England and Wales	1975-1990	Seasonal	Regression model with flu proxy (Linear)	RCGP consultation rate for aggregated URTI and 4-weekly influenza A+B reports	Mortality: all-cause	number	all ages
Nielsen, 2011[142]	Denmark	1994-2010	Seasonal and 2009 pandemic	Regression model with flu proxy (Poisson)	ILI consultation percentage after seasonal correction	Mortality: all-cause	both rate and number	0-4, 5-14, 15-64, 65+, all ages
Nielsen, 2013[143]	Europe	2008-2011	Seasonal and 2009 pandemic	Regression model without flu proxy (Poisson)	N	Mortality: all-cause	proportion	0–4, 5–14, 15–64, 65+, all ages
Nielsen, 2018[144]	Denmark	2010-2017	Seasonal	Regression model with flu proxy (Poisson)	Proportion of physician visits for ILI multiplied by the proportion of specimens testing positive for influenza	Mortality: all-cause	both rate and number	0-4, 5-14, 15-64, 65+, all ages
Nielsen, 2019[145]	Europe	2012-2018	Seasonal	Regression model with flu proxy (Poisson)	Proportion of physician visits for ILI multiplied by the proportion of specimens testing positive for influenza	Mortality: all-cause	rate	0-4, 5-14, 15-64, 65+, all ages
Nogueira, 2009[146]	Portugal	2007-2009	Seasonal	Regression model without flu proxy (Linear)	N	Mortality: all-cause	both rate and number	65-74, 75+, all ages
Nørgaard, 2026[147]	Europe (16 participating countries)	2014-2019	Seasonal	Regression model without flu proxy (Poisson)	N	Mortality: all-cause	rate	65+, all ages
Nunes, 2011[148]	Portugal	1980-2004	Seasonal	ARIMA	N	Mortality: all-cause, IHD, cerebrovascular disease	both rate and number	0-4, 5-54, 55-64, 65-69, 70-74,75-79, 80-84, 85+, 65+, all ages
Nunes, 2018[149]	Portugal	1916-1922	1918 pandemic	Regression model without flu proxy (Negative binomial)	N	Mortality: all-cause	both rate and number	all ages
Nygaard, 2023[150]	Norway (Kautokeino, Karasjok)	1918-1920	1918 pandemic	Regression model without flu proxy (Linear)	N	Mortality: all-cause	rate	0–19, 20–49, 50+
Ohmi, 2011[151]	Japan	1952-2009	Seasonal and 2009 pandemic	Relative mortality distribution model	N	Mortality: all-cause	both rate and number	0-4, 5-14, 15-34, 35-64, 65+, all ages
Ortiz, 2014[152]	US (Arizona, California, Washington)	2003-2009	Seasonal	Regression model with flu proxy (Negative binomial)	Proportion of specimens testing positive for influenza	Hospitalization: R+C, critical illness	number	18-49, 50-64, 65-74, 75-84, 85+, 18+

Park, 2016[153]	South Korea	2003-2013	Seasonal and 2009 pandemic	Regression model with flu proxy (GAM)	Proportion of physician visits for ILI multiplied by the proportion of specimens testing positive for influenza	Mortality: cardiovascular, cancer, diabetes, renal diseases, chronic liver diseases, degenerative diseases of the nervous system, all-cause, heart disease, cerebrovascular disease	both rate and number	0-4, 5-14, 15-44, 45-64, 65+, all ages
Pebody, 2015[154]	England	2014-2015	Seasonal	Regression model without flu proxy (Poisson)	N	Mortality: all-cause	rate	<18
Pebody, 2018[155]	England	2008-2015	Seasonal and 2009 pandemic	Regression model with flu proxy (Poisson)	Proportion of physician visits for ILI multiplied by the proportion of specimens testing positive for influenza	Mortality: all-cause	number	<5, 5-14, 15-64, 65+, all ages
Perdelli, 1980[156]	Italy (Genoa)	1956-1979	Seasonal	Regression model without flu proxy (Linear)	N	Mortality: all-cause	rate	all ages
Portugal, 2021[157]	Portugal	2009-2020	Seasonal	Regression model with flu proxy (Negative binomial)	Counts of lab-confirmed influenza cases	Mortality: all-cause	number	all ages
Presanis, 2011[158]	England	2009-2010	2009 pandemic	Multiplier	N	Mortality: all-cause; Hospitalization: all-cause; ICU admission: all-cause	number	<1, 1-4, 5-14, 15-24, 25-44, 45-64, 65+
Pumarola, 2023[159]	Spain	2008-2018	Seasonal	Regression model with flu proxy (Poisson)	ILI incidence rate	Mortality: R+C, All-cause; Hospitalization: R+C, All-cause	both rate and number	0-4, 5-18, 19-49, 50-64, 65-74, 75+, 65+, all ages
Qi, 2020[160]	Mainland China (Chongqing)	2012-2018	Seasonal and 2009 pandemic	Regression model with flu proxy (Negative binomial)	Proportion of specimens testing positive for influenza	Mortality: R+C, all-cause, IHD	both rate and number	<65, 65+, all ages
Quandelacy, 2014[161]	US	1997-2007	Seasonal	Regression model with flu proxy (Linear)	Proportion of physician visits for ILI multiplied by the proportion of specimens testing	Mortality: cardiovascular, cancer, diabetes, renal diseases, chronic liver	rate	<18, 18-49, 50-64, 65-74, 75+, all ages

					positive for influenza	diseases, degenerative diseases of the nervous system, septicaemia, all-cause, heart disease, myocardial infarction, IHD, cerebrovascular disease, central nervous system disease, Alzheimer disease, unintentional injuries		
Ragona, 1978[162]	Italy (Rome)	1956-1976	Seasonal	Regression model without flu proxy (Linear)	N	Mortality: all-cause Absenteeism: all-cause	rate	all ages
Ramírez, 2009[163]	Colombia (Bogotá)	1997-2005	Seasonal	Regression model without flu proxy (Poisson)	N	Mortality: cardiovascular, all-cause	rate	60+, all ages
Ramiro, 2018[164]	Spain (Madrid)	1888-1892	1889 pandemic	Rate-difference	N	Mortality: all-cause	rate	<5, 5-14, 15-24, 25-49, 50-69, 70+, all ages
Reed, 2015[5]	United States	2010-2013	Seasonal	Multiplier	hospital admissions due to lab-confirmed influenza	Mortality: all-cause; Hospitalization: all-cause; ICU admission: all-cause	both rate and number	<18, 18-64, 65+, all ages
Reichert, 2001[165]	Japan, US	1949-1998	Seasonal	Rate-difference	N	Mortality: all-cause	rate	all ages
Reichert, 2004[166]	US	1959-1999	Seasonal and 1968 pandemic	ARIMA; Regression model without flu proxy (Linear)	N	Mortality: cancer, diabetes, all-cause, IHD, cerebrovascular disease	both rate and number	<75, 75+, all ages
Reyes, 2018[167]	India	1916-1920	1918 pandemic	Regression model without flu proxy (Linear)	N	Mortality: all-cause	proportion	<5, 5-20, 20-30, 30-50, 50+
Richard, 2009[168]	Japan, US, UK	1915-1923	1918 pandemic	Regression model without flu proxy (Linear); Rate-difference	N	Mortality: all-cause	both rate and number	all ages
Ridenhour, 2013[169]	Canada (Ontario)	1993-2008	Seasonal	Regression model with flu proxy (Linear)	Proportion of specimens testing positive for influenza	Mortality: all-cause	number	65-74, 75+

Rizzo, 2006[170]	Italy	1970-2001	Seasonal	Regression model without flu proxy (Linear)	N	Mortality: all-cause	both rate and number	45-64, 65+
Rizzo, 2007[171]	Italy	1969-2001	Both	Regression model without flu proxy (Linear)	N	Mortality: all-cause	both rate and number	0-14, 15-44, 45-64, 65+, all ages
Rosano, 2019[172]	Italy	2013-2017	Seasonal	Regression model with flu proxy (Poisson)	Proportion of physician visits for ILI multiplied by the proportion of specimens testing positive for influenza	Mortality: all-cause	both rate and number	0-4, 5-14, 15-64, 65+, all ages
Rubinson, 2013[173]	US	2003-2009	2009 pandemic	Rate-difference	N	Hospitalization: all-cause;<br>ED visit: all-cause	number	all ages
Sánchez-Ramos, 2017[174]	Mexico	1998-2012	Seasonal	Regression model without flu proxy (Linear)	N	Mortality: cardiovascular, all-cause	both rate and number	0-6 months, 6-11 months, 12-23 months, 24-35 months, 36-60 months, 0-60 months
Salto-Quintana, 2019[175]	Mexico	1998-2015	Seasonal and 2009 pandemic	Regression model without flu proxy (Linear)	N	Mortality: cardiovascular, all-cause	rate	0-4, 5-19, 20-59, 60+, all ages
Sattenspiel, 2025[176]	US (Alaska)	1915-1921	1918 pandemic	Regression model without flu proxy (Linear)	N	Mortality: all-cause	both rate and number	0-4, 5-9, 10-14, 15-19, 20-24, 25-29, 30-34, 35-39, 40-44, 45-49, 50-54, 55-59, 60-64, 65-69, 70-74, 75+, all ages
Schanzer, 2007[177]	Canada	1989-1999	Seasonal	Regression model with flu proxy (Poisson)	Influenza-certified death numbers	Mortality: cancer, renal diseases, all-cause, IHD, other heart disease, stroke, metabolic, dementia, diseases of digestive system, other cardiovascular, disorders of the nervous system, psychotic conditions, accidents	both rate and number	<50, 50-64, 65+, all ages
Schanzer, 2008[178]	Canada	1994-2000	Seasonal	Regression model with flu proxy (Poisson)	Hospital admissions due to	Mortality: cardiovascular, all-	both rate and number	<50, 50-54, 55-59, 60-64, 65-69, 70-74, 75-79, 80-84,

					lab-confirmed influenza	cause, heart disease		85-89, 90+, 65+, all ages
Schanzer, 2013[179]	Canada	1992-2009	Seasonal and 2009 pandemic	Regression model with flu proxy (Poisson)	Flu A: laboratory positive tests and admissions to hospital with laboratory-confirmed influenza;<br>Flu B: percentage of laboratory tests positive	Mortality: all-cause;<br>Hospitalization: all-cause	both rate and number	<65, 65+, all ages
Schanzer, 2013[180]	Canada (Ontario)	2003-2010	Seasonal and 2009 pandemic	Regression model with flu proxy (Poisson)	Hospital admissions due to lab-confirmed influenza	ED visit: all-cause	rate	all ages
Schindler, 2024[181]	Germany	1996-2018	Seasonal and 2009 pandemic	Relative mortality distribution model	N	Mortality: cardiovascular, R+C, all-cause;<br>Hospitalization: all-cause	both rate and number	0-17, 18-34, 35-59, 60-69, 70-79, 80-89, 90+, all ages
Schmidt, 2022[182]	Denmark, Spain, US	2015-2017	Seasonal	Regression model without flu proxy (Poisson)	N	Mortality: cardiovascular, R+C, all-cause	both rate and number	0-64, 65-74, 75+, all ages
Shindo, 2000[183]	Japan	1947-1999	Seasonal	ARIMA	N	Mortality: all-cause	number	all ages
Shrestha, 2011[184]	US	2009-2010	2009 pandemic	Multiplier	N	Mortality: all-cause;<br>Hospitalization: all-cause	both rate and number	0-17, 18-64, 65+, all ages
Simonsen, 1997[185]	US	1972-1992	Seasonal	Regression model without proxy (Linear)	N	Mortality: all-cause	number	all ages
Simonsen, 2005[186]	US	1968-2001	Both	Regression model without proxy (Linear)	N	Mortality: all-cause	number	45-64, 65-69, 70-74, 75-79, 80-84, 90-94, 65+, 85+
Simonsen, 2013[187]	Argentina, Australia, Chile, Mainland China, France, Germany, Hong Kong, Israel, Japan, Mexico, New Zealand, Poland, Republic of Korea, Romania,	2005-2009	2009 pandemic	Regression model with flu proxy (Linear) and multiple imputation model for extrapolation	Counts of lab-confirmed influenza cases	Mortality: R+C, all-cause	both rate and number	<65, 65+, all ages

	Singapore, Slovenia, South Africa, Spain, UK, US							
Sinnathamby, 2023[188]	UK (Scotland, England, Wales), Northern Ireland, Republic of Ireland	2010-2017	Seasonal	Regression model with flu proxy (Poisson)	Proportion of physician visits for ILI multiplied by the proportion of specimens testing positive for influenza	Mortality: all-cause	both rate and number	<15, 15-64, 65+, all ages
Sinnecker, 1987[189]	Germany	1968-1985	Seasonal	Regression model without flu proxy (Linear)	N	Mortality: cardiovascular, cancer, diabetes, renal diseases, all-cause, heart disease, IHD, cerebrovascular disease, hypertension, artery disease	both rate and number	all ages
Somayaji, 2020[190]	US (Washington)	2001-2012	Seasonal and 2009 pandemic	Regression model with flu proxy (Negative binomial)	Proportion of specimens testing positive for influenza	Hospitalization: R+C	both rate and number	0-0.5, 0.5-2, 2-4, 5-14, 15-49, 50-64, 65+, all ages
Sprenger, 1993[191]	Netherlands	1967-1989	Seasonal	Regression model with flu proxy (Poisson)	Influenza mortality rates	Mortality: heart disease, heart and lung diseases, all other diseases beyond heart and lung diseases	both rate and number	0-59, 60-69, 70-79, 80+
Staub, 2022[192]	Switzerland, Sweden, Spain	1877-2021	1890, 1918 and 1957 pandemic	Regression model without proxy (Negative binomial)	N	Mortality: all-cause	both rate and number	all ages
Steele, 2026[193]	48 populations	1918, 1957, 1968 and 2009	Pandemic	Regression model without flu proxy (Linear)	N	Mortality: all-cause	rate	0-4, 5-24, 25-39, 40-64, 65+
Stein, 2024[194]	Australia	2015-2019	Seasonal	Regression model with flu proxy (Poisson)	Counts of lab-confirmed influenza cases	Mortality: all-cause	number	<65
Stroup, 1988[195]	US	1962-1983	Seasonal and 1968 pandemic	ARIMA	N	Mortality: all-cause	rate	<1, 1-14, 15-44, 45-64, 65+, all ages
Sugaya, 2005[196]	Japan	1972-2003	Seasonal	Rate-difference	N	Mortality: all-cause	both rate and number	0-1, 1-4, 5-9, 10-14

Suter, 2024[197]	Switzerland	1889-1994	1918, 1957, 1968 pandemic	Regression model without flu proxy (Poisson)	N	Mortality: all-cause	rate	all ages
Tachibana, 1999[198]	Japan	1980-1994	Seasonal	Relative mortality distribution model	N	Mortality: cancer, diabetes, renal diseases, chronic liver diseases, all-cause, IHD, cerebrovascular disease, mental disease, hypertensive disease	rate	0-4, 5-9, 10-14, 15-19, 20-29, 30-44, 45-64, 65-74, 75+, all ages
Takahashi, 2001[199]	Japan	1975-1997	Both	Relative mortality distribution model; Rate-difference	N	Mortality: all-cause	number	all ages
Takahashi, 2002[200]	Japan	1975-1999	Seasonal	Relative mortality distribution model	N	Mortality: cancer, diabetes, chronic liver diseases, all-cause, heart disease, cerebrovascular disease, hypertensive diseases, renal failure	both rate and number	0-4, 5-24, 25-44, 45-64, 65+, all ages
Takahashi, 2006[201]	Japan	1987-2003	Pandemic	Relative mortality distribution model	N	Mortality: all-cause	number	all ages
Takahashi, 2008[202]	Japan	1987-2005	Seasonal	Relative mortality distribution model	N	Mortality: cancer, renal diseases, all-cause, heart disease, cerebrovascular disease	number	0-4, 5-24, 25-44, 45-64, 65+, all ages
Tayback, 1957[203]	Philippines (Manila)	1956-1957	1957 pandemic	Rate-difference	N	Mortality: all-cause	both rate and number	0-1, 1-4, 5-9, 10-14, 15-24, 25-44, 45-64, 65+, all ages
Tempia, 2015[204]	South Africa	1998-2009	Seasonal and 2009 pandemic	Regression model with flu proxy (Poisson)	Proportion of specimens testing positive for influenza	Mortality: cardiovascular, all-cause	both rate and number	5-19, 20-44, 45-64, 65-74, 75+, 5+
Tempia, 2019[205]	South Africa	2013-2015	Both	Regression model with flu proxy (GAM)	Proportion of specimens testing positive for influenza	Mortality: cardiovascular, all-cause, all other causes other than respiratory and cardiovascular; Medically-attended	both rate and number	<1, 1–4, 5–24, 25–44, 45–64, 65+, <5, 5+, all ages

						illness and severe illness: cardiovascular, all-cause, all other causes other than respiratory and cardiovascular		
Thiberville, 2015[206]	South-eastern France	1999-2010	Seasonal and 2009 pandemic	Regression model with flu proxy (Poisson)	ILI outpatient consultation rates	Mortality: cardiovascular, all-cause	rate	0–24, 25–44, 45–64, 65+
Thompson, 2003[207]	US	1990-1999	Seasonal	Regression model with flu proxy (Poisson)	Proportion of specimens testing positive for influenza	Mortality: R+C, all-cause	both rate and number	<1, 1-4, 5-49, 50-64, 65+, all ages
Thompson, 2004[208]	US	1979-2001	Seasonal	Regression model with flu proxy (Poisson)	Proportion of specimens testing positive for influenza	Hospitalization: R+C	both rate and number	<5, 5-49, 50-64, 65-69, 70-74, 75-79, 80-84, 85+, all ages
Thompson, 2009[209]	US	1972-2003	Seasonal	Rate-difference; Regression model without flu proxy (Linear); Regression model with flu proxy (Poisson); ARIMA	(For Poisson model) Proportion of specimens testing positive for influenza	Mortality: R+C	both rate and number	<65, 65+, all ages
Tillett, 1980[210]	England and Wales	1968-1979	Seasonal	Regression model with flu proxy (Linear)	RGCP reported influenza rates	Mortality: all-cause; Sickness benefit claims: all-cause	number	65+, all ages
Tillett, 1983[211]	England and Wales	1968-1978	Seasonal	Regression model with flu proxy (Linear)	Rate of clinical epidemic influenza reported by GP	Mortality: cardiovascular, R+C, all-cause, IHD, cerebrovascular disease	number	<14, 15-39, 40-64, 65+, all ages
Togashi, 2004[212]	Japan (Hokkaido)	1994-2002	Seasonal	Not used	N	Complication: acute encephalopathy	both rate and number	<15
Tominaga, 1977[213]	Japan	1961-1974	Seasonal	Regression model without flu proxy (Linear)	N	Mortality: diabetes	rate	all ages
US CDC, 2010[214]	US	1976-2007	Seasonal	Regression model with flu proxy (Poisson)	Proportion of specimens testing positive for influenza	Mortality: R+C	both rate and number	<19, 19-64, 65+, all ages
vanAsten, 2012[215]	Netherlands	1999-2007	Seasonal	Regression model with flu proxy (Poisson)	Number of lab reports for each pathogen	Mortality: all-cause	both rate and number	65-74, 75-84, 85+, 65+

VanAsten, 2021[216]	Netherlands	2010-2020	Both	Regression model without flu proxy (Linear)	N	Mortality: all-cause	number	all ages
Vestergaard, 2017[217]	Europe	2016-2017	Seasonal	Regression model with flu proxy (Poisson)	Reported rates of ILI or ARI	Mortality: all-cause	rate	0-4, 5-14, 15-64, 65+, all ages
Viboud, 2005[218]	US, Canada, England and Wales, France, Japan, Australia	1967-1978	1968 Pandemic	Regression model without flu proxy (Linear)	N	Mortality: all-cause	rate	<65, all ages
Viboud, 2006[219]	England and Wales, Canada, US	1950-1999	Seasonal and 1951, 1957, 1968 pandemic	Regression model without flu proxy (Linear)	N	Mortality: all-cause	rate	<65, 65+, all ages
Viboud, 2016[220]	Europe, the Asia-Pacific region, the Americas	1957-1959	1957 pandemic	Regression model without flu proxy (Spline; linear)	N	Mortality: R+C	both rate and number	all ages
Walkowiak, 2023[221]	Poland	2000-2019	Seasonal	Regression model without flu proxy (Poisson)	N	Mortality: cardiovascular, cancer, diabetes, septicaemia, IHD, cerebrovascular disease, atherosclerosis	number	all ages
Wang, 2014[222]	Mainland China (Guangzhou)	2010-2012	Both	Regression model with flu proxy (Negative binomial)	Proportion of specimens testing positive for influenza	Mortality: R+C, all-cause, IHD	both rate and number	0-64, 65+, all ages
Warren-Gash, 2011[223]	England and Wales, Hong Kong	1998-2008	Seasonal	Regression model with flu proxy (Poisson)	GP consultations for ILI (for England and Wales) and proportion positive for Hong Kong	Mortality: myocardial infarction; Hospitalization: myocardial infarction	number	all ages
Wijngaard, 2012[224]	Netherlands	1999-2010	Seasonal and 2009 pandemic	Regression model with flu proxy (Poisson)	ILI incidence	Mortality: all-cause	both rate and number	0-4, 5-24, 25-44, 45-64, 65-74, 75+, all ages
Wong, 2004[225]	Hong Kong	1996-1999	Seasonal	Regression model with flu proxy (Poisson); Rate-difference	Proportion of specimens testing positive for influenza; N	Mortality: R+C, all-cause, IHD	both rate and number	40-64, 65+, all ages
Wong, 2009[226]	Hong Kong	1996-2000	Seasonal	Regression model with flu proxy (Poisson)	Proportion of specimens testing positive for influenza	Hospitalization: diabetes, IHD, cerebrovascular disease	both rate and number	0-14, 15-39, 40-64, 65-74, 75+, all ages
Wong, 2012[227]	Hong Kong, Singapore,	2004-2006	Seasonal	Regression model with flu proxy (Poisson)	proportion of specimens testing	Mortality: cardiovascular, all-cause, IHD	rate	65+, all ages

	Mainland China (Guangzhou)				positive for influenza			
Wong, 2013[228]	Hong Kong	2001-2009	2009 Pandemic	Regression model with flu proxy (Time series; Linear; Poisson with log/identity links)	Age-standardized Incidence Proxy; ILI; ILI based on consultations with general practitioners; ILI × LAB	Mortality: all-cause	number	0-4, 5-14, 15-29, 30-39, 40-49, 50-59, 60-69, 70+, all ages
Wong, 2019[229]	Hong Kong	2006-2016	Seasonal and 2009 pandemic	Regression model with flu proxy (Linear)	Proportion of physician visits for ILI multiplied by the proportion of specimens testing positive for influenza	Mortality: all-cause	both rate and number	0-4, 5-14, 15-44, 45-64, 65+, all ages
Wong, 2022[3]	Hong Kong	1998-2019	Seasonal and 2009 pandemic	Regression model with flu proxy (Linear)	Proportion of physician visits for ILI multiplied by the proportion of specimens testing positive for influenza	Mortality: septicaemia; Hospitalization: septicaemia	rate	0-4, 5-64, 65+, all ages
Wong, 2026[230]	Hong Kong	2014-2023	Seasonal	Regression model with flu proxy (Linear)	Proportion of physician visits for ILI multiplied by the proportion of specimens testing positive for influenza	Mortality: cardiovascular, heart, cerebrovascular, renal, other, all-cause	both rate and number	0-4, 5-14, 15-44, 45-64, 65+, all ages
Wu, 2012[2]	Hong Kong	1998-2009	Seasonal and 2009 pandemic	Regression model with flu proxy (Linear)	Proportion of physician visits for ILI multiplied by the proportion of specimens testing positive for influenza	Mortality: cardiovascular, malignant neoplasm, diabetes, renal diseases, chronic liver diseases, all-cause, heart disease, cerebrovascular disease, Alzheimer diseases	rate	0-4, 5-14, 15-44, 45-64, 65+, all ages
Wu, 2014[231]	Hong Kong	2009-2011	Seasonal and 2009 pandemic	Regression model with flu proxy (Linear)	Proportion of physician visits for ILI multiplied by the proportion of specimens testing positive for influenza	Mortality: all-cause	rate	0-64, 65+, all ages

Wu, 2018[232]	Mainland China (Beijing)	2007-2013	Seasonal and 2009 pandemic	Regression model with flu proxy (Negative binomial)	Proportion of specimens testing positive for influenza	Mortality: R+C, all-cause	both rate and number	<65, 65+, all ages
Yang, 2009[233]	Hong Kong	1996-2002	Seasonal	Regression model with flu proxy (Poisson)	Proportion of specimens testing positive for influenza	Mortality: R+C, all-cause, IHD, cerebrovascular disease	proportion	65+, all ages
Yang, 2011[234]	Mainland China (Guangzhou), Hong Kong, Singapore	2004-2006	Seasonal	Regression model with flu proxy (Poisson)	Proportion of specimens testing positive for influenza	Mortality: R+C, all-cause, IHD	both rate and number	65+, all ages
Yang, 2012[235]	Hong Kong	2005-2010	Seasonal and 2009 pandemic	Regression model with flu proxy (Poisson)	Proportion of specimens testing positive for influenza	Hospitalization: cardiovascular, diabetes, stroke, IHD	both rate and number	40-64, 65-74, 75+, all ages
Yang, 2012[236]	Hong Kong	1998-2009	Seasonal and 2009 pandemic	Regression model with flu proxy (Poisson)	Proportion of specimens testing positive for influenza	Mortality: R+C, all-cause	both rate and number	<20, 20–39, 40–64, 65–84, 85+, all ages
Yang, 2019[237]	Hong Kong, Brisbane	2001-2012	Seasonal	Regression model with flu proxy (Poisson)	Proportion of specimens testing positive for influenza	Mortality: R+C, all-cause, IHD, stroke; Hospitalization: R+C, IHD, stroke	both rate and number	65+
Yap, 2004[238]	Hong Kong	1998-2001	Seasonal	Regression model with flu proxy (Linear)	Total number of positive respiratory isolates	Hospitalization: heart failure	both rate and number	65+
Yin, 2024[239]	Mainland China	2012-2021	Seasonal	Regression model with flu proxy (Negative binomial)	Proportion of specimens testing positive for influenza	Mortality: cardiovascular, R+C, cancer, diabetes, all-cause, IHD, nervous system diseases, chronic liver and kidney disease	both rate and number	all ages
Yu, 2013[240]	Mainland China	2004-2010	Seasonal and 2009 pandemic	Regression model with flu proxy (Negative binomial)	Proportion of specimens testing positive for influenza	Mortality: R+C, all-cause	both rate and number	0-64, 65+, all ages
Zhang, 2018[241]	Mainland China (Yancheng)	2011-2015	Both	Regression model with flu proxy (Linear)	Proportion of physician visits for ILI multiplied by the proportion of specimens testing positive for influenza	Mortality: R+C, all-cause	rate	0-14, 15-64, 65+, all ages
Zhao, 2019[242]	Mainland China (Shanxi)	2013-2017	Both	Regression model with flu proxy (GLM/Poisson);	Proportion of specimens testing positive for	Mortality: R+C, all-cause	rate	0-64, 65-74, 75+, all ages

				Regression model without flu proxy (Linear)	influenza; N			
Zhou, 2012[243]	US	1993-2008	Seasonal	Regression model with flu proxy (Negative binomial)	Proportion of specimens testing positive for influenza	Hospitalization: R+C	rate	<1, 1-4, 5-49, 50-64, 65+, all ages
Ziegler, 2025[244]	Switzerland (Zurich)	1918-1920	1918 pandemic	Regression model without flu proxy (Poisson)	N	Mortality: all-cause	rate	all ages
Zucs, 2005[245]	Germany	1985-2001	Seasonal	Relative mortality distribution model; Regression model without flu proxy (Linear)	N	Mortality: all-cause	both rate and number	all ages

Supplementary Figures

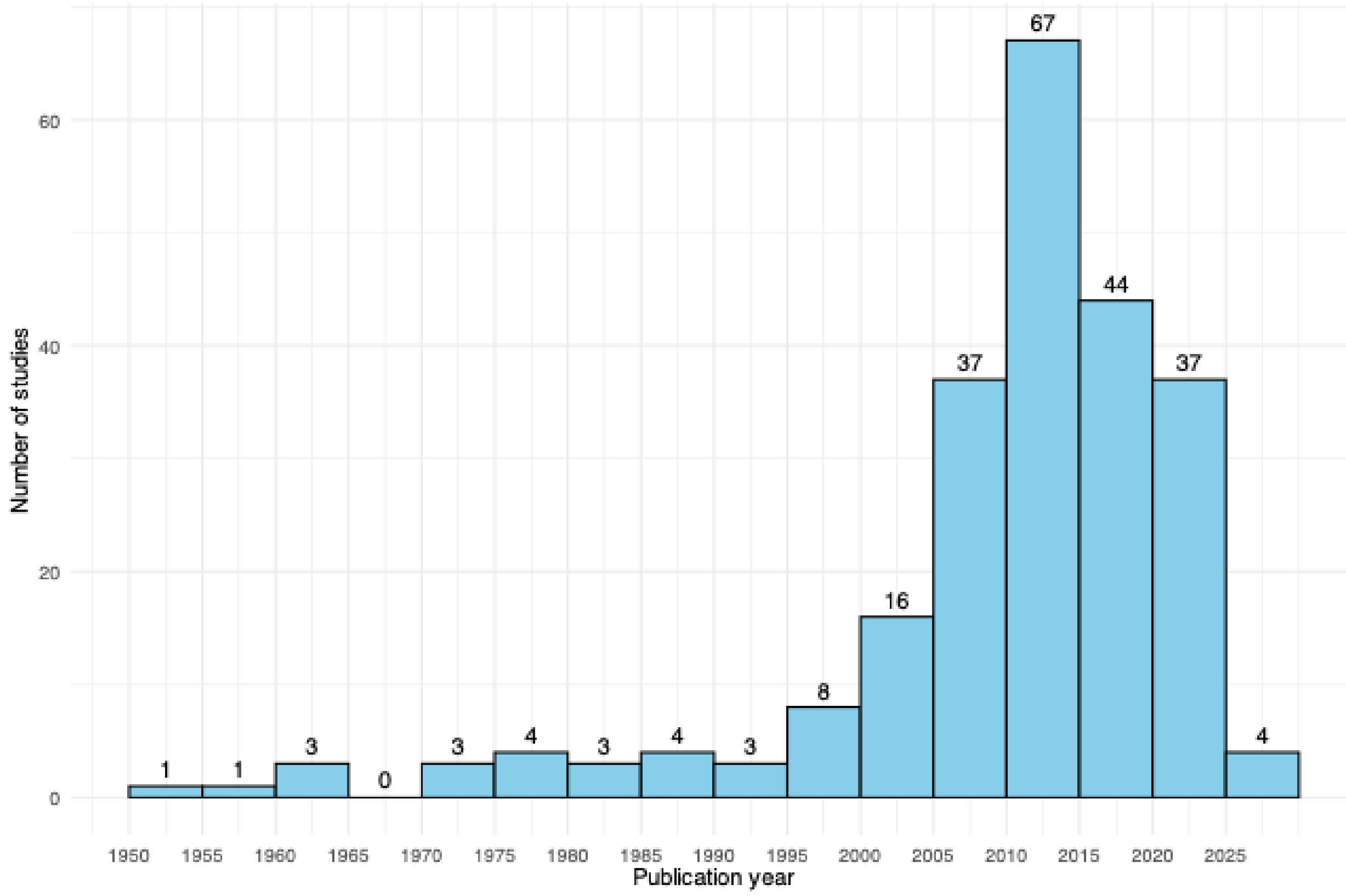

Fig S1. Number of studies by year of publication.

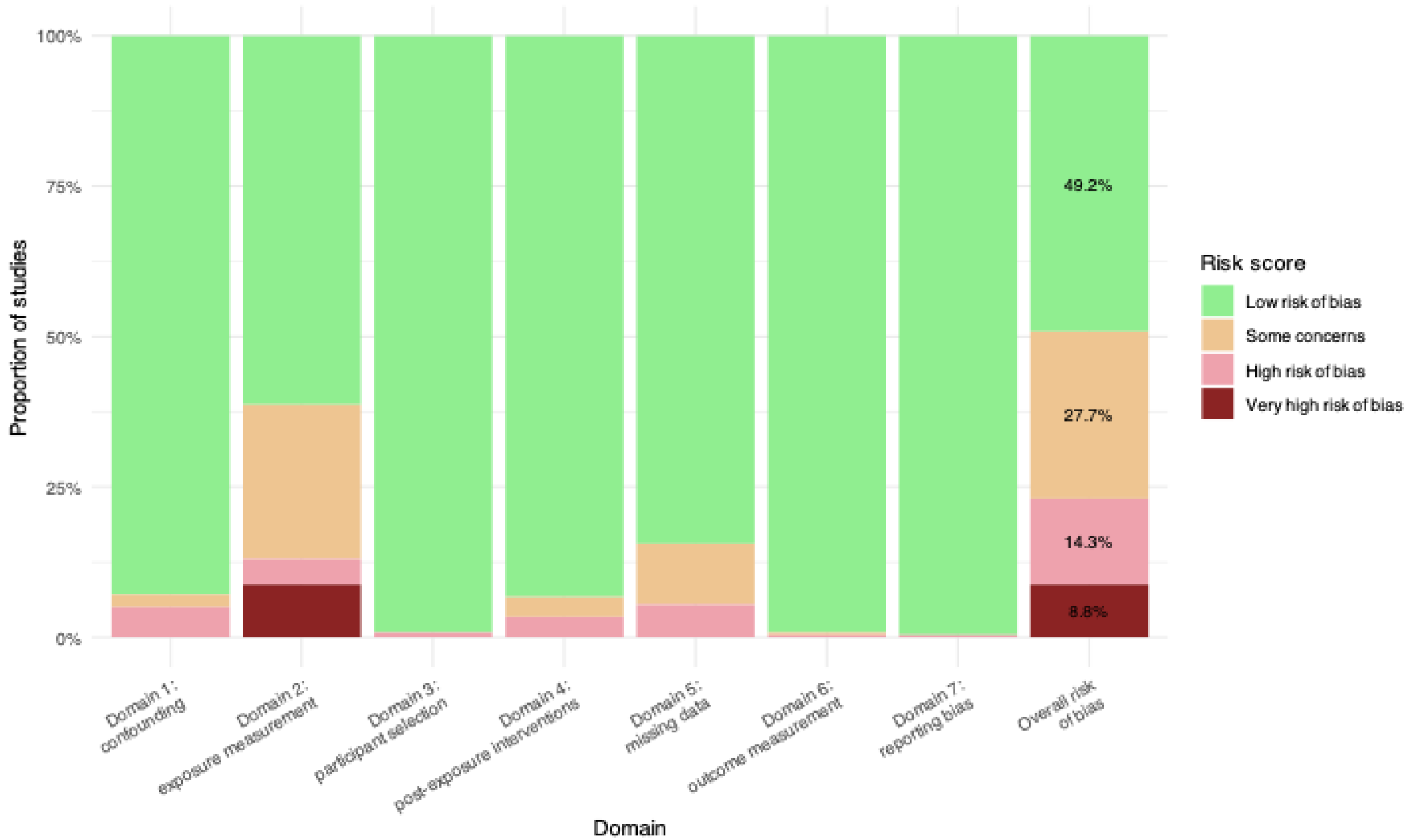

Fig S2. Summary of the results of quality assessment using ROBINS-E in terms of score distribution in each of the seven domains and the overall score. Each bar shows the proportion of included studies rated as low risk, some concerns, high risk, or very high risk of bias within that ROBINS-E domain. The "Overall risk of bias" bar represents the overall risk-of-bias judgement for each study. Percentages are calculated using all included studies.

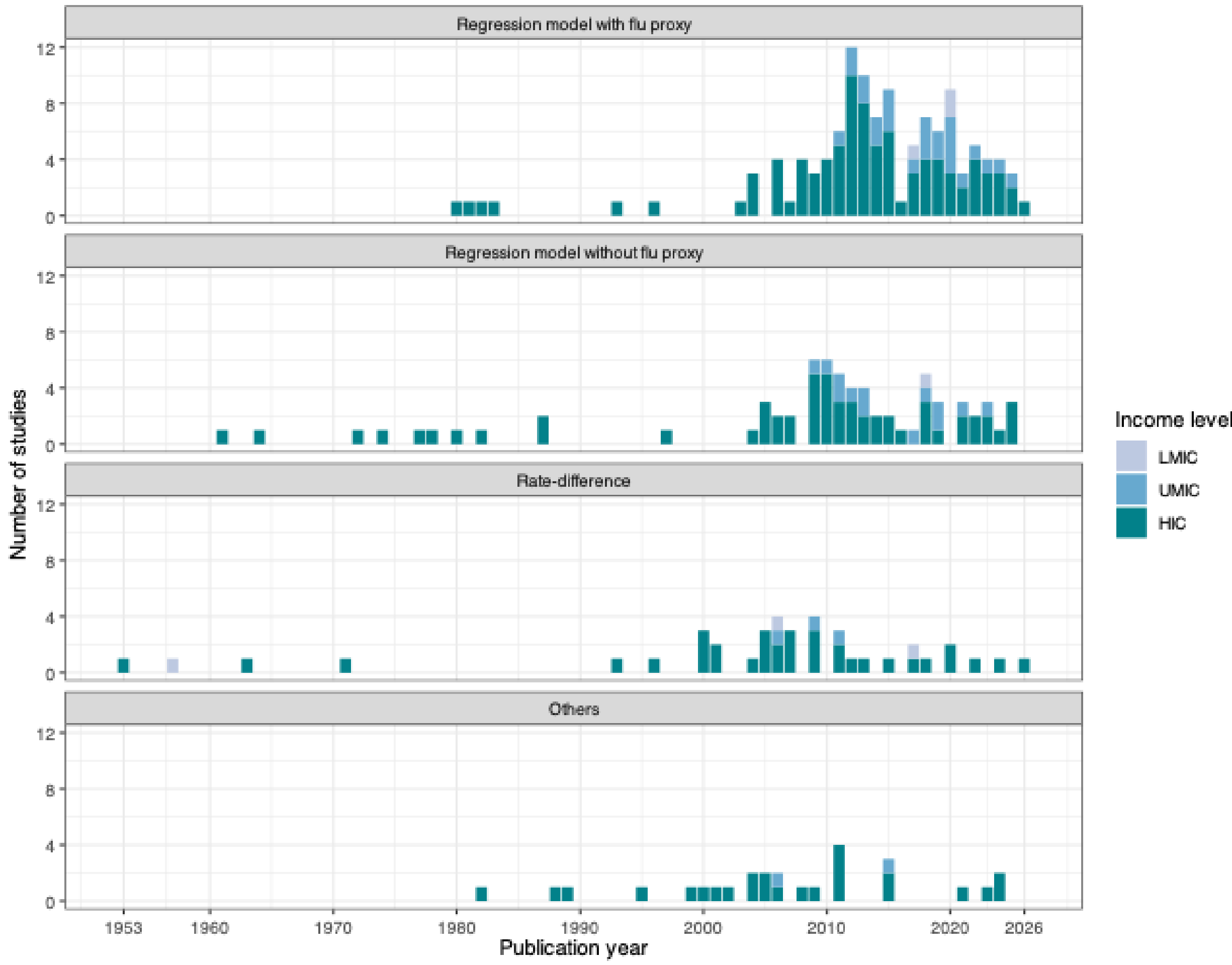

Fig S3. Number of included studies by publication year and income level, grouped by main estimation approach.

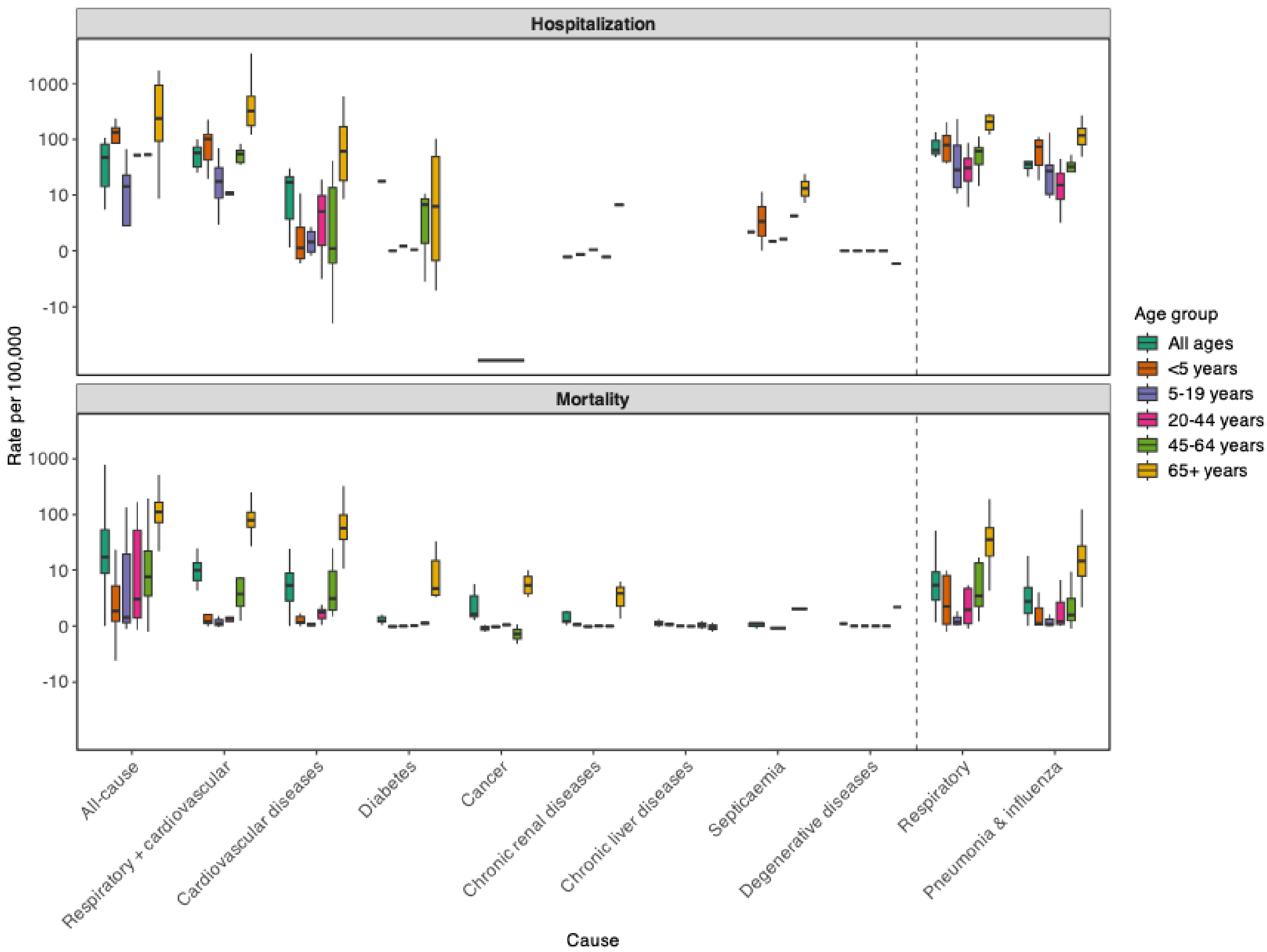

Fig S4. Summary of available influenza-associated hospitalization and mortality rate estimates by cause, outcome and age group. Each boxplot shows the distribution (median, interquartile range, and overall range) of reported rates per 100,000 population for a given cause category, with whiskers beyond them representing the full range of estimates within each age-cause-outcome stratum. Respiratory and Pneumonia & Influenza estimates were additionally extracted from included studies to serve as reference comparators. Colours indicate age groups (all ages, <5 years, 5–14 years, 20–64 years, 65–64 years, and ≥65 years), as shown in the legend. Estimates are plotted on a logarithmic scale to allow comparison across a wide range of rates.

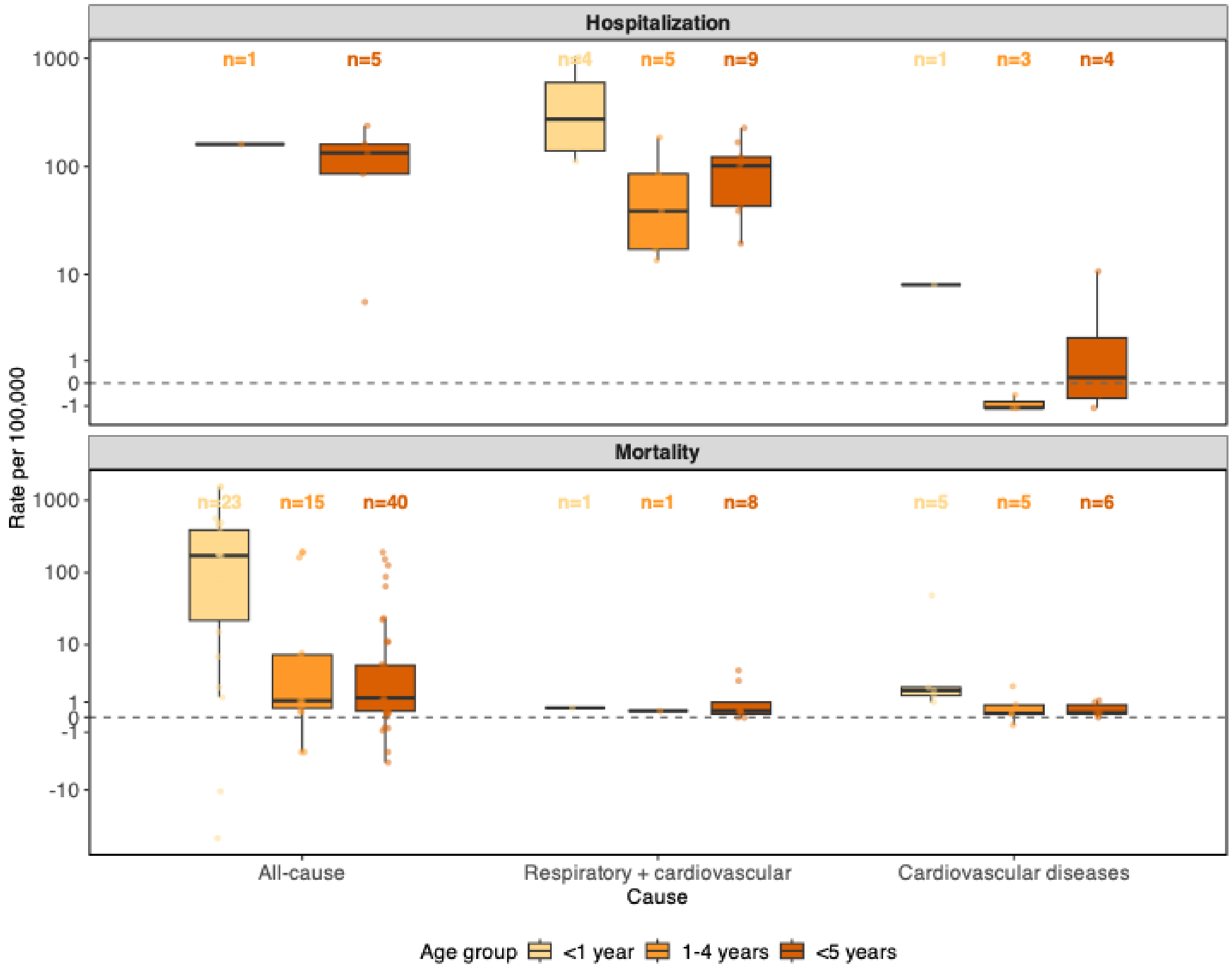

Fig S5. Influenza-associated hospitalization and mortality rates among children by narrower age groups. Box plots show the distribution of influenza-associated hospitalization (top) and mortality (bottom) rate estimates per 100,000 population for children aged <1 year, 1-4 years, and <5 years (broad group), stratified by cause category. The number of estimates is shown above each box. Boxes represent the interquartile range with the median indicated by the horizontal line; whiskers extend to the most extreme values within 1.5 times the interquartile range. Only cause categories with available data for at least one narrower age group are shown.

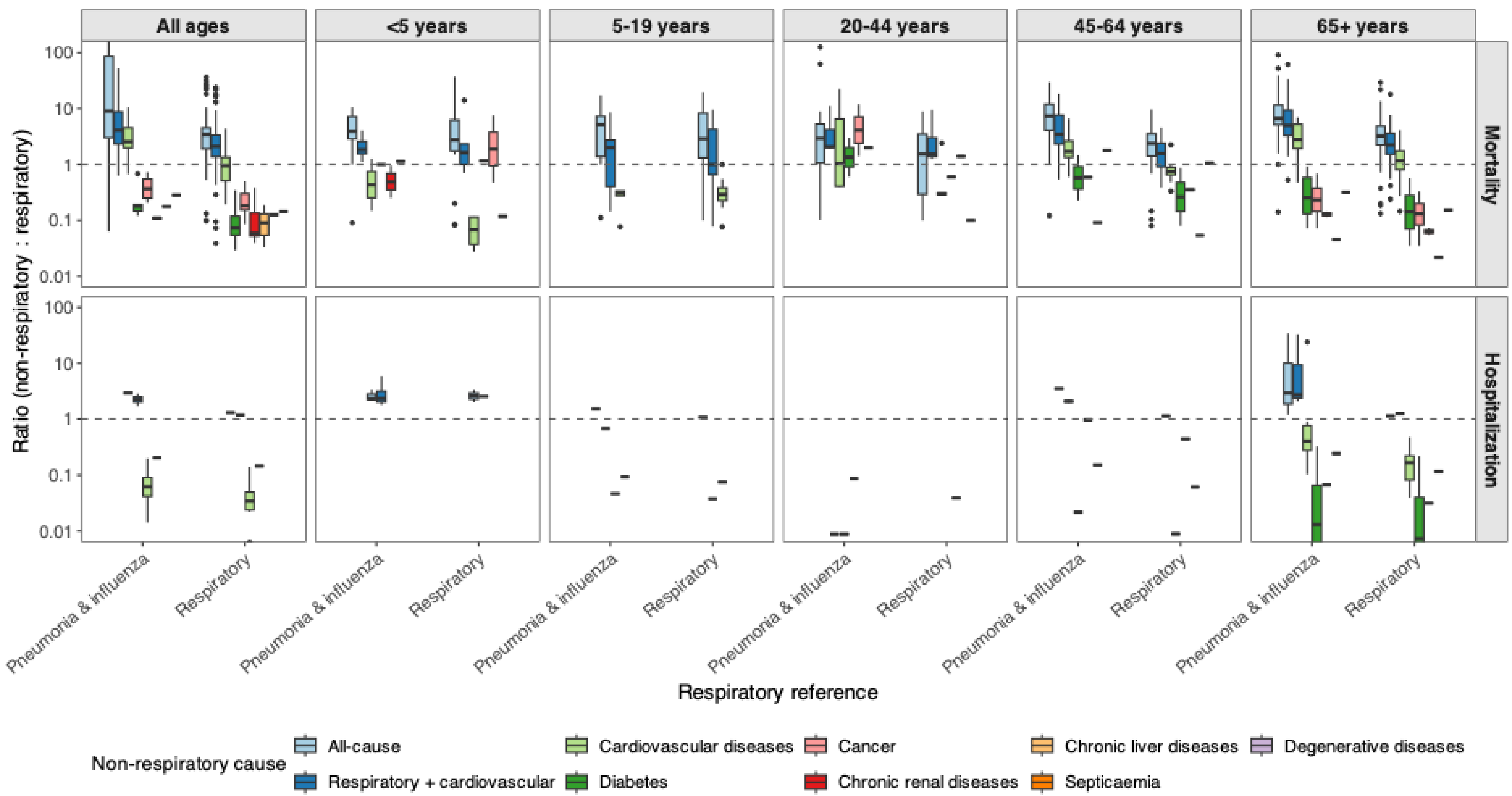

Fig S6. Ratios of non-respiratory to respiratory influenza-associated burden estimates, by age group and outcome. Each panel shows within-study ratios of non-respiratory outcomes to corresponding respiratory reference estimates (pneumonia & influenza or broad respiratory), stratified by age group and by hospitalization vs mortality; the horizontal dashed line indicates a ratio of 1 (indicating equal non-respiratory and respiratory burden).

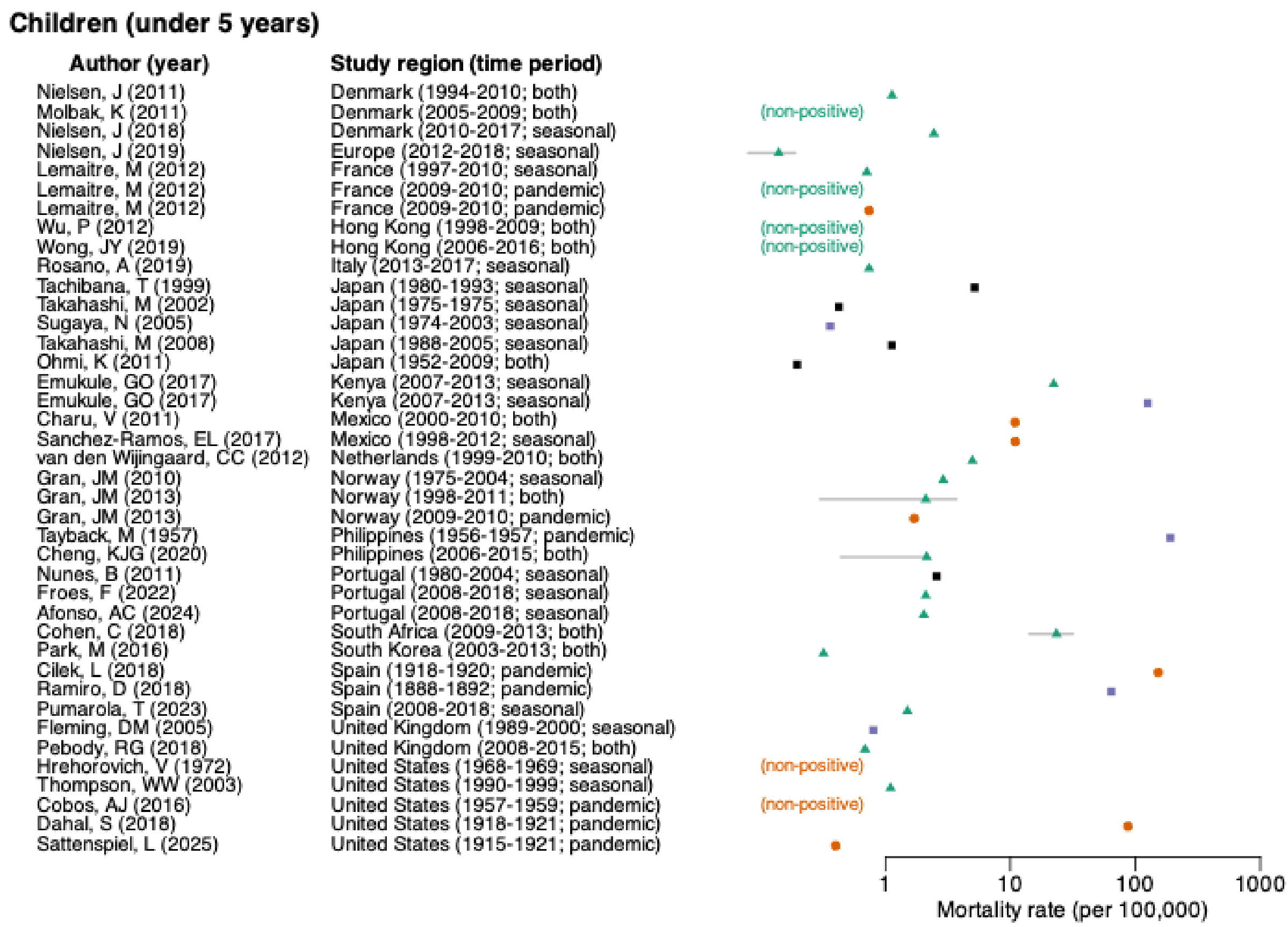

Fig S7. Forest plot of all-cause influenza-associated mortality rate estimates for children <5 years. Each point represents a study estimate of the mortality rate per 100,000 population, with horizontal lines indicating the reported confidence intervals on a logarithmic scale. Study details listed to the left include first author, publication year, study region, time period, and influenza period represented by the estimate. Colours indicate the main estimation approach, with green for regression models with an influenza activity proxy, orange for regression models without an influenza proxy (Serfling-like models), purple for rate-difference methods, and black for other methods (same for **Figs S8–S10**). Estimates labelled "non-positive" had point estimates ≤0 and are displayed at the lower plotting limit on the logarithmic x-axis.

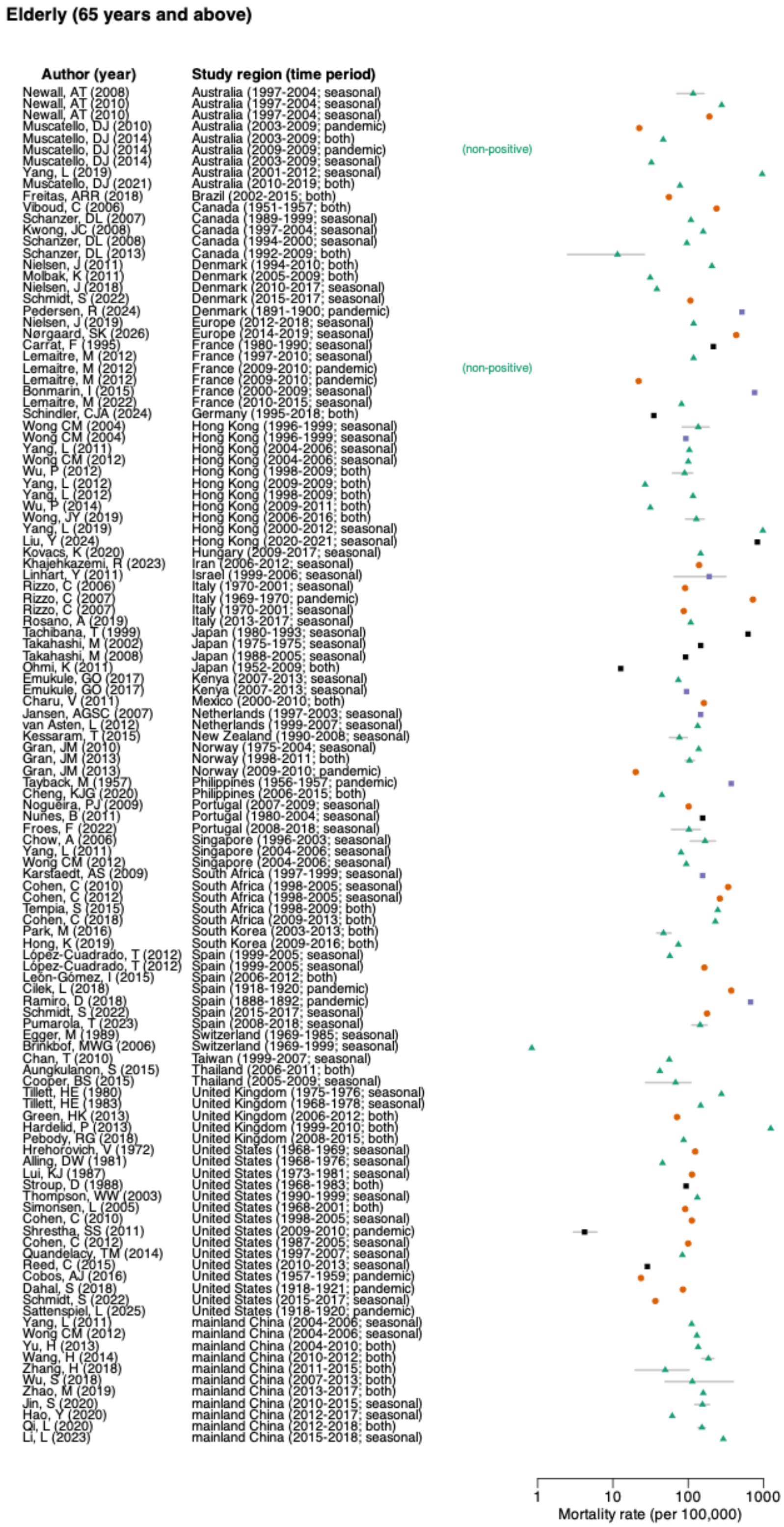

Fig S8. Forest plot of all-cause influenza-associated mortality rate estimates for elderly aged 65 years or above.

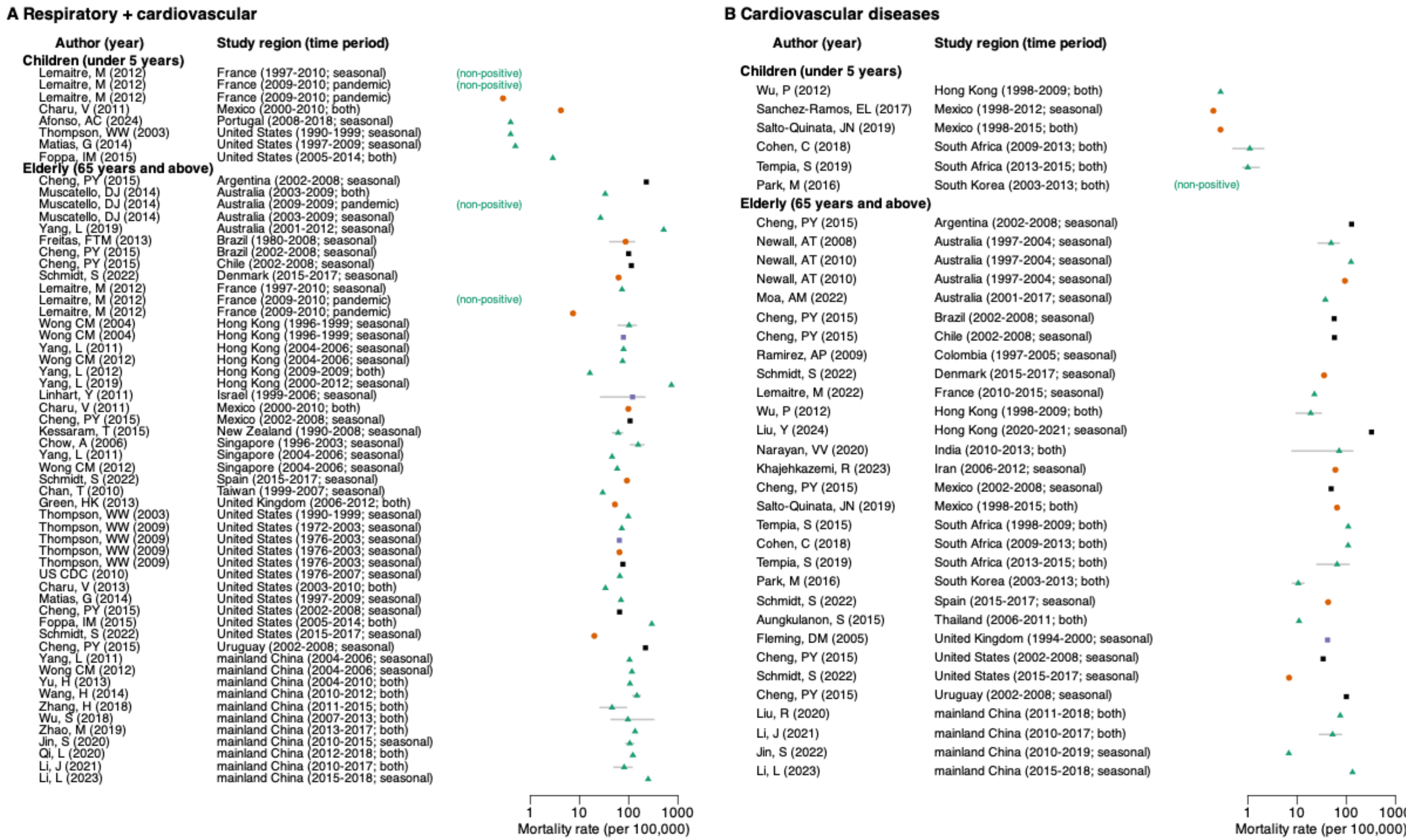

Fig S9. Forest plot of influenza-associated (A) respiratory + cardiovascular and (B) cardiovascular mortality rate estimates for children under 5 years and elderly aged 65 years or above.

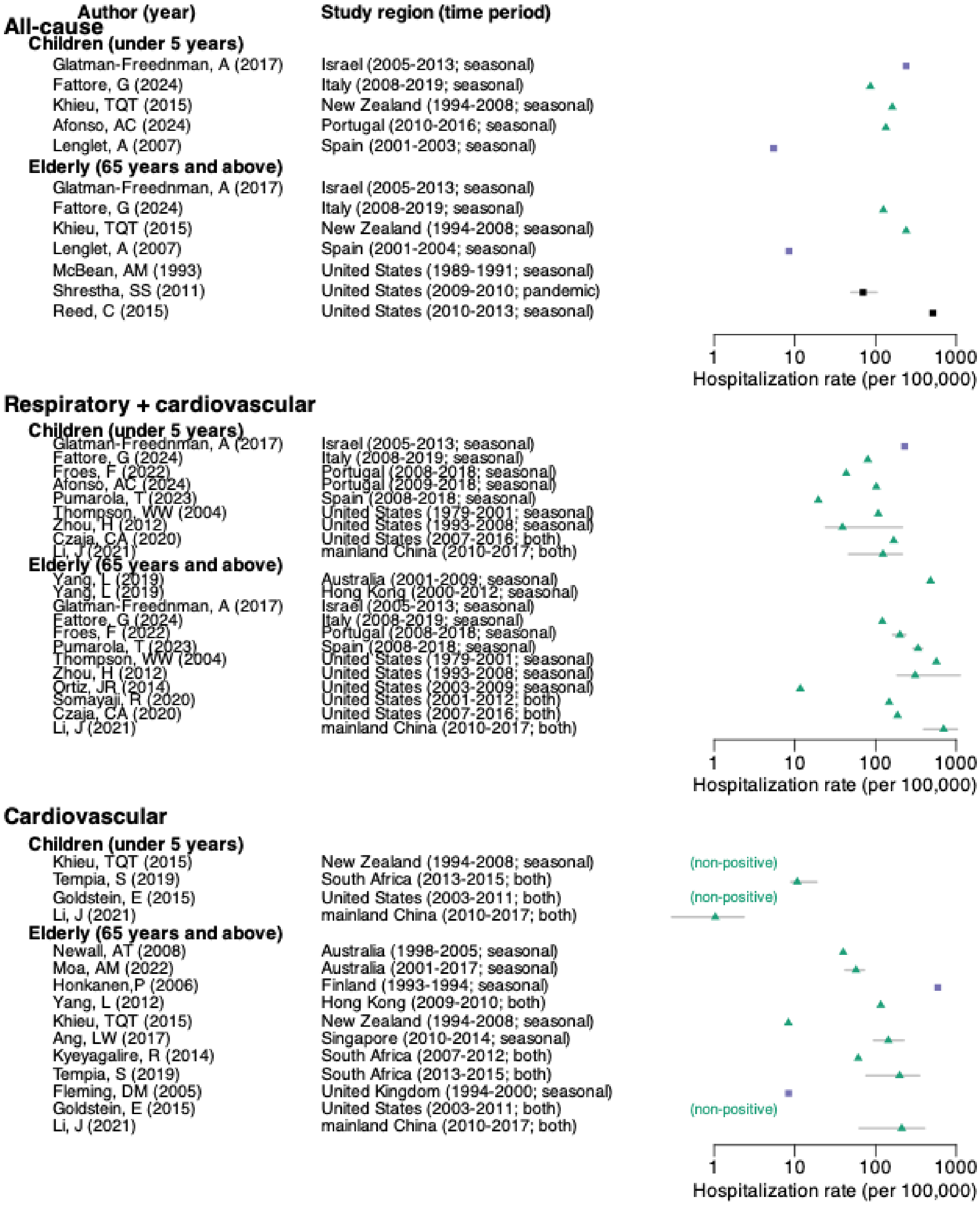

Fig S10. Forest plot for major non-respiratory influenza-associated hospitalization rate estimates for children under 5 years and elderly aged 65 years or above.

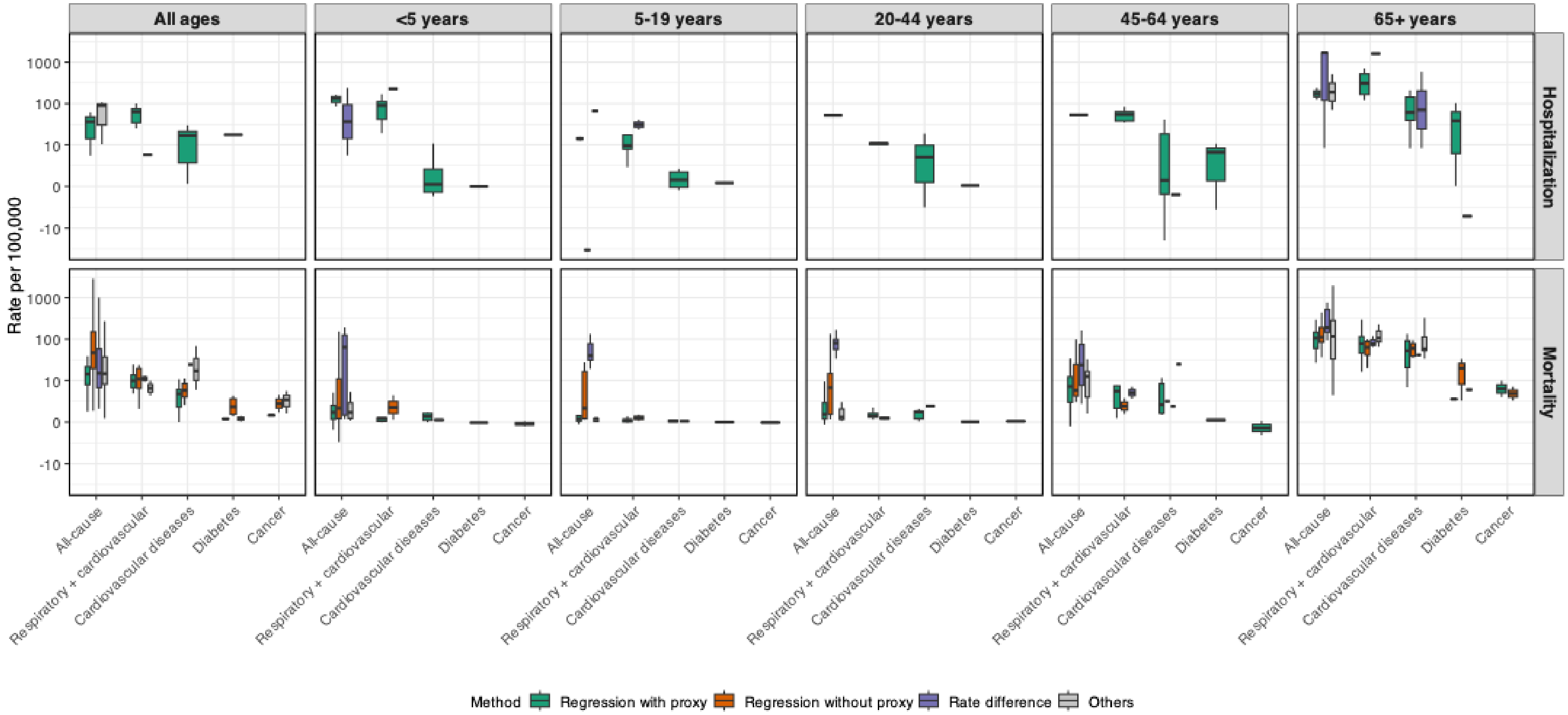

Fig S11. Summary of cause-specific influenza-associated hospitalization and mortality rate estimates by main methodological category and age group. Each panel shows boxplots of rates per 100,000 population for a given age group and outcome (hospitalization or mortality), stratified by cause category on the x-axis. Colours indicate the estimation approach (green, regression with influenza proxy; orange, regression without proxy; purple, rate-difference; grey, other methods). Boxplots summarise the distribution of estimates within each cause-method-age stratum on a logarithmic scale.

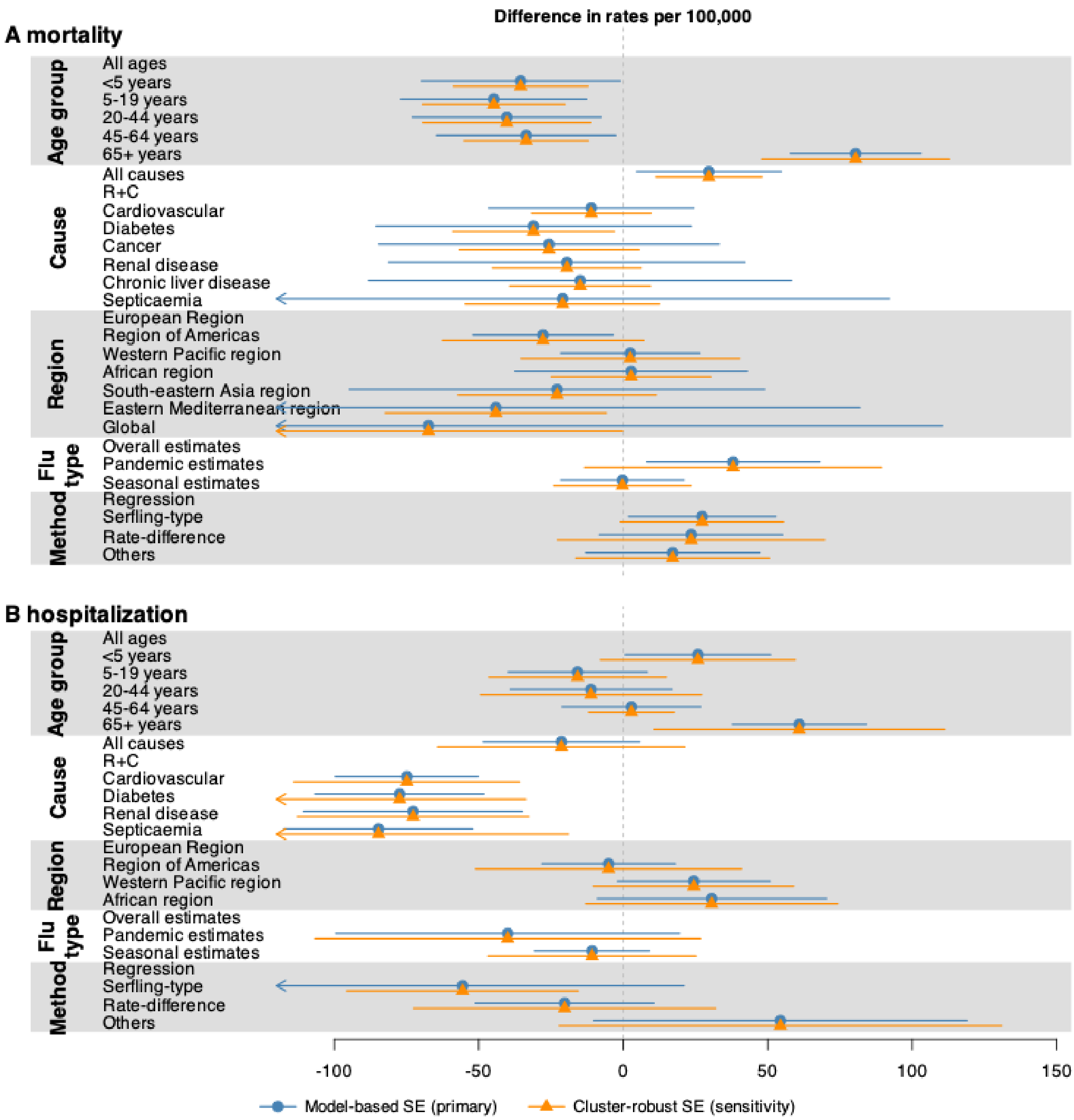

Fig S12. Sensitivity analysis comparing model-based and cluster-robust SEs in random-effects meta-regression of influenza-associated (A) mortality and (B) hospitalization rates. Blue circles with lines represent estimates and 95% CIs from the primary analysis using model-based SEs; orange triangles represent estimates using cluster-robust SEs (clustered by study). The reference categories are all ages (age group), all causes (cause), European Region (region), overall estimates (influenza type), and regression (method). Arrows indicate confidence intervals extending beyond the displayed range.

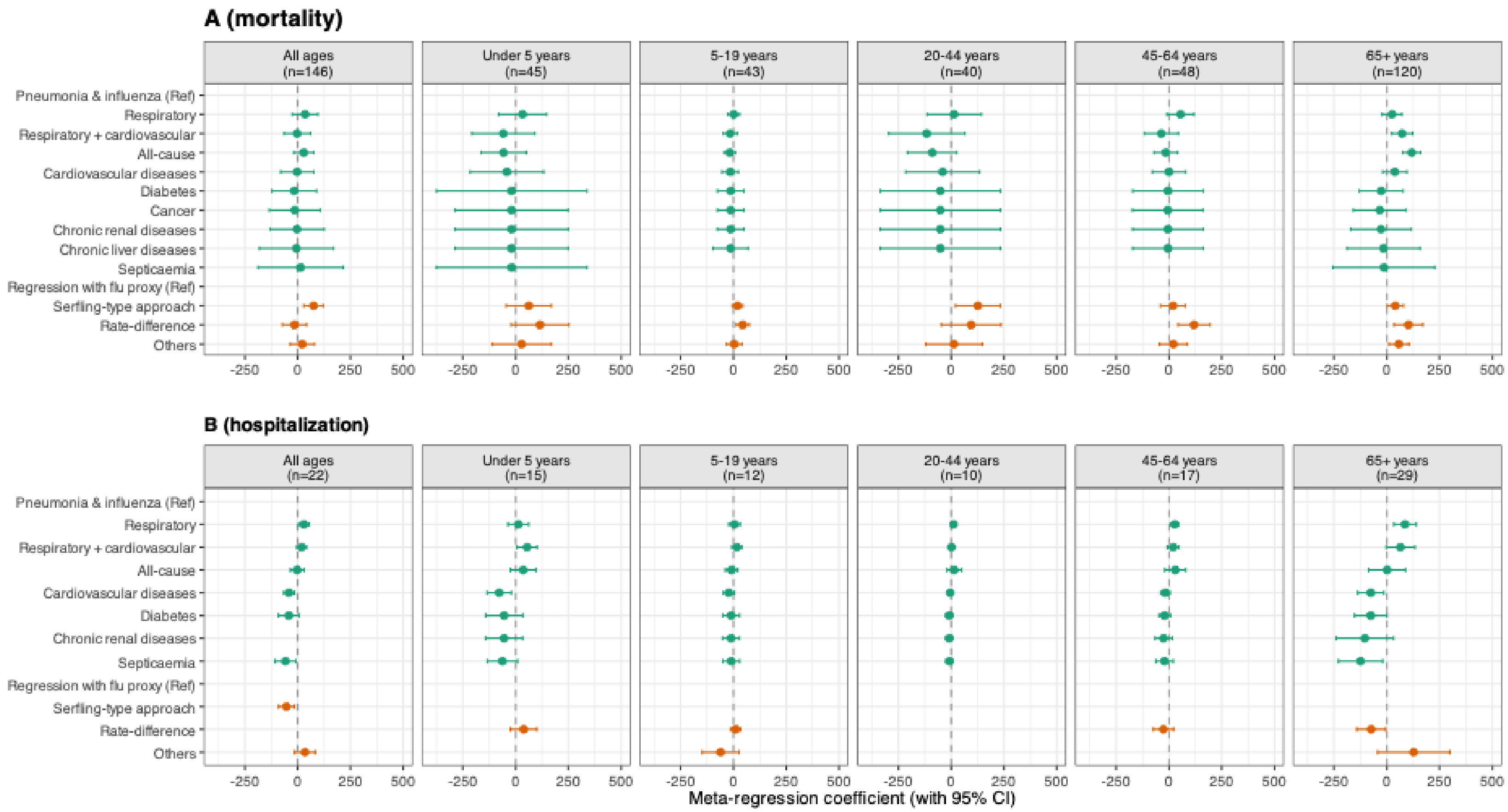

Fig S13. Meta-regression coefficients for influenza-associated mortality and hospitalization, including respiratory estimates as reference, by age group and cause category. Points and horizontal lines show meta-regression coefficients and 95% CIs for non-respiratory outcomes compared with respiratory reference categories stratified by age group. Estimates are adjusted for the same covariates as in the main meta-regression model (region and influenza type, with age accounted for by stratification). Respiratory reference estimates are included to illustrate relative differences in estimated influenza-associated burden across causes.

Supplementary References

1. Goldstein E, Viboud C, Charu V, Lipsitch M. Improving the estimation of influenza-related mortality over a seasonal baseline. *Epidemiology* 2012; **23**(6): 829-38.
2. Wu P, Goldstein E, Ho LM, et al. Excess mortality associated with influenza A and B virus in Hong Kong, 1998-2009. *J Infect Dis* 2012; **206**(12): 1862-71.
3. Wong JY, Cheung CM, Bond HS, et al. Estimating excess septicaemia mortality and hospitalisation burden associated with influenza in Hong Kong, 1998 to 2019. *Epidemiol Infect* 2022; **150**: e101.
4. WHO. Pandemic influenza severity assessment (PISA): a WHO guide to assess the severity of influenza in seasonal epidemics and pandemics, second edition, 2025.
5. Reed C, Chaves SS, Daily Kirley P, et al. Estimating influenza disease burden from population-based surveillance data in the United States. *PLoS One* 2015; **10**(3): e0118369.
6. Higgins JPT, Morgan RL, Rooney AA, et al. A tool to assess risk of bias in non-randomized follow-up studies of exposure effects (ROBINS-E). *Environ Int* 2024; **186**: 108602.
7. WHO. Countries/areas by WHO region. https://apps.who.int/violence-info/Countries%20and%20areas%20by%20WHO%20region%20-%2012bfe12.pdf (accessed Dec 19 2025).
8. Bank W. World Bank country classifications by income level for 2024-2025. July 1, 2024 2024. https://blogs.worldbank.org/en/opendata/world-bank-country-classifications-by-income-level-for-2024-2025 (accessed Dec 19 2025).
9. Li L, Wong JY, Wu P, et al. Heterogeneity in Estimates of the Impact of Influenza on Population Mortality: A Systematic Review. *Am J Epidemiol* 2018; **187**(2): 378-88.
10. Charu V, Chowell G, Palacio Mejia LS, et al. Mortality burden of the A/H1N1 pandemic in Mexico: a comparison of deaths and years of life lost to seasonal influenza. *Clin Infect Dis* 2011; **53**(10): 985-93.
11. Poehling KA, Edwards KM, Weinberg GA, et al. The underrecognized burden of influenza in young children. *N Engl J Med* 2006; **355**(1): 31-40.
12. Warren-Gash C, Hayward AC, Hemingway H, et al. Influenza infection and risk of acute myocardial infarction in England and Wales: a CALIBER self-controlled case series study. *J Infect Dis* 2012; **206**(11): 1652-9.
13. Alling DW, Blackwelder WC, Stuart-Harris CH. A study of excess mortality during influenza epidemics in the United States, 1968-1976. *Am J Epidemiol* 1981; **113**(1): 30-43.
14. Andreasen V, Simonsen L. The perils of using annual all-cause mortality data to estimate pandemic influenza burden. *Vaccine* 2011; **29 Suppl 2**: B49-55.
15. Ang LW, Yap J, Lee V, et al. Influenza-Associated Hospitalizations for Cardiovascular Diseases in the Tropics. *Am J Epidemiol* 2017; **186**(2): 202-9.
16. Ansart S, Pelat C, Boelle PY, Carrat F, Flahault A, Valleron AJ. Mortality burden of the 1918-1919 influenza pandemic in Europe. *Influenza Other Respir Viruses* 2009; **3**(3): 99-106.
17. Aungkulanon S, Cheng PY, Kusreesakul K, et al. Influenza-associated mortality in Thailand, 2006-2011. *Influenza Other Respir Viruses* 2015; **9**(6): 298-304.

18. Azziz-Baumgartner E, Cabrera AM, Cheng PY, et al. Incidence of influenza-associated mortality and hospitalizations in Argentina during 2002-2009. *Influenza Other Respir Viruses* 2013; **7**(5): 710-7.
19. Bertolani A, Fattore G, Pregliasco F. The hospitalization burden of influenza: just the tip of the iceberg? *Global & Regional Health Technology Assessment* 2018; **2018**: 2284240318777148.
20. Blackburn R, Zhao H, Pebody R, Hayward A, Warren-Gash C. Laboratory-Confirmed Respiratory Infections as Predictors of Hospital Admission for Myocardial Infarction and Stroke: Time-Series Analysis of English Data for 2004-2015. *Clin Infect Dis* 2018; **67**(1): 8-17.
21. Bonmarin I, Belchior E, Lévy-Bruhl D. Impact of influenza vaccination on mortality in the French elderly population during the 2000-2009 period. *Vaccine* 2015; **33**(9): 1099-101.
22. Brinkhof MW, Spoerri A, Birrer A, Hagman R, Koch D, Zwahlen M. Influenza-attributable mortality among the elderly in Switzerland. *Swiss Med Wkly* 2006; **136**(19-20): 302-9.
23. Caldas Afonso A, Gouveia C, Januário G, et al. Uncovering the burden of Influenza in children in Portugal, 2008-2018. *BMC Infect Dis* 2024; **24**(1): 100.
24. Carrat F, Valleron AJ. Influenza mortality among the elderly in France, 1980-90: how many deaths may have been avoided through vaccination? *J Epidemiol Community Health* 1995; **49**(4): 419-25.
25. Chan TC, Hsiao CK, Lee CC, et al. The impact of matching vaccine strains and post-SARS public health efforts on reducing influenza-associated mortality among the elderly. *PLoS One* 2010; **5**(6): e11317.
26. Chandra S, Christensen J. Tracking Pandemic Severity Using Data on the Age Structure of Mortality: Lessons From the 1918 Influenza Pandemic in Michigan. *Am J Public Health* 2021; **111**(S2): S149-s55.
27. Charu V, Simonsen L, Lustig R, Steiner C, Viboud C. Mortality burden of the 2009-10 influenza pandemic in the United States: improving the timeliness of influenza severity estimates using inpatient mortality records. *Influenza Other Respir Viruses* 2013; **7**(5): 863-71.
28. Chaves SS, Nealon J, Burkart KG, et al. Global, regional and national estimates of influenza-attributable ischemic heart disease mortality. *EClinicalMedicine* 2023; **55**: 101740.
29. Cheng PY, Palekar R, Azziz-Baumgartner E, et al. Burden of influenza-associated deaths in the Americas, 2002-2008. *Influenza Other Respir Viruses* 2015; **9 Suppl 1**(Suppl 1): 13-21.
30. Cheng KJG, Rivera AS, Lam HY, et al. Influenza-associated excess mortality in the Philippines, 2006-2015. *PLoS One* 2020; **15**(6): e0234715.
31. Choi K, Thacker SB. Mortality during influenza epidemics in the United States, 1967-1978. *Am J Public Health* 1982; **72**(11): 1280-3.
32. Chow A, Ma S, Ling AE, Chew SK. Influenza-associated deaths in tropical Singapore. *Emerg Infect Dis* 2006; **12**(1): 114-21.
33. Chowell G, Viboud C, Simonsen L, et al. The 1918-1920 influenza pandemic in Peru. *Vaccine* 2011; **29 Suppl 2**(Suppl 2): B21-6.

34. Chowell G, Erkoreka A, Viboud C, Echeverri-Dávila B. Spatial-temporal excess mortality patterns of the 1918-1919 influenza pandemic in Spain. *BMC Infect Dis* 2014; **14**: 371.
35. Cilek L, Chowell G, Ramiro Fariñas D. Age-Specific Excess Mortality Patterns During the 1918-1920 Influenza Pandemic in Madrid, Spain. *Am J Epidemiol* 2018; **187**(12): 2511-23.
36. Clay K, Lewis J, Severnini E. What explains cross-city variation in mortality during the 1918 influenza pandemic? Evidence from 438 U.S. cities. *Econ Hum Biol* 2019; **35**: 42-50.
37. Cobos AJ, Nelson CG, Jehn M, Viboud C, Chowell G. Mortality and transmissibility patterns of the 1957 influenza pandemic in Maricopa County, Arizona. *BMC Infect Dis* 2016; **16**(1): 405.
38. Cohen C, Simonsen L, Kang JW, et al. Elevated influenza-related excess mortality in South African elderly individuals, 1998-2005. *Clin Infect Dis* 2010; **51**(12): 1362-9.
39. Cohen C, Simonsen L, Sample J, et al. Influenza-related mortality among adults aged 25-54 years with AIDS in South Africa and the United States of America. *Clin Infect Dis* 2012; **55**(7): 996-1003.
40. Cohen C, Walaza S, Treurnicht FK, et al. In- and Out-of-hospital Mortality Associated with Seasonal and Pandemic Influenza and Respiratory Syncytial Virus in South Africa, 2009-2013. *Clin Infect Dis* 2018; **66**(1): 95-103.
41. Collins SD, Lehmann J. Excess deaths from influenza and pneumonia and from important chronic diseases during epidemic periods, 1918-51. *Public Health Monogr* 1953; **10**: 1-21.
42. Comas-García A, García-Sepúlveda CA, Méndez-de Lira JJ, Aranda-Romo S, Hernández-Salinas AE, Noyola DE. Mortality attributable to pandemic influenza A (H1N1) 2009 in San Luis Potosí, Mexico. *Influenza Other Respir Viruses* 2011; **5**(2): 76-82.
43. Cooper BS, Kotirum S, Kulpeng W, et al. Mortality attributable to seasonal influenza A and B infections in Thailand, 2005-2009: a longitudinal study. *Am J Epidemiol* 2015; **181**(11): 898-907.
44. Cox B, Wuillaume F, Van Oyen H, Maes S. Monitoring of all-cause mortality in Belgium (Be-MOMO): a new and automated system for the early detection and quantification of the mortality impact of public health events. *Int J Public Health* 2010; **55**(4): 251-9.
45. Czaja CA, Miller L, Colborn K, et al. State-level estimates of excess hospitalizations and deaths associated with influenza. *Influenza Other Respir Viruses* 2020; **14**(2): 111-21.
46. Dahal S, Jenner M, Dinh L, Mizumoto K, Viboud C, Chowell G. Excess mortality patterns during 1918-1921 influenza pandemic in the state of Arizona, USA. *Ann Epidemiol* 2018; **28**(5): 273-80.
47. Dawood FS, Iuliano AD, Reed C, et al. Estimated global mortality associated with the first 12 months of 2009 pandemic influenza A H1N1 virus circulation: a modelling study. *Lancet Infect Dis* 2012; **12**(9): 687-95.
48. Deng X, Liu J, Wang M, et al. Changes in influenza-associated excess mortality in China between 2012-2019 and 2020-2021: a population-based statistical modelling study. *Infect Dis Poverty* 2025; **14**(1): 52.

49. Dushoff J, Plotkin JB, Viboud C, Earn DJ, Simonsen L. Mortality due to influenza in the United States--an annualized regression approach using multiple-cause mortality data. *Am J Epidemiol* 2006; **163**(2): 181-7.
50. Egger M, Jennings S, Spuhler T, Zimmermann HP, Paccaud F, Somaini B. [Mortality in influenza epidemics in Switzerland 1969-1985]. *Schweiz Med Wochenschr* 1989; **119**(13-14): 434-9.
51. Eickhoff TC, Sherman IL, Serfling RE. Observations on excess mortality associated with epidemic influenza. *Jama* 1961; **176**: 776-82.
52. Emukule GO, Spreeuwenberg P, Chaves SS, et al. Estimating influenza and respiratory syncytial virus-associated mortality in Western Kenya using health and demographic surveillance system data, 2007-2013. *PLoS One* 2017; **12**(7): e0180890.
53. Fattore G, Pongiglione B, Vezzosi L. Excess hospitalizations and in-hospital mortality associated with seasonal influenza in Italy: a 11-year retrospective study. *BMC Infect Dis* 2024; **24**(1): 227.
54. Faust JS, Lin Z, Del Rio C. Comparison of Estimated Excess Deaths in New York City During the COVID-19 and 1918 Influenza Pandemics. *JAMA Netw Open* 2020; **3**(8): e2017527.
55. Feng L, Shay DK, Jiang Y, et al. Influenza-associated mortality in temperate and subtropical Chinese cities, 2003-2008. *Bull World Health Organ* 2012; **90**(4): 279-88b.
56. Fireman B, Lee J, Lewis N, Bembom O, van der Laan M, Baxter R. Influenza vaccination and mortality: differentiating vaccine effects from bias. *Am J Epidemiol* 2009; **170**(5): 650-6.
57. Fleming DM. The impact of three influenza epidemics on primary care in England and Wales. *Pharmacoeconomics* 1996; **9 Suppl 3**: 38-45; discussion 50-3.
58. Fleming DM. The contribution of influenza to combined acute respiratory infections, hospital admissions, and deaths in winter. *Commun Dis Public Health* 2000; **3**(1): 32-8.
59. Fleming DM, Pannell RS, Cross KW. Mortality in children from influenza and respiratory syncytial virus. *J Epidemiol Community Health* 2005; **59**(7): 586-90.
60. Fleming DM, Cross KW, Pannell RS. Influenza and its relationship to circulatory disorders. *Epidemiol Infect* 2005; **133**(2): 255-62.
61. Foppa IM, Hossain MM. Revised estimates of influenza-associated excess mortality, United States, 1995 through 2005. *Emerg Themes Epidemiol* 2008; **5**: 26.
62. Foppa IM, Cheng PY, Reynolds SB, et al. Deaths averted by influenza vaccination in the U.S. during the seasons 2005/06 through 2013/14. *Vaccine* 2015; **33**(26): 3003-9.
63. Freitas FT, Souza LR, Azziz-Baumgartner E, et al. Influenza-associated excess mortality in southern Brazil, 1980-2008. *Epidemiol Infect* 2013; **141**(8): 1731-40.
64. Freitas ARR, Donalisio MR. Excess of Mortality in Adults and Elderly and Circulation of Subtypes of Influenza Virus in Southern Brazil. *Front Immunol* 2017; **8**: 1903.
65. Froes F, Carmo M, Lopes H, et al. Excess hospitalizations and mortality associated with seasonal influenza in Portugal, 2008-2018. *BMC Infect Dis* 2022; **22**(1): 726.
66. Gavrilova NS, Gavrilov LA. Patterns of mortality during pandemic: An example of Spanish flu pandemic of 1918. *Popul Econ* 2020; **4**(2): 56-64.
67. Glatman-Freedman A, Kaufman Z, Stein Y, et al. Influenza Season Hospitalization Trends in Israel: A Multi-Year Comparative Analysis 2005/2006 Through 2012/2013. *J Hosp Med* 2017; **12**(9): 710-6.

68. Goldstein E, Greene SK, Olson DR, Hanage WP, Lipsitch M. Estimating the hospitalization burden associated with influenza and respiratory syncytial virus in New York City, 2003-2011. *Influenza Other Respir Viruses* 2015; **9**(5): 225-33.
69. Goldstein E. Mortality associated with Omicron and influenza infections in France before and during the COVID-19 pandemic. *Epidemiol Infect* 2023; **151**: e148.
70. Gómez-Gómez A, Sánchez-Ramos EL, Noyola DE. Diabetes is a major cause of influenza-associated mortality in Mexico. *Rev Epidemiol Sante Publique* 2021; **69**(4): 205-13.
71. Gran JM, Iversen B, Hungnes O, Aalen OO. Estimating influenza-related excess mortality and reproduction numbers for seasonal influenza in Norway, 1975-2004. *Epidemiol Infect* 2010; **138**(11): 1559-68.
72. Gran JM, Kacelnik O, Grjibovski AM, Aavitsland P, Iversen BG. Counting pandemic deaths: comparing reported numbers of deaths from influenza A(H1N1)pdm09 with estimated excess mortality. *Influenza Other Respir Viruses* 2013; **7**(6): 1370-9.
73. Green HK, Andrews N, Fleming D, Zambon M, Pebody R. Mortality attributable to influenza in England and Wales prior to, during and after the 2009 pandemic. *PLoS One* 2013; **8**(12): e79360.
74. Green HK, Zhao H, Boddington NL, et al. Detection of varying influenza circulation within England in 2012/13: informing antiviral prescription and public health response. *J Public Health (Oxf)* 2015; **37**(2): 295-304.
75. Hai JJ, Liu D, Leung K, et al. Impacts of viral respiratory infections on segments of fatal out-of-hospital cardiac arrests. *Postgrad Med J* 2025; **101**(1201): 1156-65.
76. Hanzlíková H, Plavcová E, Kyselý J, Kynčl J, Malý M, Urban A. Links between influenza epidemics, weather characteristics and all-cause mortality in the Czech Republic, 1982-2020. *BMC Public Health* 2025; **26**(1): 189.
77. Hao Y, Huang L, Liu X, Chen Y, Li J, Wang X. Influenza-attributable years of life lost in older adults in a subtropical city in China, 2012-2017: A modeling study based on a competing risks approach. *Int J Infect Dis* 2020; **97**: 354-9.
78. Hardelid P, Andrews N, Pebody R. Excess mortality monitoring in England and Wales during the influenza A(H1N1) 2009 pandemic. *Epidemiol Infect* 2011; **139**(9): 1431-9.
79. Hardelid P, Pebody R, Andrews N. Mortality caused by influenza and respiratory syncytial virus by age group in England and Wales 1999-2010. *Influenza Other Respir Viruses* 2013; **7**(1): 35-45.
80. Heudorf U, Kowall B. All-cause excess mortality in Germany peaked during the late-2022 influenza period, exceeding peaks during SARS-CoV-2 waves (2020-2023). *PLoS One* 2026; **21**(6): e0335982.
81. Hong K, Sohn S, Chun BC. Estimating Influenza-associated Mortality in Korea: The 2009-2016 Seasons. *J Prev Med Public Health* 2019; **52**(5): 308-15.
82. Honkanen P, Läärä E, Pyhälä R, Kivelä SL, Helena Mäkelä P. Comparison of two vaccination programmes in preventing influenza-related hospitalization among the elderly during two consecutive seasons. *Scand J Infect Dis* 2006; **38**(6-7): 506-11.
83. Housworth J, Langmuir AD. Excess mortality from epidemic influenza, 1957-1966. *Am J Epidemiol* 1974; **100**(1): 40-8.
84. Hrehorovich V, Dyal WW, Schrack WD, Jr. Influenza epidemic in Pennsylvania. *Health Serv Rep* 1972; **87**(9): 835-44.

85. Bock C, Kuhn D, Schneider S, Zoellner I. Seasonal waves of influenza and cause-specific mortality in Germany. *Central European Journal of Medicine* 2012; **7**: 450-6.
86. Imaz MS, Eimann M, Poyard E, Savy V. [Influenza associated excess mortality in Argentina: 1992-2002]. *Rev Chilena Infectol* 2006; **23**(4): 297-306.
87. Jang H, Cho J, Cho SK, et al. All-Cause and Cause-Specific Mortality Attributable to Seasonal Influenza: A Nationwide Matched Cohort Study. *J Korean Med Sci* 2023; **38**(25): e188.
88. Jansen AG, Sanders EA, Hoes AW, van Loon AM, Hak E. Influenza- and respiratory syncytial virus-associated mortality and hospitalisations. *Eur Respir J* 2007; **30**(6): 1158-66.
89. Jin S, Li J, Cai R, et al. Age- and sex-specific excess mortality associated with influenza in Shanghai, China, 2010-2015. *Int J Infect Dis* 2020; **98**: 382-9.
90. Jin S, Jiang C, Xia T, et al. Age-dependent and sex-dependent differences in mortality from influenza-associated cardiovascular diseases among older adults in Shanghai, China: a population-based study. *BMJ Open* 2022; **12**(9): e061068.
91. Karstaedt AS, Hopley M, Wong M, Crewe-Brown HH, Tasset-Tisseau A. Influenza- and respiratory syncytial virus-associated adult mortality in Soweto. *S Afr Med J* 2009; **99**(10): 750-4.
92. Kessaram T, Stanley J, Baker MG. Estimating influenza-associated mortality in New Zealand from 1990 to 2008. *Influenza Other Respir Viruses* 2015; **9**(1): 14-9.
93. Khajehkazemi R, Baneshi MR, Iuliano AD, et al. Estimated mortality due to seasonal influenza in southeast of Iran, 2006/2007 to 2011/2012 influenza seasons. *Influenza Other Respir Viruses* 2023; **17**(1): e13061.
94. Khieu TQ, Pierse N, Telfar-Barnard LF, Huang QS, Baker MG. Estimating the contribution of influenza to hospitalisations in New Zealand from 1994 to 2008. *Vaccine* 2015; **33**(33): 4087-92.
95. King JC, Ajao A, Lichenstein R, Magder LS. Surge in Hospitalizations Associated With Mechanical Ventilator Use During Influenza Outbreaks. *Disaster Med Public Health Prep* 2014; **8**(2): 136-42.
96. Kovács K, Pakot L. [Influenza-associated mortality in Hungary between 2009/2010 and 2016/2017]. *Orv Hetil* 2020; **161**(23): 962-70.
97. Pedersen RK, Ingholt MM, Van Wijhe M, Andreasen V, Simonsen L. Identifying signature features of epidemic diseases from 19th century all-cause mortality data. *Am J Epidemiol* 2025; **194**(2): 460-8.
98. Kuo HW, Schmid D, Liu YL, Lachner P, Allerberger F. Influenza-related excess mortality, Austria 2001 till 2009. *Wien Klin Wochenschr* 2011; **123**(19-20): 593-8.
99. Kwok KO, Riley S, Perera R, et al. Relative incidence and individual-level severity of seasonal influenza A H3N2 compared with 2009 pandemic H1N1. *BMC Infect Dis* 2017; **17**(1): 337.
100. Kwong JC, Stukel TA, Lim J, et al. The effect of universal influenza immunization on mortality and health care use. *PLoS Med* 2008; **5**(10): e211.
101. Kyeyagalire R, Tempia S, Cohen AL, et al. Hospitalizations associated with influenza and respiratory syncytial virus among patients attending a network of private hospitals in South Africa, 2007-2012. *BMC Infect Dis* 2014; **14**: 694.
102. Kyncl J, Prochazka B, Goddard NL, et al. A study of excess mortality during influenza epidemics in the Czech Republic, 1982-2000. *Eur J Epidemiol* 2005; **20**(4): 365-71.

103. Kyncl J, Brabec M, Maly M, Simka V, Urban A. Influenza-Related Deaths in the Czech Republic Over 21 Seasons. *Influenza Other Respir Viruses* 2025; **19**(3): e70072.
104. López-Cuadrado T, de Mateo S, Jiménez-Jorge S, Savulescu C, Larrauri A. Influenza-related mortality in Spain, 1999-2005. *Gac Sanit* 2012; **26**(4): 325-9.
105. Langmuir AD, Henderson DA, Serfling RE. THE EPIDEMIOLOGICAL BASIS FOR THE CONTROL OF INFLUENZA. *Am J Public Health Nations Health* 1964; **54**(4): 563-71.
106. Ledberg A. Mortality of the COVID-19 Outbreak in Sweden in Relation to Previous Severe Disease Outbreaks. *Front Public Health* 2021; **9**: 579948.
107. León-Gómez I, Delgado-Sanz C, Jiménez-Jorge S, et al. [Excess mortality associated with influenza in Spain in winter 2012]. *Gac Sanit* 2015; **29**(4): 258-65.
108. Lee VJ, Chen MI, Chan SP, et al. Influenza pandemics in Singapore, a tropical, globally connected city. *Emerg Infect Dis* 2007; **13**(7): 1052-7.
109. Lee VJ, Yap J, Ong JB, et al. Influenza excess mortality from 1950-2000 in tropical Singapore. *PLoS One* 2009; **4**(12): e8096.
110. Lemaitre M, Carrat F, Rey G, Miller M, Simonsen L, Viboud C. Mortality burden of the 2009 A/H1N1 influenza pandemic in France: comparison to seasonal influenza and the A/H3N2 pandemic. *PLoS One* 2012; **7**(9): e45051.
111. Lemaitre M, Fouad F, Carrat F, et al. Estimating the burden of influenza-related and associated hospitalizations and deaths in France: An eight-season data study, 2010-2018. *Influenza Other Respir Viruses* 2022; **16**(4): 717-25.
112. Lenglet AD, Hernando V, Rodrigo P, Larrauri A, Donado JD, de Mateo S. Impact of flu on hospital admissions during 4 flu seasons in Spain, 2000-2004. *BMC Public Health* 2007; **7**: 197.
113. Levi M, Cipriani F, Balzi D. All-cause mortality during the influenza season 2019-2020: comparison with previous influenza seasons in the territory of the local health authority "Central Tuscany", Italy. *Ann Ig* 2022; **34**(2): 150-5.
114. Li CK, Choi BC, Wong TW. Influenza-related deaths and hospitalizations in Hong Kong: a subtropical area. *Public Health* 2006; **120**(6): 517-24.
115. Li J, Wang C, Ruan L, et al. Development of influenza-associated disease burden pyramid in Shanghai, China, 2010-2017: a Bayesian modelling study. *BMJ Open* 2021; **11**(9): e047526.
116. Li L, Yan ZL, Luo L, et al. Influenza-Associated Excess Mortality by Age, Sex, and Subtype/Lineage: Population-Based Time-Series Study With a Distributed-Lag Nonlinear Model. *JMIR Public Health Surveill* 2023; **9**: e42530.
117. Linhart Y, Shohat T, Bromberg M, Mendelson E, Dictiar R, Green MS. Excess mortality from seasonal influenza is negligible below the age of 50 in Israel: implications for vaccine policy. *Infection* 2011; **39**(5): 399-404.
118. Liu XX, Qin G, Li X, et al. Excess mortality associated with influenza after the 2009 H1N1 pandemic in a subtropical city in China, 2010-2015. *Int J Infect Dis* 2017; **57**: 54-60.
119. Liu R, Liu X, Yang P, et al. Influenza-associated cardiovascular mortality in older adults in Beijing, China: a population-based time-series study. *BMJ Open* 2020; **10**(11): e042487.
120. Liu Y, Jingyu X, Cihan C, et al. Change in disease burden associated with influenza and air pollutants during the COVID-19 pandemic in Hong Kong. *Digit Health* 2024; **10**: 20552076241261892.

121. Lui KJ, Kendal AP. Impact of influenza epidemics on mortality in the United States from October 1972 to May 1985. *Am J Public Health* 1987; **77**(6): 712-6.
122. Mamelund SE, Iversen BG. [Morbidity and mortality in pandemic influenza in Norway]. *Tidsskr Nor Laegeforen* 2000; **120**(3): 360-3.
123. Markush RE. Crude Mortality in Florida During Asian Influenza Epidemics. *Southern Medical Journal* 1963; **56**(8): 904-13.
124. Matias G, Taylor R, Haguinet F, Schuck-Paim C, Lustig R, Shinde V. Estimates of mortality attributable to influenza and RSV in the United States during 1997-2009 by influenza type or subtype, age, cause of death, and risk status. *Influenza Other Respir Viruses* 2014; **8**(5): 507-15.
125. Mazick A, Gergonne B, Wuillaume F, et al. Higher all-cause mortality in children during autumn 2009 compared with the three previous years: pooled results from eight European countries. *Euro Surveill* 2010; **15**(5).
126. McBean AM, Babish JD, Warren JL. The impact and cost of influenza in the elderly. *Arch Intern Med* 1993; **153**(18): 2105-11.
127. Miller DL, Pereira MS, Clarke M. Epidemiology of the Hong Kong-68 variant of influenza A2 in Britain. *Br Med J* 1971; **1**(5747): 475-9.
128. Moa AM, Menzies RI, Yin JK, MacIntyre CR. Modelling the influenza disease burden in people aged 50-64 and ⩾65 years in Australia. *Influenza Other Respir Viruses* 2022; **16**(1): 132-41.
129. Mølbak K, Widgren K, Jensen KS, et al. Burden of illness of the 2009 pandemic of influenza A (H1N1) in Denmark. *Vaccine* 2011; **29 Suppl 2**: B63-9.
130. Molbak K, Espenhain L, Nielsen J, et al. Excess mortality among the elderly in European countries, December 2014 to February 2015. *Euro Surveill* 2015; **20**(11).
131. Muñoz MP, Soldevila N, Martínez A, et al. Influenza vaccine coverage, influenza-associated morbidity and all-cause mortality in Catalonia (Spain). *Vaccine* 2011; **29**(31): 5047-52.
132. Murray CJ, Lopez AD, Chin B, Feehan D, Hill KH. Estimation of potential global pandemic influenza mortality on the basis of vital registry data from the 1918-20 pandemic: a quantitative analysis. *Lancet* 2006; **368**(9554): 2211-8.
133. Muscatello DJ, Cretikos MA, Macintyre CR. All-cause mortality during first wave of pandemic (H1N1) 2009, New South Wales, Australia, 2009. *Emerg Infect Dis* 2010; **16**(9): 1396-402.
134. Muscatello DJ, Newall AT, Dwyer DE, Macintyre CR. Mortality attributable to seasonal and pandemic influenza, Australia, 2003 to 2009, using a novel time series smoothing approach. *PLoS One* 2014; **8**(6): e64734.
135. Muscatello DJ, Bein KJ, Dinh MM. Emergency Department demand associated with seasonal influenza, 2010 through 2014, New South Wales, Australia. *Western Pac Surveill Response J* 2017; **8**(3): 11-20.
136. Muscatello DJ, Nazareno AL, Turner RM, Newall AT. Influenza-associated mortality in Australia, 2010 through 2019: High modelled estimates in 2017. *Vaccine* 2021; **39**(52): 7578-83.
137. Narayan VV, Iuliano AD, Roguski K, et al. Burden of influenza-associated respiratory and circulatory mortality in India, 2010-2013. *J Glob Health* 2020; **10**(1): 010402.

138. Neuzil KM, Mellen BG, Wright PF, Mitchel EF, Jr., Griffin MR. The effect of influenza on hospitalizations, outpatient visits, and courses of antibiotics in children. *N Engl J Med* 2000; **342**(4): 225-31.
139. Newall AT, Wood JG, Macintyre CR. Influenza-related hospitalisation and death in Australians aged 50 years and older. *Vaccine* 2008; **26**(17): 2135-41.
140. Newall AT, Viboud C, Wood JG. Influenza-attributable mortality in Australians aged more than 50 years: a comparison of different modelling approaches. *Epidemiol Infect* 2010; **138**(6): 836-42.
141. Nicholson KG. Impact of influenza and respiratory syncytial virus on mortality in England and Wales from January 1975 to December 1990. *Epidemiol Infect* 1996; **116**(1): 51-63.
142. Nielsen J, Mazick A, Glismann S, Mølbak K. Excess mortality related to seasonal influenza and extreme temperatures in Denmark, 1994-2010. *BMC Infect Dis* 2011; **11**: 350.
143. Nielsen J, Mazick A, Andrews N, et al. Pooling European all-cause mortality: methodology and findings for the seasons 2008/2009 to 2010/2011. *Epidemiol Infect* 2013; **141**(9): 1996-2010.
144. Nielsen J, Krause TG, Mølbak K. Influenza-associated mortality determined from all-cause mortality, Denmark 2010/11-2016/17: The FluMOMO model. *Influenza Other Respir Viruses* 2018; **12**(5): 591-604.
145. Nielsen J, Vestergaard LS, Richter L, et al. European all-cause excess and influenza-attributable mortality in the 2017/18 season: should the burden of influenza B be reconsidered? *Clin Microbiol Infect* 2019; **25**(10): 1266-76.
146. Nogueira PJ, Nunes B, Machado A, et al. Early estimates of the excess mortality associated with the 2008-9 influenza season in Portugal. *Euro Surveill* 2009; **14**(18).
147. Nørgaard SK, Nielsen J, Schjørring CB, et al. Excess mortality in Europe estimated by EuroMOMO during the COVID-19 pandemic and previous influenza seasons. *Nat Commun* 2026; **17**(1): 1356.
148. Nunes B, Viboud C, Machado A, et al. Excess mortality associated with influenza epidemics in Portugal, 1980 to 2004. *PLoS One* 2011; **6**(6): e20661.
149. Nunes B, Silva S, Rodrigues A, Roquette R, Batista I, Rebelo-de-Andrade H. The 1918-1919 Influenza Pandemic in Portugal: A Regional Analysis of Death Impact. *Am J Epidemiol* 2018; **187**(12): 2541-9.
150. Nygaard IH, Dahal S, Chowell G, Sattenspiel L, Sommerseth HL, Mamelund SE. Age-specific mortality and the role of living remotely: The 1918-20 influenza pandemic in Kautokeino and Karasjok, Norway. *Int J Circumpolar Health* 2023; **82**(1): 2179452.
151. Ohmi K, Marui E. [Estimation of the excess death associated with influenza pandemics and epidemics in Japan after world war II: relation with pandemics and the vaccination system]. *Nihon Koshu Eisei Zasshi* 2011; **58**(10): 867-78.
152. Ortiz JR, Neuzil KM, Cooke CR, Neradilek MB, Goss CH, Shay DK. Influenza pneumonia surveillance among hospitalized adults may underestimate the burden of severe influenza disease. *PLoS One* 2014; **9**(11): e113903.
153. Park M, Wu P, Goldstein E, Joo Kim W, Cowling BJ. Influenza-Associated Excess Mortality in South Korea. *Am J Prev Med* 2016; **50**(4): e111-e9.
154. Pebody RG, Green HK, Andrews N, et al. Uptake and impact of vaccinating school age children against influenza during a season with circulation of drifted influenza A and B strains, England, 2014/15. *Euro Surveill* 2015; **20**(39).

155. Pebody RG, Green HK, Warburton F, et al. Significant spike in excess mortality in England in winter 2014/15 - influenza the likely culprit. *Epidemiol Infect* 2018; **146**(9): 1106-13.
156. Perdelli F. GR, Martino C. [Excess mortality from influenza in Genoa]. *Giornale di Igiene Medicina Preventiva* 1980; **21**(1-2): 21-31.
157. Portugal L. Mortality and Excess Mortality: Improving FluMOMO. *J Environ Public Health* 2021; **2021**: 5582589.
158. Presanis AM, Pebody RG, Paterson BJ, et al. Changes in severity of 2009 pandemic A/H1N1 influenza in England: a Bayesian evidence synthesis. *Bmj* 2011; **343**: d5408.
159. Pumarola T, Díez-Domingo J, Martinón-Torres F, et al. Excess hospitalizations and mortality associated with seasonal influenza in Spain, 2008-2018. *BMC Infect Dis* 2023; **23**(1): 86.
160. Qi L, Li Q, Ding XB, et al. Mortality burden from seasonal influenza in Chongqing, China, 2012-2018. *Hum Vaccin Immunother* 2020; **16**(7): 1668-74.
161. Quandelacy TM, Viboud C, Charu V, Lipsitch M, Goldstein E. Age- and sex-related risk factors for influenza-associated mortality in the United States between 1997-2007. *Am J Epidemiol* 2014; **179**(2): 156-67.
162. Ragona G, Giunchi G, Rocchi G, Muzzi A, De Felici A. Excess mortality from influenza in a large urban population, Rome, Italy, 1956--76. *J Hyg (Lond)* 1978; **80**(2): 249-57.
163. Ramírez AP, Mendoza AR, Montoya JM, et al. [Mortality associated with peak seasons of influenza virus circulation in Bogotá, Colombia, 1997-2005]. *Rev Panam Salud Publica* 2009; **26**(5): 435-9.
164. Ramiro D, Garcia S, Casado Y, Cilek L, Chowell G. Age-specific excess mortality patterns and transmissibility during the 1889-1890 influenza pandemic in Madrid, Spain. *Ann Epidemiol* 2018; **28**(5): 267-72.
165. Reichert TA, Sugaya N, Fedson DS, Glezen WP, Simonsen L, Tashiro M. The Japanese experience with vaccinating schoolchildren against influenza. *N Engl J Med* 2001; **344**(12): 889-96.
166. Reichert TA, Simonsen L, Sharma A, Pardo SA, Fedson DS, Miller MA. Influenza and the winter increase in mortality in the United States, 1959-1999. *Am J Epidemiol* 2004; **160**(5): 492-502.
167. Reyes O, Lee EC, Sah P, Viboud C, Chandra S, Bansal S. Spatiotemporal Patterns and Diffusion of the 1918 Influenza Pandemic in British India. *Am J Epidemiol* 2018; **187**(12): 2550-60.
168. Richard SA, Sugaya N, Simonsen L, Miller MA, Viboud C. A comparative study of the 1918-1920 influenza pandemic in Japan, USA and UK: mortality impact and implications for pandemic planning. *Epidemiol Infect* 2009; **137**(8): 1062-72.
169. Ridenhour BJ, Campitelli MA, Kwong JC, et al. Effectiveness of inactivated influenza vaccines in preventing influenza-associated deaths and hospitalizations among Ontario residents aged ⩾ 65 years: estimates with generalized linear models accounting for healthy vaccinee effects. *PLoS One* 2013; **8**(10): e76318.
170. Rizzo C, Viboud C, Montomoli E, Simonsen L, Miller MA. Influenza-related mortality in the Italian elderly: no decline associated with increasing vaccination coverage. *Vaccine* 2006; **24**(42-43): 6468-75.

171. Rizzo C, Bella A, Viboud C, et al. Trends for influenza-related deaths during pandemic and epidemic seasons, Italy, 1969-2001. *Emerg Infect Dis* 2007; **13**(5): 694-9.
172. Rosano A, Bella A, Gesualdo F, et al. Investigating the impact of influenza on excess mortality in all ages in Italy during recent seasons (2013/14-2016/17 seasons). *Int J Infect Dis* 2019; **88**: 127-34.
173. Rubinson L, Mutter R, Viboud C, et al. Impact of the fall 2009 influenza A(H1N1)pdm09 pandemic on US hospitals. *Med Care* 2013; **51**(3): 259-65.
174. Sánchez-Ramos EL, Monárrez-Espino J, Noyola DE. Impact of vaccination on influenza mortality in children <5years old in Mexico. *Vaccine* 2017; **35**(9): 1287-92.
175. Salto-Quintana JN, Rivera-Alfaro G, Sánchez-Ramos EL, Gómez-Gómez A, Noyola DE. Post-pandemic influenza-associated mortality in Mexico. *Pathog Glob Health* 2019; **113**(2): 67-74.
176. Sattenspiel L, Mamelund SE, Dahal S, Wissler A, Chowell G, Tinker-Fortel E. Death on the permafrost: revisiting the 1918-1920 influenza pandemic in Alaska using death certificates. *Am J Epidemiol* 2025; **194**(1): 152-61.
177. Schanzer DL, Tam TW, Langley JM, Winchester BT. Influenza-attributable deaths, Canada 1990-1999. *Epidemiol Infect* 2007; **135**(7): 1109-16.
178. Schanzer DL, Langley JM, Tam TW. Co-morbidities associated with influenza-attributed mortality, 1994-2000, Canada. *Vaccine* 2008; **26**(36): 4697-703.
179. Schanzer DL, Sevenhuysen C, Winchester B, Mersereau T. Estimating influenza deaths in Canada, 1992-2009. *PLoS One* 2013; **8**(11): e80481.
180. Schanzer DL, Schwartz B. Impact of seasonal and pandemic influenza on emergency department visits, 2003-2010, Ontario, Canada. *Acad Emerg Med* 2013; **20**(4): 388-97.
181. Schindler CJA, Wittenberg I, Damm O, Kramer R, Mikolajczyk R, Schönfelder T. Influenza-Associated Excess Mortality and Hospitalization in Germany from 1996 to 2018. *Infect Dis Ther* 2024; **13**(11): 2333-50.
182. Schmidt SSS, Iuliano AD, Vestergaard LS, et al. All-cause versus cause-specific excess deaths for estimating influenza-associated mortality in Denmark, Spain, and the United States. *Influenza Other Respir Viruses* 2022; **16**(4): 707-16.
183. Shindo N. [Morbidity and mortality of influenza in Japan]. *Nihon Rinsho* 2000; **58**(11): 2187-91.
184. Shrestha SS, Swerdlow DL, Borse RH, et al. Estimating the burden of 2009 pandemic influenza A (H1N1) in the United States (April 2009-April 2010). *Clin Infect Dis* 2011; **52 Suppl 1**: S75-82.
185. Simonsen L, Clarke MJ, Williamson GD, Stroup DF, Arden NH, Schonberger LB. The impact of influenza epidemics on mortality: introducing a severity index. *Am J Public Health* 1997; **87**(12): 1944-50.
186. Simonsen L, Reichert TA, Viboud C, Blackwelder WC, Taylor RJ, Miller MA. Impact of influenza vaccination on seasonal mortality in the US elderly population. *Arch Intern Med* 2005; **165**(3): 265-72.
187. Simonsen L, Spreeuwenberg P, Lustig R, et al. Global mortality estimates for the 2009 Influenza Pandemic from the GLaMOR project: a modeling study. *PLoS Med* 2013; **10**(11): e1001558.
188. Sinnathamby MA, Warburton F, Reynolds AJ, et al. An intercountry comparison of the impact of the paediatric live attenuated influenza vaccine (LAIV) programme across the UK

and the Republic of Ireland (ROI), 2010 to 2017. *Influenza Other Respir Viruses* 2023; **17**(2): e13099.
189. Sinnecker H. BW, Woratz G., Muller D., Bigl S., Bog R., Grimm J., Hajduk F. [Investigations into excess mortality and into the effectiveness of prophylactic influenza vaccinations in patients with chronic cardiovascular disease.]. *Zeitschrift fur Klinische Medizin* 1987; **42**(26): 2353-7.
190. Somayaji R, Neradilek MB, Szpiro AA, et al. Effects of Air Pollution and Other Environmental Exposures on Estimates of Severe Influenza Illness, Washington, USA. *Emerg Infect Dis* 2020; **26**(5): 920-9.
191. Sprenger MJ, Mulder PG, Beyer WE, Van Strik R, Masurel N. Impact of influenza on mortality in relation to age and underlying disease, 1967-1989. *Int J Epidemiol* 1993; **22**(2): 334-40.
192. Staub K, Panczak R, Matthes KL, et al. Historically High Excess Mortality During the COVID-19 Pandemic in Switzerland, Sweden, and Spain. *Ann Intern Med* 2022; **175**(4): 523-32.
193. Steele LE, Tilstra AM, Short KR, Mamelund SE, Wrigley-Field E. Age-specific mortality patterns across influenza pandemics: evidence from all-cause mortality data across multiple populations. *Int J Epidemiol* 2026; **55**(3).
194. Stein AN, Pendrey CGA, Muscatello DJ, et al. Estimates of Seasonal Influenza Burden That Could Be Averted by Improved Influenza Vaccines in the Australian Population Aged Under 65 Years, 2015-2019. *Influenza Other Respir Viruses* 2024; **18**(4): e13289.
195. Stroup DF, Thacker SB, Herndon JL. Application of multiple time series analysis to the estimation of pneumonia and influenza mortality by age 1962-1983. *Stat Med* 1988; **7**(10): 1045-59.
196. Sugaya N, Takeuchi Y. Mass vaccination of schoolchildren against influenza and its impact on the influenza-associated mortality rate among children in Japan. *Clin Infect Dis* 2005; **41**(7): 939-47.
197. Suter J, Devos I, Matthes KL, Staub K. The health and demographic impacts of the "Russian flu" pandemic in Switzerland in 1889/1890 and in the years thereafter. *Epidemiol Infect* 2024; **152**: e174.
198. Tachibana T, Kawaminami K, Minowa M. [Excess mortality from influenza epidemics in Japan, 1980-1994]. *Nihon Koshu Eisei Zasshi* 1999; **46**(4): 263-74.
199. Takahashi M, Tango T. [Comparative study of new method and the Kawai and Fukutomi methods for estimating excess mortality associated with influenza-epidemics, based upon national vital statistics from 1975 to 1997]. *Nihon Koshu Eisei Zasshi* 2001; **48**(10): 816-26.
200. Takahashi M, Tango T. [Estimation of excess mortality associated with influenza-epidemics by age and cause specific death in Japan, 1975-1999]. *Nihon Eiseigaku Zasshi* 2002; **57**(3): 571-84.
201. Takahashi M. [Estimation of the range of excess death associated with influenza epidemics: application of a model using annual mortality rates and the seasonal index for determining minimum excess mortality estimates]. *Nihon Koshu Eisei Zasshi* 2006; **53**(8): 554-62.
202. Takahashi M, Nagai M. [Estimation of excess mortality associated with influenza epidemics specific for sex, age and cause of death in Japan during 1987-2005]. *Nihon Eiseigaku Zasshi* 2008; **63**(1): 5-19.

203. Tayback M, Reyes AC. Philippine influenza epidemic of 1957. *Public Health Rep (1896)* 1957; **72**(10): 855-60.
204. Tempia S, Walaza S, Viboud C, et al. Deaths associated with respiratory syncytial and influenza viruses among persons ≥5 years of age in HIV-prevalent area, South Africa, 1998-2009(1). *Emerg Infect Dis* 2015; **21**(4): 600-8.
205. Tempia S, Walaza S, Moyes J, et al. Quantifying How Different Clinical Presentations, Levels of Severity, and Healthcare Attendance Shape the Burden of Influenza-associated Illness: A Modeling Study From South Africa. *Clin Infect Dis* 2019; **69**(6): 1036-48.
206. Thiberville SD, Gaudart J, Raoult D, Charrel RN. Influenza-attributable deaths in south-eastern France (1999 to 2010): mortality predictions were undependable. *BMC Public Health* 2015; **15**: 539.
207. Thompson WW, Shay DK, Weintraub E, et al. Mortality associated with influenza and respiratory syncytial virus in the United States. *Jama* 2003; **289**(2): 179-86.
208. Thompson WW, Shay DK, Weintraub E, et al. Influenza-associated hospitalizations in the United States. *Jama* 2004; **292**(11): 1333-40.
209. Thompson WW, Weintraub E, Dhankhar P, et al. Estimates of US influenza-associated deaths made using four different methods. *Influenza Other Respir Viruses* 2009; **3**(1): 37-49.
210. Tillett HE, Smith JW, Clifford RE. Excess morbidity and mortality associated with influenza in England and Wales. *Lancet* 1980; **1**(8172): 793-5.
211. Tillett HE, Smith JW, Gooch CD. Excess deaths attributable to influenza in England and Wales: age at death and certified cause. *Int J Epidemiol* 1983; **12**(3): 344-52.
212. Togashi T, Matsuzono Y, Narita M, Morishima T. Influenza-associated acute encephalopathy in Japanese children in 1994-2002. *Virus Res* 2004; **103**(1-2): 75-8.
213. Tominaga M. YK, Hara M. Excess mortality from diabetes mellitus during influenza epidemics, 1961/1974 in Japan (Japanese). *Journal of the Japan Diabetes Society* 1977; **20**(2): 174-9.
214. Estimates of deaths associated with seasonal influenza --- United States, 1976-2007. *MMWR Morb Mortal Wkly Rep* 2010; **59**(33): 1057-62.
215. van Asten L, van den Wijngaard C, van Pelt W, et al. Mortality attributable to 9 common infections: significant effect of influenza A, respiratory syncytial virus, influenza B, norovirus, and parainfluenza in elderly persons. *J Infect Dis* 2012; **206**(5): 628-39.
216. van Asten L, Harmsen CN, Stoeldraijer L, et al. Excess Deaths during Influenza and Coronavirus Disease and Infection-Fatality Rate for Severe Acute Respiratory Syndrome Coronavirus 2, the Netherlands. *Emerg Infect Dis* 2021; **27**(2): 411-20.
217. Vestergaard LS, Nielsen J, Krause TG, et al. Excess all-cause and influenza-attributable mortality in Europe, December 2016 to February 2017. *Euro Surveill* 2017; **22**(14).
218. Viboud C, Grais RF, Lafont BA, Miller MA, Simonsen L. Multinational impact of the 1968 Hong Kong influenza pandemic: evidence for a smoldering pandemic. *J Infect Dis* 2005; **192**(2): 233-48.
219. Viboud C, Tam T, Fleming D, Miller MA, Simonsen L. 1951 influenza epidemic, England and Wales, Canada, and the United States. *Emerg Infect Dis* 2006; **12**(4): 661-8.
220. Viboud C, Simonsen L, Fuentes R, Flores J, Miller MA, Chowell G. Global Mortality Impact of the 1957-1959 Influenza Pandemic. *J Infect Dis* 2016; **213**(5): 738-45.

221. Walkowiak MP, Walkowiak D. From respiratory diseases to nervous system disorders: Unraveling the certified causes of influenza-associated deaths in Poland from 2000 to 2019. *Influenza Other Respir Viruses* 2023; **17**(11): e13214.
222. Wang H, Fu C, Li K, et al. Influenza associated mortality in Southern China, 2010-2012. *Vaccine* 2014; **32**(8): 973-8.
223. Warren-Gash C, Bhaskaran K, Hayward A, et al. Circulating influenza virus, climatic factors, and acute myocardial infarction: a time series study in England and Wales and Hong Kong. *J Infect Dis* 2011; **203**(12): 1710-8.
224. Wijngaard CC, Asten L, Koopmans MP, et al. Comparing pandemic to seasonal influenza mortality: moderate impact overall but high mortality in young children. *PLoS One* 2012; **7**(2): e31197.
225. Wong CM, Chan KP, Hedley AJ, Peiris JS. Influenza-associated mortality in Hong Kong. *Clin Infect Dis* 2004; **39**(11): 1611-7.
226. Wong CM, Yang L, Chan E, Chan KH, Hedley AJ, Peiris JS. Influenza-associated hospitalisation. *Hong Kong Med J* 2009; **15 Suppl 9**: 35-7.
227. Wong CM, Peiris JS, Yang L, et al. Effect of influenza on cardiorespiratory and all-cause mortality in Hong Kong, Singapore and Guangzhou. *Hong Kong Med J* 2012; **18 Suppl 2**: 8-11.
228. Wong JY, Wu P, Nishiura H, et al. Infection fatality risk of the pandemic A(H1N1)2009 virus in Hong Kong. *Am J Epidemiol* 2013; **177**(8): 834-40.
229. Wong JY, Goldstein E, Fang VJ, Cowling BJ, Wu P. Real-time estimation of the influenza-associated excess mortality in Hong Kong. *Epidemiol Infect* 2019; **147**: e217.
230. Wong JY, Cheung JK, Iuliano AD, Wu P, Cowling BJ. Influenza-associated Excess Mortality Associated With Influenza B in Hong Kong, 2014-2023. *J Infect Dis* 2026; **233**(3): 485-92.
231. Wu P, Goldstein E, Ho LM, et al. Excess mortality impact of two epidemics of pandemic influenza A(H1N1pdm09) virus in Hong Kong. *Influenza Other Respir Viruses* 2014; **8**(1): 1-7.
232. Wu S, Wei Z, Greene CM, et al. Mortality burden from seasonal influenza and 2009 H1N1 pandemic influenza in Beijing, China, 2007-2013. *Influenza Other Respir Viruses* 2018; **12**(1): 88-97.
233. Yang L, Wong CM, Chan KP, et al. Seasonal effects of influenza on mortality in a subtropical city. *BMC Infect Dis* 2009; **9**: 133.
234. Yang L, Ma S, Chen PY, et al. Influenza associated mortality in the subtropics and tropics: results from three Asian cities. *Vaccine* 2011; **29**(48): 8909-14.
235. Yang L, Wang XL, Chan KP, et al. Hospitalisation associated with the 2009 H1N1 pandemic and seasonal influenza in Hong Kong, 2005 to 2010. *Euro Surveill* 2012; **17**(45).
236. Yang L, Chan KP, Cowling BJ, et al. Excess mortality associated with the 2009 pandemic of influenza A(H1N1) in Hong Kong. *Epidemiol Infect* 2012; **140**(9): 1542-50.
237. Yang L, Chan KP, Wong CM, et al. Comparison of influenza disease burden in older populations of Hong Kong and Brisbane: the impact of influenza and pneumococcal vaccination. *BMC Infect Dis* 2019; **19**(1): 162.
238. Yap FH, Ho PL, Lam KF, Chan PK, Cheng YH, Peiris JS. Excess hospital admissions for pneumonia, chronic obstructive pulmonary disease, and heart failure during influenza seasons in Hong Kong. *J Med Virol* 2004; **73**(4): 617-23.

239. Yin TL, Chen N, Zhang JY, et al. Excess multi-cause mortality linked to influenza virus infection in China, 2012-2021: a population-based study. *Front Public Health* 2024; **12**: 1399672.
240. Yu H, Feng L, Viboud CG, et al. Regional variation in mortality impact of the 2009 A(H1N1) influenza pandemic in China. *Influenza Other Respir Viruses* 2013; **7**(6): 1350-60.
241. Zhang H, Xiong Q, Wu P, Chen Y, Leung NHL, Cowling BJ. Influenza-associated mortality in Yancheng, China, 2011-15. *Influenza Other Respir Viruses* 2018; **12**(1): 98-103.
242. Zhao MJ, He YL, Chen J, et al. [Estimates of influenza-associated excess mortality by three regression models in Shanxi Province during 2013-2017]. *Zhonghua Yu Fang Yi Xue Za Zhi* 2019; **53**(10): 1012-7.
243. Zhou H, Thompson WW, Viboud CG, et al. Hospitalizations associated with influenza and respiratory syncytial virus in the United States, 1993-2008. *Clin Infect Dis* 2012; **54**(10): 1427-36.
244. Ziegler E, Matthes KL, Middelkamp PW, et al. Retrospective modelling of the disease and mortality burden of the 1918-1920 influenza pandemic in Zurich, Switzerland. *Epidemics* 2025; **50**: 100813.
245. Zucs P, Buchholz U, Haas W, Uphoff H. Influenza associated excess mortality in Germany, 1985-2001. *Emerg Themes Epidemiol* 2005; **2**: 6.
